# Reduced cue-induced reinstatement of cocaine-seeking behavior in *Plcb1* +/− mice

**DOI:** 10.1038/s41398-021-01396-6

**Published:** 2021-10-11

**Authors:** Judit Cabana-Domínguez, Elena Martín-García, Ana Gallego-Roman, Rafael Maldonado, Noèlia Fernàndez-Castillo, Bru Cormand

**Affiliations:** 1 Department de Genètica, Microbiologia i Estadística, Facultat de Biologia, Universitat de Barcelona, Barcelona, Catalonia Spain; 2 Centro de Investigación Biomédica en Red de Enfermedades Raras (CIBERER), Madrid, Spain; 3 Institut de Biomedicina de la Universitat de Barcelona (IBUB), Barcelona, Catalonia Spain; 4 Institut de Recerca Sant Joan de Déu (IR-SJD), Barcelona, Catalonia Spain; 5 Laboratory of Neuropharmacology-Neurophar, Department of Experimental and Health Sciences, Universitat Pompeu Fabra (UPF), Barcelona, Catalonia Spain; 6 Hospital del Mar Medical Research Institute (IMIM), Barcelona, Catalonia Spain

**Keywords:** Neuroscience, Addiction, Genomics

## Abstract

Cocaine addiction causes serious health problems, and no effective treatment is available yet. We previously identified a genetic risk variant for cocaine addiction in the *PLCB1* gene and found this gene upregulated in postmortem brains of cocaine abusers and in human dopaminergic neuron-like cells after an acute cocaine exposure. Here, we functionally tested the contribution of the *PLCB1* gene to cocaine addictive properties using *Plcb1*+/− mice. First, we performed a general phenotypic characterization and found that *Plcb1*+/− mice showed normal behavior, although they had increased anxiety and impaired short-term memory. Subsequently, mice were trained for operant conditioning, self-administered cocaine for 10 days, and were tested for cocaine motivation. After extinction, we found a reduction in the cue-induced reinstatement of cocaine-seeking behavior in *Plcb1*+/− mice. After reinstatement, we identified transcriptomic alterations in the medial prefrontal cortex of *Plcb1*+/− mice, mostly related to pathways relevant to addiction like the dopaminergic synapse and long-term potentiation. To conclude, we found that heterozygous deletion of the *Plcb1* gene decreases cue-induced reinstatement of cocaine-seeking, pointing at PLCB1 as a possible therapeutic target for preventing relapse and treating cocaine addiction.

## Introduction

Cocaine is the most used psychostimulant illicit drug worldwide [1], causing severe health problems that include the development of cocaine addiction in around 15–16% of cocaine users [2]. Cocaine addiction is a complex psychiatric disorder that results from the interaction of genetic, epigenetic, and environmental risk factors [3]. The heritability of cocaine addiction is one of the highest among psychiatric disorders, estimated around 65% for women [4] and 79% for men [5]. However, the genetic factors and mechanisms that underlie the transition from drug use to addiction and its establishment remain unknown.

In a previous study, we identified a single nucleotide polymorphism in the *PLCB1* gene associated with drug dependence (rs1047383), and especially with a subgroup of cocaine-addicted patients, that was replicated in an independent clinical sample [6]. Also, genetic variants in this gene were found nominally associated with an illegal substance and cocaine addiction in two GWAS performed in European-American samples [7]. On the other hand, we found that cocaine increased the expression of *PLCB1* both in human dopaminergic neuron-like cells (differentiated SH-SY5Y cells) after acute cocaine exposure and in postmortem samples of the nucleus accumbens (NAc) of cocaine abusers [6]. Interestingly, this gene was also found over-expressed in the same brain region in mice after cocaine administration for 7 days and also during withdrawal [8]. All this evidence suggest that *PLCB1* may play a role in cocaine addiction.

The *PLCB1* gene encodes phospholipase C beta 1, and it is highly expressed in the brain, mainly in the frontal cortex, basal ganglia (caudate, putamen, and NAc) and hippocampus (HPC). These brain regions are crucial for drug reward and the formation of drug-context associations, both contributing to the development and maintenance of addiction [9–14]. Several neurotransmitters activate this protein, including dopamine through DRD1 and DRD2 [15, 16], serotonin by 5-HT2A and 2C receptors [17, 18] and glutamate by mGluR1 [19, 20]. Thus, PLCB1 might be a point of convergence of neurotransmitter systems that play an essential role in the development of addiction (recently reviewed [21, 22]). The activation of PLCB1 produces the cleavage of phosphatidylinositol 4,5-bisphosphate (PIP2) into the second messengers diacylglycerol and inositol 1,4,5-trisphosphate (IP3), responsible for intracellular signal transduction. Alterations in PLCB1-mediated signaling in the brain have been associated with other neuropsychiatric disorders such as epilepsy, schizophrenia, and bipolar disorder [23].

Here we studied the contribution of the *PLCB1* gene to cocaine addiction and dissected its participation in the different aspects of the addictive process. We used heterozygous knockout (KO) mice (*Plcb1*+/−), as the homozygous KO (*Plcb1*−/−) showed seizure attacks and low viability after birth [24]. In addition, the use of a constitutive heterozygous KO mouse model allowed us to assess *Plcb1* haploinsufficiency during neurodevelopment in mice similarly to humans, where inherited genetic risk or protective variants can modulate the susceptibility to addiction [6]. First, we performed a general phenotypic characterization using a battery of behavioral tests, including memory, anxiety, locomotor activity, coordination, food and water intake, and sucrose preference. Then, we evaluated cocaine operant self-administration, extinction, and cue-induced reinstatement. Finally, we studied transcriptional alterations to further understand the molecular mechanisms involved.

## Methods

### Animals

Male mice, 8 weeks old, were housed individually at temperature- and humidity-controlled laboratory conditions (21 ± 1 °C, 55 ± 10%) maintained with food and water ad libitum. Mice were tested during the dark phase of a reverse light cycle (lights off at 8.00 h and on at 20.00 h). *Plcb1+/*− mice in a C57BL/6J background and their wild-type (WT) littermates were used [24]. All experimental protocols were performed in accordance with the guidelines of the European Communities Council Directive 2010/63/EU and approved by the local ethics committee (Comitè Ètic d’Experimentació Animal-Parc de Recerca Biomèdica de Barcelona, CEEA-PRBB, agreement N°9213). In agreement, maximal efforts were made to reduce the suffering and the number of mice used.

### Western blot against Plcb1

Medial prefrontal cortex (mPFC) and HPC from mice not exposed to cocaine (*n* = 4 WT and 4 *Plcb1*+/− mice) were dissected. Protein lysates and western blot experiments were performed as previously described [25] using a primary antibody against Plcb1 (sc-5291, Santa Cruz Biotechnology, Texas, USA) diluted 1:500, and a secondary anti-mouse IgG antibody (A0545, Sigma-Aldrich, UK) diluted 1:10,000. For normalization, α-tubulin levels were measured with an anti-α-tubulin antibody (T5168, Sigma-Aldrich, UK; diluted 1:2.000).

### Behavioral tests for phenotype characterization

**Fig. 1:**
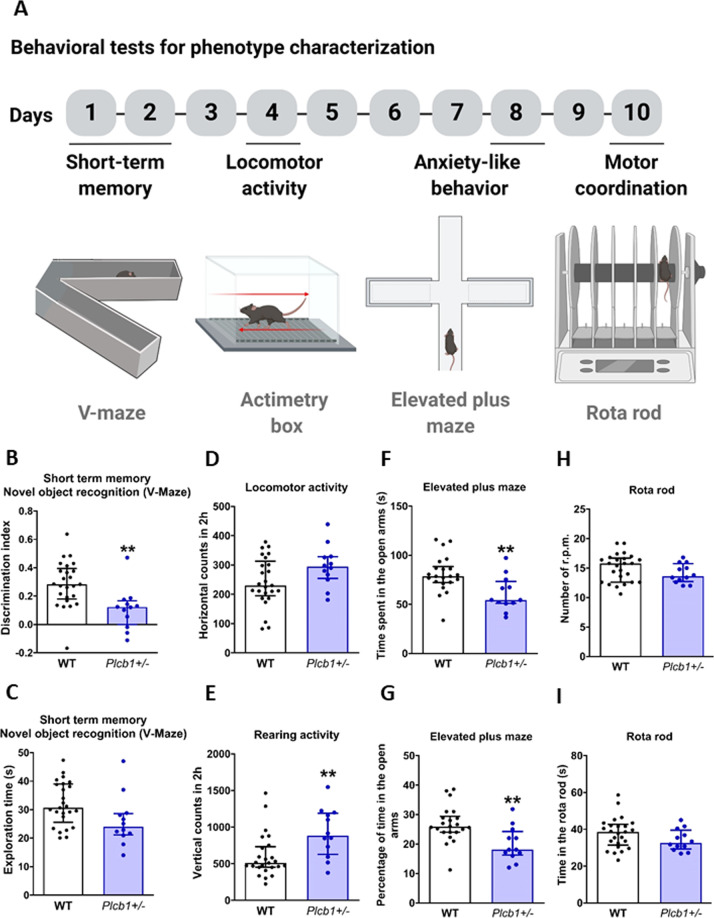
Behavioral tests for phenotype characterization. **A** Timeline of the experimental sequence. **B**, **C** Short-term memory measured in the V-maze for novel object recognition task, (**B**) discrimination index (*t*-test, **P < 0.01 vs WT) and (**C**) exploration. **D** Locomotor and (**E**) rearing activity measured in actimetry boxes (*U*-Mann–Whitney, **P < 0.01 vs WT). **F** Anxiety-like behavior measured by the time spent in the open arms (*t*-test, **P < 0.01 vs WT) and (**G**) the percentage of time in the open arms in the elevated plus maze (*t*-test, **P < 0.01 vs WT). Motor coordination measured in the rota rod test by the (**H**) number of r.p.m. that the mice performed in the average trials or the (**I**) mean time they remain in the apparatus. All data are expressed in median and interquartile range and individual data are shown (WT *n* = 25; *Plcb1*+/− *n* = 12).

A group of 25 WT and 12 *Plcb1*+/− mice underwent different behavioral tests for phenotype characterization for 10 days, as described in Fig. 1. Mice were first tested for short-term memory of a novel object recognition task on a V-maze apparatus. After this test, locomotor activity was evaluated in actimetry boxes. Anxiety-like behavior was then assessed by using the elevated plus maze test, and finally motor coordination was evaluated using the rota rod test. In addition, six animals per genotype were placed in experimental PHECOMP boxes (Panlab and Harvard Apparatus) to control several consummatory and locomotor parameters. Every day position bottles was exchanged, and the consumption was measured after a 24 h interval.

### Operant conditioning maintained by cocaine

Cocaine self-administration experiments were performed in the same animals as previously described [26, 27], after the behavioral tests for phenotype characterization. Each 2 h daily self-administration session started with a priming injection of the drug. Cocaine was intravenously infused in 23.5 μl over 2 s (0.5 mg/kg per injection). Cue light, located above the active hole, was paired with the delivery of the reinforcer. Mice (WT *n* = 36; *Plcb1*+/−*n* = 26) were trained under a fixed ratio 1 schedule of reinforcement (FR1; one nose-poke lead to the delivery of one dose of cocaine) over five consecutive daily sessions and under a fixed ratio 3 (FR3) over five consecutive daily sessions. Control mice trained with saline were included for both genotypes (WT *n* = 6; *Plcb1*+/−*n* = 6). After the ten FR sessions, animals were tested in a progressive ratio schedule of 4 h where the response requirement to earn the cocaine escalated according to the following series: 1–2–3–5–12–18–27–40–60–90–135–200–300–450–675–1000. Then, we proceed with the extinction phase, 2 h daily sessions following the same experimental conditions than the cocaine self-administration sessions except that cocaine was not available, and cue-light was not presented. Finally, mice that accomplished extinction criterion were tested in the cue-induced reinstatement during a 2 h session, to evaluate the reinstatement of cocaine-seeking behavior.

After the cue-induced reinstatement, animals were euthanized by decapitation, brains were quickly removed, and the mPFC and HPC were dissected. Brain tissues were then frozen by immersion in 2‐methylbutane surrounded by dry ice and stored at −80 °C for later RNA isolation and transcriptomic analyses.

### RNA extraction and RNA sequencing

Total RNA of 12 WT and 11 *Plcb1*+/− mice from mPFC and HPC were isolated using the RNeasy Lipid Tissue Mini Kit (Qiagen Düsseldorf, Germany) according to the manufacturer’s protocol. RNA samples were grouped in four pools consisting of three mice per pool for each experimental group. Pools were homogeneous in the average number of nose pokes in the cue-induced reinstatement and representative from the whole sample. Furthermore, no significant differences were observed in the mean number of cocaine infusions in 2 h sessions among pools with an average of 28.5 ± 0.64 infusions in Plcb1+/− mutants and in 26.48 ± 1.23 in WT, suggesting that any transcriptomic change found would be related to the addiction phenotype and not to the concentration of cocaine in the brain in the previous phase of acquisition (*t*-test = 1.42, n.s.).

RNA sequencing (RNAseq) was performed by the Centre de Regulació Genòmica (CRG, Barcelona, Spain). Libraries were prepared using the TruSeq Stranded mRNA Sample Prep Kit_v2 (Illumina, San Diego, CA, USA) according to the manufacturer’s protocol and sequenced 2 × 75 on Illumina’s HiSeq3000 system for both mPFC and HPC. The Bioinformatics service of CRG carried out the analysis of RNAseq. Briefly, FastQC v0.11.5 [28] was used to inspect the reads quality and CutAdapt 1.7.1 [29] to clean the data of adapters and low-quality reads. Then, reads were mapped to the *Mus musculus* genome of reference (GRCm38/mm10) with STAR 2.5.3a [30], and the differential expression analysis was done by DESeq2 [31] to compare WT and *Plcb1*+/−. Corrections for multiple testing were applied by adjusting the *p* values with a 5% False Discovery Rate.

### Functional annotation of RNAseq results

We performed a functional group enrichment of differentially expressed genes (DEGs) in mPFC using the DAVID Annotation Tool (http://david.abcc.ncifcrf.gov) [32] considering GO (Gene Ontology) biological processes and KEGG pathways (Kyoto Encyclopedia of Genes and Genomes). Then, we searched for over‐represented transcription factor‐binding sites (TFBS) using the information of MsigDB (https://www.gsea-msigdb.org/gsea/msigdb) integrated on WebGestalt2019 (http://www.webgestalt.org/) [33], and the default parameters, applying the weighted set cover method to reduce redundancy. In both analyses, the Benjamin–Hochberg procedure was performed for multiple testing. Finally, we investigated the existence of gene networks with Ingenuity Pathway Analysis 8.8 software (IPA, http://www.ingenuity.com/products/ipa; Ingenuity Systems, Redwood City, CA, USA) [34] after selecting genes with fold-change (FC) > |1.2 | .

### Statistical analysis

Mice were randomly allocated in their experimental groups and experiments were performed under blind conditions. Three-way ANOVA with repeated measures was used to test the evolution over sessions or days. Sessions or days were used as within-subject factors and genotype (*Plcb1*+/− or WT) and drug (cocaine or saline) were used as between-subjects factors. Post-hoc analyses (Newman–Keuls) were performed when required. Comparisons between two groups were analyzed by Student *t* test or *U*-Mann–Whitney depending on the distribution defined by the Kolmogorov–Smirnov normality test and the sample size. The chi-square analyses were performed to compare the percentage of mice that acquired operant learning criteria in the different experimental groups. Results are expressed as mean ± SEM or individual values with the median and the interquartile range specified in the figure legend. Differences were considered significant at *P* < 0.05. The sample size was calculated based on power analysis using two-sample Student *t* test, achieving a power between 80 and 90%. The statistical analyses were performed using the Statistical Package for Social Science tool SPSS^®^25.0 (SPSS Inc, Chicago, USA).

See supplementary information for more details of genotyping of transgenic mice, drugs, behavioral tests of phenotype characterization, operant conditioning maintained by cocaine, RNA extraction and sequencing.

## Results

In the present work, we aimed to assess the contribution of the *PLCB1* gene in the different stages of cocaine addiction using heterozygous KO mice (*Plcb1*+/−). To do so, we performed a general phenotypic characterization and, then we evaluated cocaine operant self-administration, extinction, and cue-induced reinstatement in those animals. Also, we studied transcriptional changes in mPFC and HPC to understand the mechanisms involved.

### Behavioral tests for phenotype characterization

First, we confirmed by western blot that protein levels of Plcb1 were reduced in the brain regions of interest, about 22%, on the *Plcb1*+/− mice compared to WT in mPFC and HPC (*P* = 0.013 and *P* = 0.018, respectively; Supplementary Fig. S1). Importantly, this reduction was observed in Plcb1a isoform but not in Plcb1b, both in mPFC and HPC, and there seems to be a slight compensatory mechanism.

Then we evaluated the effects of the heterozygous deletion of *Plcb1* on general behavioral responses, including locomotor, cognitive and emotional responses, food, and water intake. *Plcb1*+/− mice showed a lower discrimination index in the novel object recognition compared to WT (*t*-test = 3.21, *P* < 0.01, Fig. 1B), suggesting an impairment in short-term memory. This difference was not influenced by exploration time, as this variable was equal between genotypes (Fig. 1C). Furthermore, no differences in locomotor activity were reported between genotypes, discarding an involvement of the *Plcb1* heterozygous deletion in locomotion (Fig. 1D). Mutant mice showed increased exploratory activity with a higher number of rearings than the WT mice (*U*-Mann–Whitney = 67.5, *P* < 0.01, Fig. 1E). An anxiogenic profile was revealed in the elevated plus maze in mutants, as shown by the reduced time spent in the open arms (*t*-test = 3.02, *P* < 0.001, Fig. 1F) and the percentage of time (*t*-test = 3.16, *P* < 0.001, Fig. 1G). Finally, the rota rod test revealed that the heterozygous deletion of *Plcb1* did not affect motor coordination (Fig. 1H, I).

Several consummatory and locomotor parameters were long-term monitored in the PheComp boxes (Supplementary Fig. 2A–H, *n* = 6 per genotype). No differences between genotypes were revealed during the whole experimental period in body weight, food, and water intake, levels of sucrose preference, stereotyped movements, horizontal and vertical locomotor activity, indicating no altered behavior in the *Plcb1*+/− mice.

### Operant conditioning maintained by cocaine

**Fig. 2:**
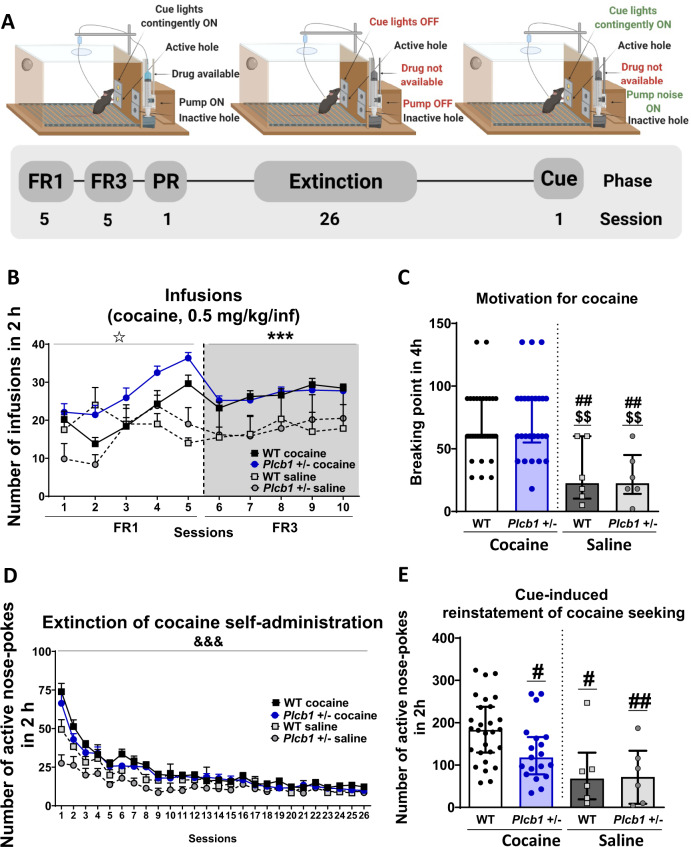
Operant conditioning maintained by cocaine in *Plcb1*+/−, wild-type (WT) and corresponding control saline mice. **A** Timeline of the experimental sequence. **B** The number of cocaine infusions (0.5 mg/kg/infusion) in both genotypes increased progressively across sessions during FR1 (repeated measures ANOVA, interaction between genotype × drug × sessions, ^✩^*P* < 0.05) and remained stable during FR3 and higher in mice trained with cocaine (repeated measures ANOVA, main effect of drug, ***P < 0.001). The maximum number of infusions was reached on session 5 in both genotypes and was slightly superior in *Plcb1*+/− (36.35 ± 1.47) than in WT (29.61 ± 2.23) but post-hoc analyses demonstrated that this difference was not significant. **C** The motivation for cocaine was equivalent in both genotypes trained with cocaine but higher than those trained with saline (individual data with interquartile range, *U*-Mann–Whitney, ^##^*P* < 0.01 vs WT cocaine, ^$$^*P* < 0.01 vs *Plcb1*+/− cocaine). **D** Both genotypes also showed similar levels of extinction that decreased during sessions, but the curve was more pronounced in WT mice than in mutants (repeated measures ANOVA, interaction genotype × sessions, ^&&&^*P* < 0.001). **E** Decreased cue-induced reinstatement of cocaine-seeking in *Plcb*+/− mice was obtained compared to WT (individual data with interquartile range, *U*-Mann–Whitney, ^#^*P* < 0.05, ^##^*P* < 0.01 vs WT cocaine). All data are expressed in mean ± SEM when sessions are represented or median and interquartile range when individual data are shown (WT cocaine *n* = 28-36; *Plcb1*+/− cocaine *n* = 19–26; saline *n* = 6 per genotype). Statistical details are included in Supplementary Table S1.

Then, we investigated the effects of the heterozygous deletion of *Plcb1* in cocaine behavioral responses related to its addictive properties. For this purpose, *Plcb1*+/− mice and their WT littermates were trained for cocaine operant self-administration (0.5 mg/kg/infusion) during FR1 and FR3, progressive ratio, extinction and cue-induced reinstatement (Fig. 2A). Control mice trained with saline were included for both genotypes. Results showed that the percentage of mice reaching the criteria of operant conditioning was 100% for both genotypes trained with cocaine and 33% for mice trained with saline: [*Chi-square* test = 9.50; *P* < 0.01, *Plcb1*+/− cocaine vs *Plcb1*+/− saline] and [*Chi-square* test = 14.00; *P* < 0.001, WT cocaine vs WT saline], as expected.

The primary reinforcing effects and the motivation for cocaine were similar in both genotypes (Fig. 2B, C). During FR1, both genotypes similarly increased the number of cocaine infusions across sessions, whereas mice trained with saline remained steady (repeated measures ANOVA, interaction between genotype × drug × sessions, *P* < 0.05, Fig. 2B and Supplementary Table S1). Thus, the evolution of operant responding was different in mice trained with cocaine and saline, with an increased responding over nearly all sessions in cocaine-trained mice. This enhancement in cocaine responding was more pronounced in mutants than WT mice. Indeed, WT mice showed a decrease in cocaine intake in the second session. The maximum number of infusions was reached on session 5 in both genotypes and was slightly superior in *Plcb1*+/− mutants (36.35 ± 1.47) than in WT (29.61 ± 2.23) but post-hoc analyses demonstrated that this difference in cocaine intake was not significant (Fig. 2B). Similar findings were observed for the total number of nose-pokes in which operant responding increased in both genotypes with cocaine but remained stable with saline (Supplementary Fig. S3A). When the effort to obtain one dose of cocaine increased to FR3, the number of infusions was stable across sessions in WT and *Plcb1*+/− mice. Similarly, operant responding was higher for all groups than in FR1 with stable higher levels of responding (Supplementary Fig. S2A) and a higher number of infusions (repeated measures ANOVA, the main effect of the drug, *P* < 0.001, Fig. 2B) in mice trained with cocaine than with saline, independently of the genotype.

Motivation for cocaine was evaluated in a progressive ratio schedule, and no significant differences were obtained between genotypes (Fig. 2C). The levels of extinction of the operant behavior were similar between genotypes and decreased progressively across sessions (repeated measures ANOVA, interaction genotype × sessions, *P* < 0.001, Fig. 2D). The percentage of mice reaching cocaine-seeking extinction criteria was similar in *Plcb1*+/− (73%) and WT (78%) mice.

Importantly, *Plcb1*+/− mice showed significantly reduced cue-induced reinstatement of cocaine-seeking compared to WT mice (*U*-Mann–Whitney, *P* < 0.05, Fig. 2E), with 27.59% less active nose-pokes compared to WT mice trained with cocaine. Furthermore, mice trained with saline from both genotypes exhibited 55.84% reduction of active nose-pokes than WT mice trained with cocaine and 39.20% less than mutants trained with cocaine. No significant differences were obtained between genotypes in inactive nose-pokes during operant conditioning maintained by cocaine nor during extinction (Supplementary Fig. S3B–C). Both genotypes trained with cocaine acquired the reinstatement criterion (double nose pokes in the active hole than the number of nose pokes during the 3 consecutive days when the mice acquired the extinction criteria) showing their capability to maintain this conditioning learning task. These data showed that *Plcb1*+/− resulted in a phenotype of decreased of cue-induced reinstatement with reduced cocaine-seeking (Fig. 2E).

### Brain transcriptomic analysis after the reinstatement of cocaine-seeking behavior

**Fig. 3:**
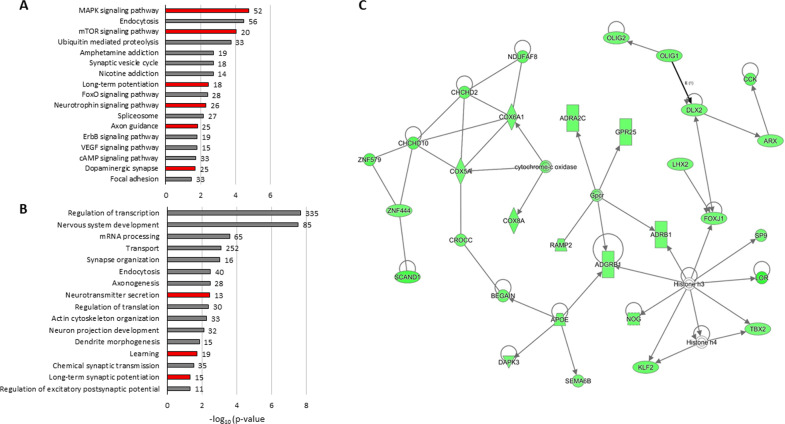
Gene expression changes in mPFC after cue-induced reinstatement of cocaine-seeking in *Plcb1*+/- vs wild-type (WT) mice. **A** Selection of over-represented KEGG pathways (Kyoto Encyclopedia of Genes and Genomes) and (**B**) GO (Gene Ontology) identified by DAVID software among the differentially expressed genes. The number of genes with altered expression included in each category is indicated on the right side of the bar. In red, relevant pathways for the addictive process. **C** Gene network involved in cellular development, cellular growth and proliferation, nervous system development and function (score = 62). The green nodes in the pathway indicate genes with downregulated expression in *Plcb1*+/− identified in RNAseq.

**Fig. 4:**
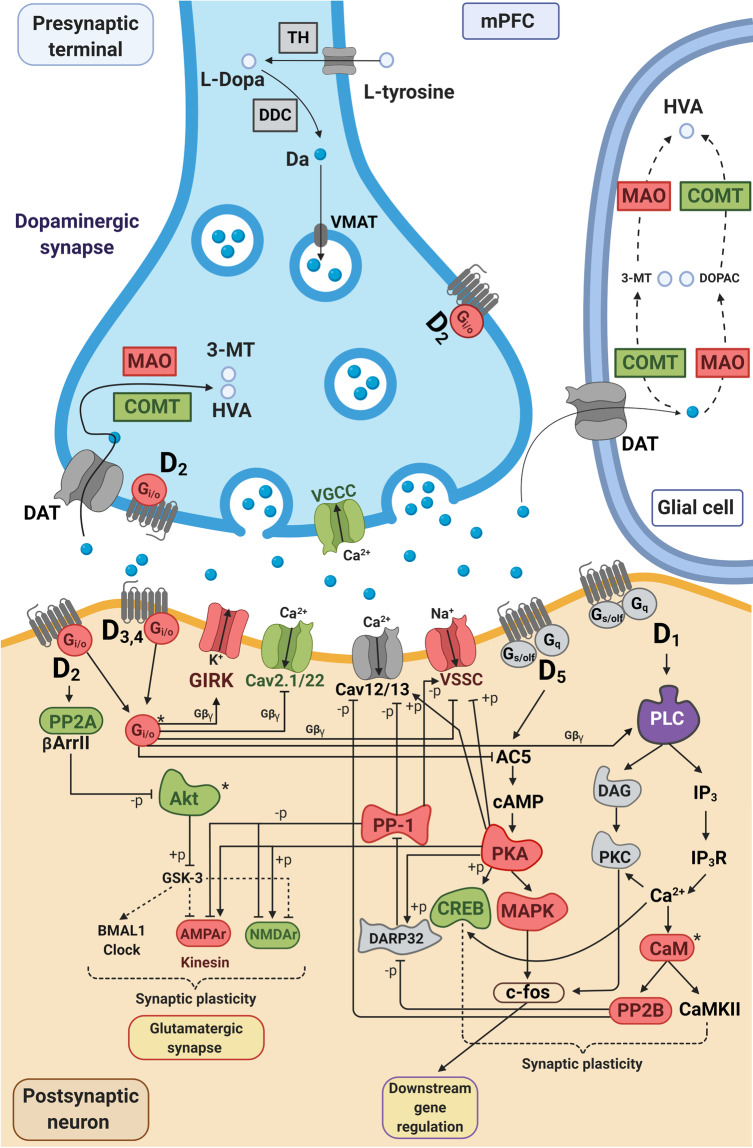
Alterations in expression in the dopaminergic synapse in mPFC of *Plcb1*+/− mice after cue-induced reinstatement of cocaine-seeking. Adapted from KEGG (Kyoto Encyclopedia of Genes and Genomes) pathways (mmu04728). Enriched pathway in differentially expressed genes in mPFC, 25 out of 131 genes in the pathway were differentially expressed (*P*_raw_ = 2e−03; *P*_ajd_ = 0.02). In red, upregulated genes and in green, downregulated genes in mPFC. *Protein complexes including upregulated and downregulated genes. Correspondence between genes and proteins can be found in Supplementary Table S6.

To further understand the role of PLCB1 in the molecular mechanisms involved in this cocaine relapse-related phenotype, we analyzed the transcriptomic profiles of mPFC and HPC immediately after the reinstatement of cocaine-seeking behavior in WT and *Plcb1*+/− mice trained with cocaine. We identified 2115 protein-coding genes differentially expressed (DEGs) in mPFC (1231 downregulated and 943 upregulated) and only 12 in HPC, when comparing *Plcb1*+/− and WT animals (Supplementary Tables S2–3). In accordance, principal component analysis (PCA) and heatmap plots revealed that individuals with the same genotype plotted together only in mPFC, but not in HPC (Supplementary Fig. S4). This suggested a more prominent role of mPFC, so further studies were carried out only with DEGs of this brain area. Analysis of functional group over-representation identified several processes previously related to cocaine addiction, including dopaminergic synapse, learning, long-term potentiation (LTP), neurotransmitter secretion, and axon guidance, as well as other relevant signaling pathways such as MAPK, mTOR, and neurotrophin (Fig. 3A, B and Supplementary Tables S3–5). We focused on the dopaminergic synapse pathway (enriched in the mPFCs DEGs; *P*_Adj_ < 0.05), in which phospholipase c (such as Plcb1) is directly participating in signal transmission (Fig. 4). Interestingly, many genes coding for proteins in this pathway are differentially expressed in *Plcb1*+/− mice after cue-induced reinstatement (Fig. 4 and Supplementary Table S6). Furthermore, we found that several TFBS were over‐represented in the DEGs of mPFC, including YY1, MYOD, NRF1, ERR1, FREAC2, NFY and E4F1 (complete list of TFBS in Supplementary Table S7). Then, we filtered the DEGs on mPFC based on fold-change (FC > | 1.2 | ) and obtained a list of 238 genes that we used for gene network construction. This analysis showed a highly scored network (score = 62, Fig. 3C) that includes 31 DEGs involved in “cellular development, cellular growth and proliferation, nervous system development and function”.

## Discussion

Here we investigated, for the first time, the contribution of the *PLCB1* gene to cocaine addictive properties using *Plcb1*+/− mice. We found that the heterozygous *Plcb1* genotype resulted in a phenotype of resistance to cue-induced reinstatement of cocaine-seeking behavior. Furthermore, we found relevant transcriptomic differences in *Plcb1*+/- mice compared to WT after cue-induced reinstatement of cocaine-seeking in mPFC, a brain area essential in relapse [35, 36]. This study supports a role for *PLCB1* in cocaine addiction, confirming previous findings in humans [6], and suggests it may be relevant in relapse to cocaine addiction.

The phenotype of *Plcb1*+/− mice was characterized at different levels to evaluate consummatory and general behavior. In general, mutant mice presented normal levels of body weight, food and water intake, locomotor activity, and motor coordination, demonstrating that this animal model is valid to study the effects of the heterozygous deletion of the *Plcb1* gene in other behavioral responses. *Plcb1*+/− showed increased anxiety in the elevated plus maze, reduced short-term memory in the novel object recognition paradigm, without any sign of depressive-like behavior in the anhedonia sucrose preference test. Thus, the single targeting of the *Plcb1* gene has an impact on selective emotional and cognitive responses.

The short-term memory impairment observed in the mutants did not affect the acquisition of operant associative conditioning nor the instrumental learning that drives the goal-directed action, as shown by similar levels of operant cocaine self-administration and extinction learning than WT. In agreement, both genotypes trained with cocaine accomplish cue-induced reinstatement criterion with high enhancement of responding during this test compared to extinction. Thus, different brain circuits are involved in each kind of learning, with perirhinal-hippocampal structures [37] participating in short-term memory in the novel object recognition paradigm and mPFC-dorsal striatum pathway in cue-associated seeking. Also differences in each paradigm such as the acute retrieval or repetitive exposure involved respectively in each task may have a crucial influence in cognition. Hence, cocaine-seeking can be multidimensional, involving different types of associative learning that together lead to an extensive repertoire of conditioned and instrumental responding.

The anxiogenic profile of *Plcb1*+/− is in accordance with the results previously reported in the *Plcb4*−/− mice, which were associated with alterations in the cholinergic activity of the medial septum [38]. However, the selective knock-down of *Plcb1* in the mPFC did not replicate this phenotype in a previous study [39], suggesting that other areas may be involved. The anxiogenic profile of *Plcb1*+/− mice had no effect on cocaine self-administration since the acquisition and extinction of this operant behavior was not modified in the mutants. Besides, mutants showed protection against cue-induced reinstatement of cocaine-seeking, instead of the expected cocaine-seeking promoted by an anxiogenic phenotype [40]. Therefore, the association of an anxiogenic profile with a phenotype resilient to cocaine-seeking in the *Plcb1*+/− mice suggests modifications in specific brain areas involved in cocaine relapse, such as the mPFC. Recently, a phenotype resilient to develop food addiction has been associated with increased strength of pyramidal glutamatergic synaptic transmission in the mPFC related to decreased compulsivity in the face of negative consequences [41] in mice with an anxiogenic profile [42]. Concerning cue-induced cocaine-seeking, the mPFC has a crucial role and the network of glutamatergic projections from the prelimbic mPFC to the dorsal striatum participates in this phenotype [43]. In our model, *Plcb1* haploinsufficiency is linked to reduced cue-induced cocaine-seeking possibly associated with modifications in this top-down corticolimbic brain network. Furthermore, these glutamatergic projections from mPFC to dorsal striatum receive mesocortical dopaminergic inputs from the VTA that could be crucially involved in the protective effects of *Plcb1*, since this gene plays an essential role in the dopaminergic signal transmission in the mPFC and many of the genes encoding for proteins in this pathway are differentially expressed in *Plcb1*+/− mice after cue-induced reinstatement (Fig. 4).

Transcriptomic analyses performed after the cue-induced reinstatement of cocaine-seeking behavior revealed some of the molecular underpinnings that underlie the cocaine relapse-related phenotype observed in the mutant mice. Interestingly, differences in gene expression between *Plcb1*+/− and WT were predominantly found in the mPFC, whereas almost no differences were observed in the HPC. This evidence highlights mPFC as a key region to explain these differences in cocaine-seeking reinstatement observed in *Plcb1*+/− mice. Consistently, in DEGs in mPFC, we found enrichment on pathways that are essential for the development of addiction, including the dopaminergic neurotransmission (Figs. 3, 4) [44]. *PLCB1* is part of the dopamine-DARPP-32 signaling pathway (Fig. 4), which plays a key role in cocaine reward [45, 46]. In a previous study, we also found this pathway enriched in DEGs in the frontal cortex and ventral striatum of mice that showed frustrated expected reward, produced by the cue in the absence of expected reward after a high level of effort in a progressive ratio schedule of reinforcement with palatable food [47]. Notably, *Plcb1* was upregulated in the frontal cortex of those frustrated mice with increased responses to obtain the reward (palatable food). These data are in line with our findings in which decreased expression of *Plcb1* (*Plcb1*+/− mice) results in decreased cue-induced responses to obtain cocaine after extinction. All these results support the participation of *Plcb1* in cue-induced cocaine-seeking revealed in the present study and the involvement of mPFC.

Our transcriptomic analyses also found enrichment in genes related to learning and memory processes, such as LTP in the mPFC. The transition from drug use to drug addiction is a maladaptive process that directly affects learning and memory [48, 49]. LTP produces long-lasting activity-dependent synaptic modifications that underlie memory, and drugs of abuse alter this mechanism in several brain regions such as mPFC [50, 51], mesocorticolimbic system [52], VTA [53] and HPC [54, 55], among others. Importantly, genes related to the glutamatergic system such as *Gria2-4* (glutamate ionotropic receptor AMPA2-4 (alpha 2-4)) and *Grik2* (ionotropic glutamate receptor kainate 2 (beta 2)) were found to be upregulated in *Plcb1*+/− mice. Meanwhile, *Grin1, 2c, and 2d* (glutamate ionotropic receptor NMDA1 (zeta 1, epsilon3 and epsilon4)) were downregulated, suggesting an increase in AMPAR/NMDAR ratio in cocaine-experienced and abstinent *Plcb1*+/− mice as compared to WT, one of the key indicators of LTP induction and increased synaptic strength [56]. Furthermore, we found enrichment in other neuroplasticity (synapse organization, neuron projection development, and axon guidance) [14, 57–59] and signaling pathways (MAPK, mTOR, and neurotrophin) [60, 61] related to addiction. The assessment of gene expression in the mPFC of the *Plcb1*+/− mice highlighted alterations in relevant pathways for the addictive process, which could contribute to the results observed in the cue-induced reinstatement.

Nowadays, there are few effective pharmacological treatments available for cocaine use disorders, and frequently, psychosocial interventions in combination with pharmacotherapy are needed [62]. Studies performed in mice have pinpointed multiple potential therapeutic approaches for the reinstatement of cocaine-seeking behavior, targeting the reward circuit [63]. In humans, treatment with bupropion, topiramate, or disulfiram has been widely used. Bupropion, a non-tricyclic antidepressant that inhibits dopamine and norepinephrine reuptake, is effective in reducing craving [64]. Topiramate, a GABA/glutamatergic medication, has also been used to treat cocaine use disorder as it reduces the activity of the mesocorticolimbic dopaminergic system [62]. A completely different strategy is the use of disulfiram which potentially inhibits the oxidoreductase dopamine β‐hydroxylase (DβH, encoded by the *DBH* gene), which converts dopamine to norepinephrine [65]. Significantly, a genetic variation in the *SLC6A3* gene (encoding DAT) has been associated with disulfiram treatment for cocaine addiction, with patients with higher DAT levels having better treatment outcomes than those with lower DAT levels [65]. Thus, further studies and new therapeutic targets are needed to obtain effective treatment for cocaine addiction. The results obtained in the present study underscore the relevance of the *Plcb1* gene in the cue-induced reinstatement of cocaine-seeking after extinction. Together with previous findings in humans [6, 7] and mice [8], *PLCB1* merits to be further evaluated as a promising novel therapeutic target for preventing relapse and treating cocaine addiction.

The experimental approach used in our study, the *Plcb1*+/− mouse model, allowed us to reproduce better the molecular context observed in humans in comparison to the use of a complete KO mouse [66], as these animals preserved, at least, half of the expression of *Plcb1*. However, the haploinsufficiency of *Plcb1* during neurodevelopment in these animals could produce alterations that may contribute to the phenotype observed in the present study. Nevertheless, this may also be similar in humans with genetic risk variants that decrease *PLCB1* expression. Therefore, this approach is appropriate to study a specific genetic alteration that confers susceptibility to drug addiction and to delineate the precise contribution of *PLCB1*.

To sum up, we studied, for the first time, the contribution of the *PLCB1* gene to cocaine addictive properties using *Plcb1*+/− mice. Previous studies have revealed an upregulation of *Plcb1* in brain areas related to the reward circuit after cocaine exposure in animals [8] and in human cocaine abusers [6]. These changes, together with our results, suggest that cocaine increases the expression of *Plcb1*, and this mechanism plays an essential role in cocaine addiction, as revealed now by the resistance to cue-induced reinstatement of cocaine-seeking behavior exhibited by *Plcb1*+/− mutant mice. These results highlight the importance of the *Plcb1* gene in the development of cocaine addiction and relapse and pinpoint PLCB1 as a promising therapeutic target for cocaine addiction and perhaps other types of addiction.

## Supplementary information

Supplementary information

Supplementary tables
