# Supplementary material for: Reduced cue-induced reinstatement of cocaine-seeking behavior in Plcb1 +/− mice
Source: Transl Psychiatry. 2021 Oct 11;11:521. doi: 10.1038/s41398-021-01396-6 (PMC8505421; doi:10.1038/s41398-021-01396-6)
Supplement: Supplementary file 1 — Supplementary information [file 41398_2021_1396_MOESM1_ESM.docx]

**SUPPLEMENTARY INFORMATION**

**MATERIALS AND METHODS**

**Genotyping of transgenic mice**

*Plcb1+/-* mice in a C57BL/6J background were kindly provided by H.-S. Shin ^1^. Very briefly, the knockout animals were generated by the replacement of exons that encode aminoacid residues 50–82 by a Neomycin cassette that was used both for gene disruption and for positive selection.

To genotype the mice, genomic DNA from ear punches or tail tips was extracted as previously described ^2^. Genotyping was carried out by PCR on extracted genomic DNA using three specific PCR primers: a sense primer, 5′-GTTAAGTCCTCAGGCAAACACC-3’, and two antisense primers, 5′-ACCTTGGGAGCTTTGGCGTG3’ and 5′-CTGACTAGGGGAGGAGTAGAAG-3’, that allowed us to amplify a 180bp Plcb1+/+ band and a 290bp Plcb1-/- band ^3^. Fragments were separated by 2.5 % agarose gel electrophoresis. Known WT and *Plcb1+/-* controls were always used to validate genotyping results. The genotype of all animals used was confirmed after the cue-induced reinstatement.

**Drugs**

Ketamine hydrochloride (Imalgène; Merial Laboratorios S.A., Barcelona, Spain), medetomidine hydrochloride (Domtor; Esteve, Barcelona, Spain), atipamezole hydrochloride (Revertor; Virbac, Barcelona, Spain), meloxicam (Metacam; Boehringer Ingelheim, Rhein, Germany) and gentamicine (Genta-Gobens; Laboratorios Normon, S.A., Madrid, Spain) were dissolved in sterile 0.9% physiological saline and administered in an injection volume of 10 ml/kg of body weight. For operant conditioning maintained by cocaine, cocaine hydrochloride (obtained from The Spanish Agency of Drugs, Madrid, Spain) was dissolved in a saline solution (0.9% NaCl w/v).

**Behavioral tests for phenotype characterization**

The details of the behavioral tests used for phenotype characterization are described below.

*Short-term memory.* To measure short-term memory, the novel object recognition (NOR) test was used. This test was performed in a V-maze apparatus (40 cm per side, Panlab, Spain) as previously described by us and others ^4,5^. On day 1 mice were habituated for 9 minutes in the V-maze. On day 2 mice were put back into the V-maze for 9 minutes; two identical objects were presented, and the time the mice spent exploring each object was recorded. After a retention phase of 3 hours, the mice were placed for another 9 minutes into the V-maze; in each paradigm one of the familiar objects was replaced with a novel object and the total time spent exploring each object (novel and familiar) was registered. Object exploration was defined as the orientation of the nose to the object at a distance of less than 2 cm. A discrimination index was calculated as the difference of the time spent exploring the novel and the time spent exploring the familiar object divided by the total time exploring both objects. A higher discrimination index is considered to reflect greater memory retention for the familiar object.

*Locomotor activity*. Locomotor activity was assessed in locomotor activity boxes (9×20×11 cm; Imetronic, Passac, France), equipped with 2 rows of photocell detectors, and placed in a low-luminosity environment (20–25 lux), as previously described ^6^. The locomotor activity was recorded for 2 hours and 2 variables were measured: horizontal activity as horizontal displacement and vertical activity as vertical exploration/elevation.

*Elevated plus maze test*. This test was performed as previously reported ^5^. The test measures the conflict between the natural tendency of mice to avoid an illuminated and elevated surface, and the natural tendency to explore new environments. It consisted of a black plastic cross with arms 40 cm long and 6 cm wide placed 50 cm above the floor. Two opposite arms were surrounded by walls (15 cm high, closed arms, 10 lux), while the two other arms were devoid of such walls (open arms, 200 lux). A central platform connected the four arms. At the start of the session, the mouse was placed at the end of a closed arm facing the wall. During the 5-minutes trial, the number of entries and the time spent in each arm were recorded. Anxiety was assessed as both the time spent avoiding the open arms and the number of entries into them.

*Motor coordination*. Rota rod apparatus was used to assess motor coordination. Specifically, this test measures the ability of the mouse to remain on a rotating rod in which the speed of rotation is gradually increased and evaluate general motor coordination. The device is a round drum (5 cm diameter) suspended between 2 plexiglass walls. The drum is suspended at 24 cm from a soft mat covered tabletop. The speed of rotation is controlled by an electric engine with a digital revolution per min (rpm) display. The test consisted in five trial sessions. Each trial started with the mouse being placed on the rotating rod at 4.0 rpm, then every 3 s the speed increased by 1.0 rpm until 20.0 rpm. The trial terminated when the mouse fell from the rod or after 90 s, whichever occurred first. There was a 10 s interval between trials. The average maximum rota rod speed and time to fall was calculated for each mouse ^6^.

*Experimental procedure for food and drink monitoring.*

*Habituation.* Six animals per genotype were taken from their home cages and placed in experimental chambers named PHECOMP boxes (Panlab and Harvard Apparatus) equipped with a food, drink and motor activity monitoring system (<https://www.panlab.com/en/products/phecomp-system-panlab>) for two weeks of habituation. Animals were monitored in these boxes 24 h a day during the whole experimental period.

*Sucrose preference test.* After a period of habituation, sucrose preference test was performed on the third week of PheComp cage housing during one week, as previously described ^4,5^. Two bottles of water, one with 2% sucrose and the other without, were placed in the cage. The position of bottles was exchanged everyday, and the consumption from each bottle measured after a 24h interval. The preference for sucrose was calculated as the relative amount of water with sucrose versus total liquid (water with and without sucrose) consumed by the mice ^4^.

**Operant conditioning maintained by cocaine**

*Operant self-administration apparatus*. The self-administration experiments were conducted in mouse operant chambers (Model ENV-307A-CT; Med Associates Inc., Georgia, VT, USA) equipped with two holes, one randomly selected as the active hole and the other as the inactive hole. The chambers were made of aluminum and acrylic, with grid floors and were housed in sound- and light-attenuated boxes equipped with fans to provide ventilation and white noise. Pump noise and stimuli lights (cues), one located inside the active hole and the other above it, were paired contingently with the delivery of the reinforcer. Cocaine was infused via a syringe that was mounted on a microinfusion pump (PHM-100A; Med Associates) and connected, via Tygon tubing (0.96 mm o.d., Portex Fine Bore Polythene Tubing, Portex Ltd, Kent, UK) to a single-channel liquid swivel (375/25, Instech Laboratories, Plymouth Meeting, PA, USA) and to the mouse intravenous catheter. The swivel was mounted on a counter-balanced arm above the operant chamber.

*Operant conditioning maintained by cocaine*. Cocaine self-administration experiments were performed as previously described ^7,8^. Mice were deeply anaesthetized by intraperitoneal injection of a mixture of ketamine (75 mg/kg) and medetomidine (1 mg/kg). After surgery, anesthesia was reversed by subcutaneous injection of the synthetic α2 adrenergic receptor antagonist, atipamezole (2.5 mg/kg) indicated for the reversal of the sedative and analgesic effects of medetomidine (α2 adrenergic receptor agonist). In addition, mice received an intraperitoneal injection of gentamicine (1 mg/kg) along with subcutaneous administration of the analgesic meloxicam (2 mg/kg). Each 2-h daily self-administration session started with a priming injection of the drug. Cocaine was infused in 23.5 μl over 2 s (0.5 mg/kg per injection, intravenously). Cue light, located above the active hole, was paired with the delivery of the reinforcer. Mice (WT n = 36; *Plcb1+/-* n = 26) were trained under a fixed ratio 1 schedule of reinforcement (FR1; one nose-poke lead to the delivery of one dose of cocaine) over 5 consecutive daily sessions and under a fixed ratio 3 (FR3) over 5 consecutive daily sessions. Control mice trained with saline were included for both genotypes (WT n = 6; *Plcb1+/-* n = 6). The timeout period after infusion delivery was 10 s. Responses on the inactive hole and all responses elicited during the 10-s timeout period were also recorded. Responses during the 10-s timeout period were considered a measure of impulsivity reflecting the inability to stop motor behavior once it is initiated. The criteria for self-administration behavior were achieved when all of the following conditions were met: (1) mice maintained stable, responding with 20% deviation from the mean of the total number of reinforcers earned in three consecutive sessions (80% of stability); (2) at least 75% of mice responding on the active hole; and (3) a minimum of 10 reinforcers per session. After the 10 FR sessions, animals were tested in a progressive ratio (PR) schedule of 4 h where the response requirement to earn the cocaine escalated according to the following series: 1–2–3–5–12–18–27–40–60–90–135–200–300–450–675–1000. On day 12, mice were moved from the cocaine self-administration/training phase to the extinction phase. The experimental conditions during the extinction phase were similar to the cocaine self-administration sessions except that cocaine was not available, and cue-light was not presented after nose poking in the active hole. Mice were given 2-h daily sessions until they achieved the extinction criterion with a maximum of 26 sessions. The criterion for extinction was achieved when, during 3 consecutive sessions, mice completed a mean number of nose pokes in the active hole consisting of 30% of the mean responses obtained during the 3 days to achieve the acquisition criteria for cocaine self-administration training. On day 27, only mice that accomplish extinction criterion were tested in the cue-induced reinstatement during a 2-h session. The presentation of conditioned environmental cues was performed to evaluate the reinstatement of cocaine-seeking behavior. The test for cue-induced reinstatement was conducted under the same conditions used in the training phase except that cocaine was not available. The reinstatement criterion was achieved when nose pokes in the active hole were double the number of nose pokes in the active hole during the 3 consecutive days when the mice acquired the extinction criteria. The catheter was flushed daily with heparinized saline (30 USP units/ml). The patency of intravenous catheters was evaluated after the last cocaine self-administration session and whenever the behavior appeared to deviate dramatically from that observed previously by infusion of thiopental through the catheter. If prominent signs of anesthesia were not apparent within 3s of the infusion, the mouse was removed from the experiment. The success rate for maintaining patency of the catheter (mean duration of 11 days) until the end of the cocaine self-administration training was 90%.

**RNA extraction**

Total RNA of 12 WT and 11 *Plcb1+/-* mice from mPFC and HPC were extracted. Samples were homogenized using the TissueRuptor system (Qiagen, Düsseldorf, Germany), and total RNA was isolated using the RNeasy Lipid Tissue Mini Kit (Qiagen) according to the manufacturer's protocol. RNA concentration was determined using the NanoDrop ND‐1000 spectrophotometer (NanoDrop Technologies, Wilmington, DE, USA), and integrity was evaluated using the Bioanalyzer2100 platform (Agilent Technologies, Santa Clara, CA, USA). RNA samples were grouped in 4 pools consisting of 3 mice per pool for each experimental group, except for 1 group in the *Plcb1+/-* mice were only 2 animals were pooled. The pools were organized to homogenize the average number of nose pokes in the different pools. The pooled individuals were the same for both mPFC and HPC.

**Library preparation and RNA sequencing**

RNA sequencing (RNAseq) was performed by the Centre de Regulació Genòmica (CRG, Barcelona, Spain). Libraries were prepared using the TruSeq Stranded mRNA Sample Prep Kit v2 (Illumina, San Diego, CA, USA) according to the manufacturer's protocol. Briefly, 250 ng of total RNA were used for poly(A)-mRNA selection using streptavidin-coated magnetic beads and were subsequently fragmented to approximately 300bp. cDNA was synthesized using reverse transcriptase (SuperScript II, Invitrogen, Carlsbad, CA, USA) and random primers. The second strand of the cDNA incorporated dUTP instead of dTTP. Double-stranded DNA was used for library preparation. dsDNA was subjected to A-tailing and ligation of the barcoded Truseq adapters. Library amplification was performed by PCR using the primer cocktail supplied in the kit. All purification steps were performed using AMPure XP beads. Final libraries were analyzed using a LabChip instrument to estimate quantity and check size distribution, and were then quantified by qPCR using the KAPA Library Quantification Kit (Roche, Basilea, Swissland) prior to amplification with Illumina’s cBot. Libraries were sequenced on the Illumina’s HiSeq 3000 system, considering 75bp paired-ends (PE) reads for both mPFC and HPC. The analysis of RNAseq was carried out by the Bioinformatics service of CRG. Briefly, FastQC v0.11.5 ^9^ was used to inspect the reads quality and CutAdapt 1.7.1 ^10^ was used to clean the data of adapters and low quality reads. Then, reads were mapped against the *Mus musculus* genome of reference (GRCm38/mm10) with STAR 2.5.3a^11^. Finally, the differential expression analysis was done using DESeq2 ^12^ to compare individuals with different genotypes (WT vs *Plcb1+/-*). Corrections for multiple testing were applied by adjusting the p-values with a 5% False Discovery Rate (FDR).

RNAseq data of mPFC and HPC were explored on a principal component analysis (PCA) plot using the "plotPCA" method from the DESeq2 package ^13^ and log2 gene expression data. The PCAs were performed with the 500 genes showing the highest variance among the samples to calculate the distance among them. The heatmaps were performed using the “heatmap” function on R and the hierarchical clustering considered the euclidean distance between the samples considering all the genes or only those with corrected p-value < 1e-05.

**REFERENCES**

1 Kim D et al. Phospholipase C isozymes selectively couple to specific neurotransmitter receptors. *Nature* 1997; **389**: 290–293.

2 Gómez-Grau M et al. New murine Niemann-Pick type C models bearing a pseudoexon-generating mutation recapitulate the main neurobehavioural and molecular features of the disease. *Sci Rep* 2017; **7**. doi:10.1038/srep41931.

3 Filis P et al. Phospholipase C-β1 signaling affects reproductive behavior, ovulation, and implantation. *Endocrinology* 2009; **150**: 3259–3266.

4 Bura SA, Burokas A, Martin-Garcia E, Maldonado R. Effects of chronic nicotine on food intake and anxiety-like behaviour in CB(1) knockout mice. *Eur Neuropsychopharmacol* 2010; **20**: 369–378.

5 Planaguma J et al. Human N-methyl D-aspartate receptor antibodies alter memory and behaviour in mice. *Brain* 2015; **138**: 94–109.

6 Martin-Garcia E et al. Intrathecal injection of P/Q type voltage-gated calcium channel antibodies from paraneoplastic cerebellar degeneration cause ataxia in mice. *JNeuroimmunol* 2013; **261**: 53–59.

7 Martín-García E et al. Differential Control of Cocaine Self-Administration by GABAergic and Glutamatergic CB1 Cannabinoid Receptors. *Neuropsychopharmacology* 2015; : 1–14.

8 Gutierrez-Cuesta J et al. Effects of genetic deletion of endogenous opioid system components on the reinstatement of cocaine-seeking behavior in mice. *Neuropsychopharmacology* 2014; **39**: 2974–2988.

9 Andrews S. FastQC: A quality control tool for high throughput sequence data. 2010.

10 Martin M. Cutadapt removes adapter sequences from high-throughput sequencing reads. *EMBnet.journal* 2011; **17**: 10.

11 Dobin A et al. STAR: Ultrafast universal RNA-seq aligner. *Bioinformatics* 2013; **29**: 15–21.

12 Love MI, Huber W, Anders S. Moderated estimation of fold change and dispersion for RNA-seq data with DESeq2. *Genome Biol* 2014; **15**: 550.

13 Love MI, Huber W, Anders S. Moderated estimation of fold change and dispersion for RNA-seq data with DESeq2. *Genome Biol* 2014; **15**: 550.

**SUPPLEMENTARY FIGURES**

**
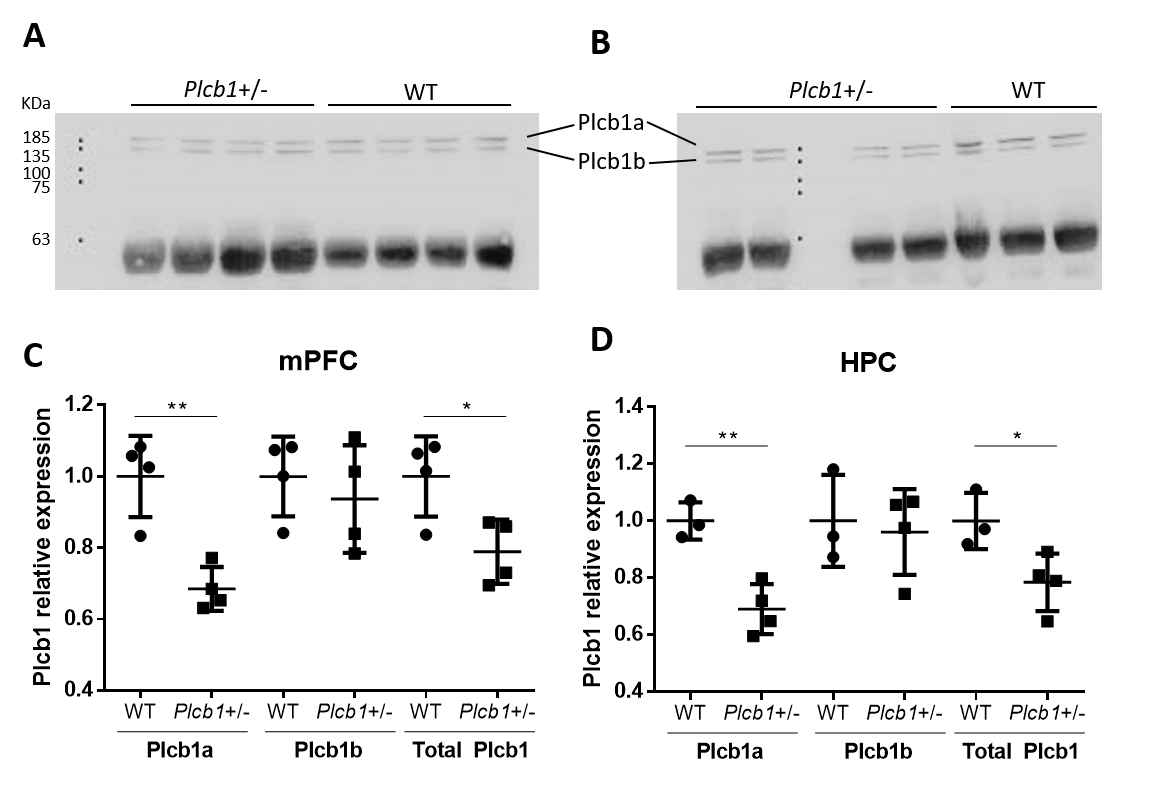
**

**Supplementary Figure S1. Western blot analysis of Plcb1 protein expression in naive mice.** Results of western blot against Plcb1 protein and quantification on (A,C) medial prefrontal cortex (mPFC) and (B,D) hippocampus (HPC) comparing *Plcb1+/-* and WT naive mice not exposed to cocaine. Tubulin was used as the loading control. **P*<0.05; ***P*<0.01.


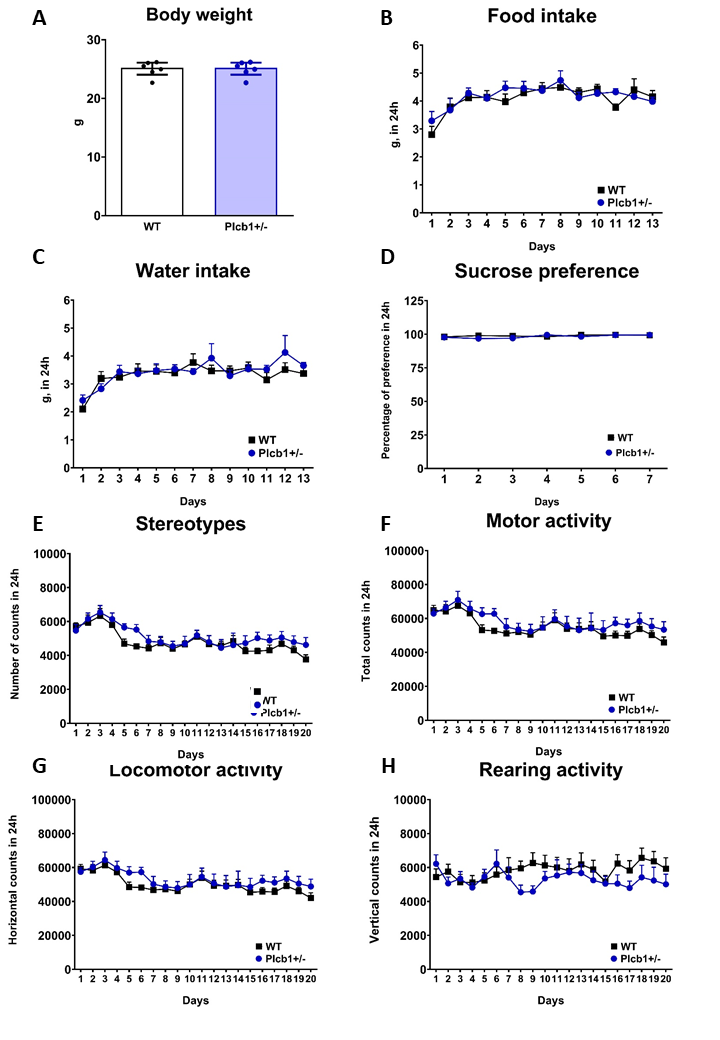


**Supplementary Figure S2. Experimental procedure for food and drink monitoring in PheComp boxes.** (A) Average of body weight measured during the whole experimental sequence. (B) Food and (C) water intake measured daily in PheComp boxes. (D) Percentage of sucrose preference in a two-bottle choice procedure. (E) Number of counts of stereotyped behavior measured daily in the PheComp boxes. (F) Motor (G) locomotor and (H) rearing activity registered every 24h in the PheComp boxes. All data are expressed in median and interquartile range and individual data are shown in body weight and mean±SEM in the rest of variables (n=6 per genotype).


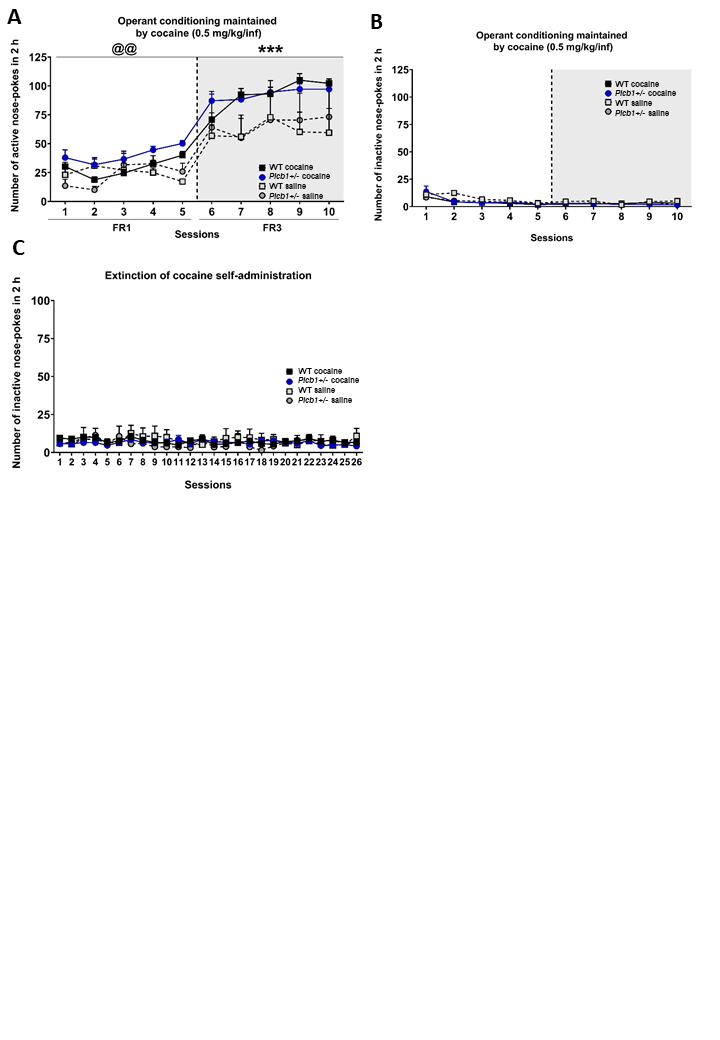


**Supplementary Figure S3. Operant conditioning maintained by cocaine in *Plcb1+/-*, wild-type (WT) and corresponding control saline mice.** (A) The levels of active nose poking during FR1 similarly increased in both genotypes trained with cocaine (repeated measures ANOVA, interaction between drug x sessions, @@*P*<0.01) and remained higher in mice trained with cocaine than with saline in FR3 (repeated measures ANOVA, main effect of drug, ***P*<0.01). Inactive nose-pokes during the (B) operant conditioning maintained by cocaine and during (C) extinction of operant behavior. All data are expressed in mean±SEM (WT cocaine n=36; *Plcb1+/-* cocaine n=26; saline n=6 per genotype).


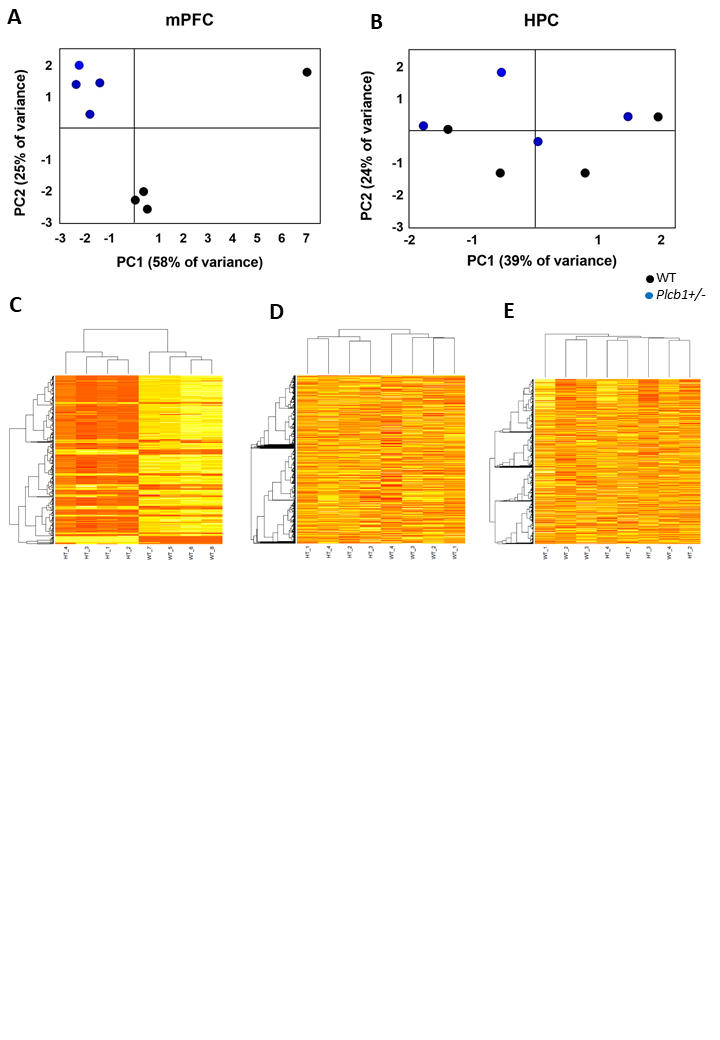


**Supplementary Figure S4. Results of RNAseq from comparing *Plcb1+/-* mice and wild-type (WT) after cue-induced reinstatement of cocaine seeking behaviour.** (A) Principal component analysis (PCA) of medial prefrontal cortex (mPFC) and (B) hippocampus (HPC). (C) Heatmap and hierarchical clustering from top differentially expressed genes in mPFC (genes with corrected *P* < 1e-05) and (D) all genes from mPFC and (E) HPC.
