# Supplementary material for: Reduced cue-induced reinstatement of cocaine-seeking behavior in Plcb1 +/− mice
Source: Transl Psychiatry. 2021 Oct 11;11:521. doi: 10.1038/s41398-021-01396-6 (PMC8505421; doi:10.1038/s41398-021-01396-6)
Supplement: Supplementary file 2 — Supplementary tables [file 41398_2021_1396_MOESM2_ESM.pdf]

Supplementary Table S1. Operant responding maintained by cocaine during FR1 and FR3 (infusions and active nose-pokes) and extinction (active nose-pokes).

	Three-way ANOVA			
	FR1 Infusions		FR3 Infusions	
	F -value	*P* -value	*F* -value	*P* -value
Genotype	$F_{(1,70)}$ = 0.40	*n.s.*	$F_{(1,70)}$ = 0.02	*n.s.*
Drug	$F_{(1,70)}$ = 5.91	P < 0.05	$F_{(1,70)}$ = 12.78	P < 0.001
Sessions	$F_{(4,280)}$ = 9.15	P < 0.001	$F_{(4,280)}$ =3.34	P < 0.05
Genotype × Drug	$F_{(1,70)}$ = 2.41	*n.s.*	$F_{(1,70)}$ = 0.02	*n.s.*
Genotype × Sessions	$F_{(4,280)}$ = 3.80	P < 0.01	$F_{(4,280)}$ = 0.30	*n.s.*
Drug × Sessions	$F_{(4,280)}$ = 5.25	P < 0.01	$F_{(4,280)}$ = 0.23	*n.s.*
Genotype × Drug × Sessions	$F_{(4,280)}$ = 2.81	P < 0.05	$F_{(4,280)}$ = 0.81	*n.s.*
	FR1 Active nose-pokes		FR3 Active-nose-pokes	
	F -value	*P* -value	*F* -value	*P* -value
Genotype	$F_{(1,70)}$ = 0.86	*n.s.*	$F_{(1,70)}$ = 0.10	*n.s.*
Drug	$F_{(1,70)}$ = 4.96	P < 0.05	$F_{(1,70)}$ = 9.77	P < 0.01
Sessions	$F_{(4,280)}$ = 3.99	P < 0.01	$F_{(4,280)}$ =3.59	P < 0.01
Genotype × Drug	$F_{(1,70)}$ = 1.70	*n.s.*	$F_{(1,70)}$ = 0.08	*n.s.*
Genotype × Sessions	$F_{(4,280)}$ = 1.92	*n.s.*	$F_{(4,280)}$ = 0.66	*n.s.*
Drug × Sessions	$F_{(4,280)}$ = 3.62	P < 0.01	$F_{(4,280)}$ = 1.36	*n.s.*
Genotype × Drug × Sessions	$F_{(4,280)}$ = 1.96	*n.s.*	$F_{(4,280)}$ = 0.85	*n.s.*
	Extinction			
	F -value	*P* -value		
Genotype	$F_{(1,55)}$ = 2.94	*n.s.*		
Drug	$F_{(1,55)}$ = 8.28	P < 0.01		
Sessions	$F_{(25,1375)}$ =41.65	P < 0.001		
Genotype × Drug	$F_{(1,55)}$ = 0.55	*n.s.*		
Genotype × Sessions	$F_{(25,1375)}$ = 1.18	P < 0.001		
Drug × Sessions	$F_{(25,1375)}$ = 4.09	*n.s.*		
Genotype × Drug × Sessions	$F_{(25,1375)}$ = 0.59	*n.s.*		

Three-way ANOVA with genotype and drug as between-subjects factor and repeated measures in the factor sessions. See materials and methods for details. *n.s.:* non significant

Supplementary Table S2. Gene expression changes in mPFC (FDR 5%) in Plcb1+/- mice compared to WT after cocaine seeking reinstatement

ids	baseMean	log2FoldChange	lfcSE	stat	pvalue	padj	Gene Name	Gene type
ENSMUSG00000095098.1	2428.060	-0.937	0.055	-16.952	1.86E-64	2.63E-60	Ccdc85b	protein_coding
ENSMUSG00000071076.6	2879.048	-0.949	0.060	-15.696	1.60E-55	1.13E-51	Jund	protein_coding
ENSMUSG00000020496.9	12161.061	-0.348	0.025	-13.977	2.16E-44	1.01E-40	Rnf187	protein_coding
ENSMUSG00000070493.3	4689.425	-0.441	0.035	-12.762	2.66E-37	9.37E-34	Chchd2	protein_coding
ENSMUSG00000060032.6	950.218	-0.648	0.055	-11.849	2.17E-32	6.11E-29	H2afj	protein_coding
ENSMUSG00000044533.15	8067.372	-0.366	0.032	-11.564	6.26E-31	1.47E-27	Rps2	protein_coding
ENSMUSG00000054871.5	1236.200	-0.652	0.057	-11.506	1.23E-30	2.47E-27	Tmem158	protein_coding
ENSMUSG00000070462.4	1279.727	-0.534	0.047	-11.467	1.92E-30	3.39E-27	Mesdc1	protein_coding
ENSMUSG00000040860.16	7894.329	-0.375	0.033	-11.245	2.44E-29	3.82E-26	Crocc	protein_coding
ENSMUSG00000047085.14	6553.856	-0.469	0.042	-11.236	2.72E-29	3.84E-26	Lrrc4b	protein_coding
ENSMUSG00000039278.10	10220.556	-0.820	0.073	-11.225	3.06E-29	3.92E-26	Pcsk1n	protein_coding
ENSMUSG00000078794.4	4932.143	-0.451	0.040	-11.179	5.19E-29	6.09E-26	Dact3	protein_coding
ENSMUSG00000029581.14	8409.185	-0.339	0.031	-11.055	2.08E-28	2.25E-25	Fscn1	protein_coding
ENSMUSG00000002393.14	903.882	-0.717	0.065	-10.993	4.14E-28	4.02E-25	Nr2f6	protein_coding
ENSMUSG00000034685.4	3656.144	-0.459	0.042	-10.990	4.28E-28	4.02E-25	Fam171a2	protein_coding
ENSMUSG00000049932.3	977.087	-0.655	0.060	-10.936	7.72E-28	6.80E-25	H2afx	protein_coding
ENSMUSG00000000740.12	4710.356	-0.556	0.051	-10.823	2.69E-27	2.23E-24	Rpl13	protein_coding
ENSMUSG00000043165.7	161.243	-0.817	0.077	-10.627	2.22E-26	1.74E-23	Lor	protein_coding
ENSMUSG00000034686.7	865.971	-0.716	0.068	-10.476	1.11E-25	8.26E-23	Prr7	protein_coding
ENSMUSG00000049422.7	3632.917	-0.545	0.052	-10.421	2.00E-25	1.41E-22	Chchd10	protein_coding
ENSMUSG00000054716.4	810.956	-0.763	0.074	-10.243	1.27E-24	8.55E-22	Zfp771	protein_coding
ENSMUSG00000039860.19	3319.619	-0.349	0.034	-10.137	3.79E-24	2.43E-21	Srrm3	protein_coding
ENSMUSG00000043445.6	1473.625	-0.597	0.059	-10.056	8.66E-24	5.31E-21	Pgp	protein_coding
ENSMUSG00000022577.16	7170.974	-0.418	0.043	-9.842	7.43E-23	4.37E-20	Ly6h	protein_coding
ENSMUSG00000025226.11	640.336	-0.617	0.063	-9.833	8.08E-23	4.56E-20	Fbxl15	protein_coding
ENSMUSG00000013858.14	4459.271	-0.349	0.036	-9.648	5.00E-22	2.71E-19	Tmem259	protein_coding
ENSMUSG00000035885.4	9047.900	-0.409	0.042	-9.622	6.48E-22	3.38E-19	Cox8a	protein_coding
ENSMUSG00000040794.5	2722.233	-0.701	0.074	-9.506	1.97E-21	9.62E-19	C1qtnf4	protein_coding
ENSMUSG00000075227.6	616.338	-0.552	0.058	-9.506	1.98E-21	9.62E-19	Znhit2	protein_coding
ENSMUSG00000064264.14	891.665	-0.555	0.059	-9.430	4.09E-21	1.92E-18	Zfp428	protein_coding

ENSMUSG00000040867.12	5995.125	-0.398	0.042	-9.415	4.73E-21	2.15E-18	Begain	protein_coding
ENSMUSG00000051550.10	1024.796	-0.576	0.062	-9.368	7.36E-21	3.24E-18	Zfp579	protein_coding
ENSMUSG00000002985.16	41842.922	-0.360	0.038	-9.362	7.80E-21	3.33E-18	Apoe	protein_coding
ENSMUSG00000035342.13	2569.807	-0.365	0.039	-9.294	1.48E-20	5.98E-18	Lzts2	protein_coding
ENSMUSG00000007944.8	2889.222	-0.418	0.045	-9.294	1.49E-20	5.98E-18	Ttc9b	protein_coding
ENSMUSG00000070858.12	358.331	-0.665	0.073	-9.095	9.45E-20	3.66E-17	Gm1673	protein_coding
ENSMUSG00000030600.15	2588.959	-0.433	0.048	-9.093	9.60E-20	3.66E-17	Lrfn1	protein_coding
ENSMUSG00000044030.4	1827.063	-0.360	0.040	-9.035	1.65E-19	6.11E-17	Irf2bp1	protein_coding
ENSMUSG00000071341.4	1550.943	-0.693	0.077	-9.002	2.22E-19	8.03E-17	Egr4	protein_coding
ENSMUSG00000090071.4	8832.339	-0.379	0.042	-8.989	2.49E-19	8.77E-17	Cdk5r2	protein_coding
ENSMUSG00000050896.12	1472.949	-0.506	0.057	-8.896	5.77E-19	1.98E-16	Rtn4rl2	protein_coding
ENSMUSG00000046229.9	432.648	-0.664	0.075	-8.832	1.03E-18	3.45E-16	Scand1	protein_coding
ENSMUSG00000032174.5	12025.867	-0.339	0.038	-8.813	1.22E-18	4.01E-16	Icam5	protein_coding
ENSMUSG00000027447.6	26537.549	-0.248	0.028	-8.792	1.46E-18	4.69E-16	Cst3	protein_coding
ENSMUSG00000029419.8	6653.533	-0.321	0.037	-8.762	1.92E-18	6.02E-16	Gm996	protein_coding
ENSMUSG00000046215.3	1286.568	-0.536	0.061	-8.717	2.86E-18	8.75E-16	Rprml	protein_coding
ENSMUSG00000089762.3	692.006	-0.562	0.065	-8.705	3.17E-18	9.51E-16	Ier5l	protein_coding
ENSMUSG00000070003.7	3940.817	-0.464	0.053	-8.679	4.01E-18	1.18E-15	Ssbp4	protein_coding
ENSMUSG00000035011.15	6673.966	-0.360	0.042	-8.566	1.07E-17	3.07E-15	Zbtb7a	protein_coding
ENSMUSG00000037499.9	1132.999	-0.391	0.046	-8.558	1.15E-17	3.25E-15	Nenf	protein_coding
ENSMUSG00000020308.6	1119.688	-0.545	0.064	-8.539	1.35E-17	3.73E-15	Tpgs1	protein_coding
ENSMUSG00000049036.7	489.513	-0.564	0.066	-8.511	1.72E-17	4.67E-15	Tmem121	protein_coding
ENSMUSG00000042388.14	14098.397	-0.368	0.043	-8.506	1.79E-17	4.77E-15	Dlgap3	protein_coding
ENSMUSG00000105867.2	1502.706	-0.393	0.046	-8.493	2.02E-17	5.27E-15	Gm42517	protein_coding
ENSMUSG00000011751.16	14000.264	-0.373	0.044	-8.434	3.33E-17	8.53E-15	Sptbn4	protein_coding
ENSMUSG00000035828.10	1592.059	-0.365	0.043	-8.422	3.70E-17	9.32E-15	Pim3	protein_coding
ENSMUSG00000001227.11	5284.242	-0.295	0.035	-8.377	5.41E-17	1.34E-14	Sema6b	protein_coding
ENSMUSG00000035585.16	2037.478	-0.331	0.040	-8.355	6.53E-17	1.59E-14	Tsen34	protein_coding
ENSMUSG00000054364.5	7912.063	-0.257	0.031	-8.336	7.71E-17	1.84E-14	Rhob	protein_coding
ENSMUSG00000043439.5	2609.601	-0.491	0.059	-8.327	8.28E-17	1.95E-14	Epop	protein_coding
ENSMUSG00000024873.6	6806.672	-0.278	0.033	-8.317	9.02E-17	2.08E-14	Cnih2	protein_coding
ENSMUSG00000044927.6	359.776	-0.527	0.064	-8.183	2.77E-16	6.29E-14	H1fx	protein_coding
ENSMUSG00000003380.11	3581.323	-0.265	0.033	-8.126	4.46E-16	9.97E-14	Rabac1	protein_coding

ENSMUSG00000028478.18	6237.142	-0.283	0.035	-8.112	5.00E-16	1.10E-13	Clta	protein_coding
ENSMUSG00000048967.16	448.124	-0.537	0.066	-8.092	5.86E-16	1.27E-13	Yjefn3	protein_coding
ENSMUSG00000030685.5	3468.793	-0.267	0.033	-8.073	6.84E-16	1.46E-13	Kctd13	protein_coding
ENSMUSG00000042675.15	6175.722	-0.336	0.042	-8.069	7.12E-16	1.50E-13	Ypel3	protein_coding
ENSMUSG00000041697.8	6569.479	-0.313	0.039	-8.067	7.23E-16	1.50E-13	Cox6a1	protein_coding
ENSMUSG00000033467.12	518.566	-0.465	0.058	-8.011	1.13E-15	2.32E-13	Crlf2	protein_coding
ENSMUSG00000052684.4	2489.177	-0.333	0.042	-8.004	1.20E-15	2.42E-13	Jun	protein_coding
ENSMUSG00000036422.10	2306.449	-0.400	0.050	-7.956	1.77E-15	3.52E-13	Pcdh8	protein_coding
ENSMUSG00000078235.3	1323.520	-0.411	0.052	-7.933	2.14E-15	4.20E-13	Fam43b	protein_coding
ENSMUSG00000011096.17	1640.762	-0.410	0.052	-7.903	2.71E-15	5.24E-13	Akt1s1	protein_coding
ENSMUSG00000019261.3	3279.321	-0.348	0.044	-7.885	3.15E-15	5.93E-13	Map1s	protein_coding
ENSMUSG00000015476.6	6427.574	-0.286	0.036	-7.885	3.16E-15	5.93E-13	Prrt1	protein_coding
ENSMUSG00000052981.6	9679.894	-0.240	0.030	-7.878	3.32E-15	6.17E-13	Ube2ql1	protein_coding
ENSMUSG00000037236.14	14697.288	0.240	0.031	7.849	4.21E-15	7.70E-13	Matr3	protein_coding
ENSMUSG00000007950.9	10690.024	-0.328	0.042	-7.809	5.76E-15	1.04E-12	Abhd8	protein_coding
ENSMUSG00000028180.18	9904.013	0.178	0.023	7.796	6.38E-15	1.14E-12	Zranb2	protein_coding
ENSMUSG00000090877.3	687.482	-0.555	0.071	-7.784	7.01E-15	1.23E-12	Hspa1b	protein_coding
ENSMUSG00000033981.14	36232.451	0.181	0.023	7.760	8.46E-15	1.47E-12	Gria2	protein_coding
ENSMUSG00000038976.12	39823.755	-0.222	0.029	-7.757	8.67E-15	1.49E-12	Ppp1r9b	protein_coding
ENSMUSG00000003072.15	6475.690	-0.255	0.033	-7.743	9.71E-15	1.65E-12	Atp5d	protein_coding
ENSMUSG00000003346.14	4043.632	-0.330	0.043	-7.741	9.85E-15	1.65E-12	Abhd17a	protein_coding
ENSMUSG00000034201.13	3477.939	-0.333	0.043	-7.740	9.97E-15	1.65E-12	Gas2l1	protein_coding
ENSMUSG00000004366.3	7344.551	-0.371	0.048	-7.681	1.57E-14	2.58E-12	Sst	protein_coding
ENSMUSG00000020087.5	711.408	-0.418	0.054	-7.679	1.60E-14	2.59E-12	Tysnd1	protein_coding
ENSMUSG00000041801.5	908.207	-0.382	0.050	-7.670	1.72E-14	2.76E-12	Phlda3	protein_coding
ENSMUSG00000000088.7	3521.405	-0.289	0.038	-7.662	1.83E-14	2.90E-12	Cox5a	protein_coding
ENSMUSG00000002984.17	3028.449	-0.265	0.035	-7.609	2.76E-14	4.32E-12	Tomm40	protein_coding
ENSMUSG00000030120.14	15767.863	-0.222	0.029	-7.592	3.16E-14	4.89E-12	Mlf2	protein_coding
ENSMUSG00000030811.14	4286.799	-0.243	0.032	-7.567	3.81E-14	5.83E-12	Fbxl19	protein_coding
ENSMUSG00000042978.10	5699.351	-0.276	0.037	-7.539	4.73E-14	7.17E-12	Sbk1	protein_coding
ENSMUSG00000034854.8	1759.471	-0.296	0.039	-7.511	5.86E-14	8.78E-12	Mfsd12	protein_coding
ENSMUSG00000052584.9	1884.408	-0.279	0.037	-7.454	9.06E-14	1.34E-11	Serp2	protein_coding
ENSMUSG00000027677.17	10489.396	0.167	0.022	7.435	1.05E-13	1.54E-11	Ttc14	protein_coding

ENSMUSG00000048481.15	1577.978	-0.381	0.051	-7.433	1.06E-13	1.54E-11	Mypop	protein_coding
ENSMUSG00000037979.13	3574.295	-0.243	0.033	-7.422	1.15E-13	1.65E-11	Ccdc92	protein_coding
ENSMUSG00000022199.10	30829.541	-0.261	0.035	-7.422	1.16E-13	1.65E-11	Slc22a17	protein_coding
ENSMUSG00000028849.17	12137.928	-0.238	0.032	-7.415	1.22E-13	1.71E-11	Map7d1	protein_coding
ENSMUSG00000020436.17	7711.715	0.198	0.027	7.391	1.46E-13	2.02E-11	Gabrg2	protein_coding
ENSMUSG00000059991.7	2598.656	-0.319	0.043	-7.376	1.63E-13	2.23E-11	Nptx2	protein_coding
ENSMUSG00000031029.6	3853.029	-0.253	0.034	-7.335	2.22E-13	3.01E-11	Eif3f	protein_coding
ENSMUSG00000025422.9	55624.113	-0.251	0.034	-7.300	2.88E-13	3.87E-11	Agap2	protein_coding
ENSMUSG00000033730.3	4336.244	-0.379	0.052	-7.271	3.57E-13	4.75E-11	Egr3	protein_coding
ENSMUSG00000046160.6	3270.411	-0.395	0.054	-7.268	3.66E-13	4.82E-11	Olig1	protein_coding
ENSMUSG00000033751.5	1164.392	-0.343	0.047	-7.258	3.92E-13	5.11E-11	Gadd45gip1	protein_coding
ENSMUSG00000008140.17	7488.264	-0.245	0.034	-7.242	4.41E-13	5.71E-11	Emc10	protein_coding
ENSMUSG00000037428.14	10345.284	-0.378	0.052	-7.227	4.93E-13	6.32E-11	Vgf	protein_coding
ENSMUSG00000020477.10	538.020	-0.438	0.061	-7.226	4.98E-13	6.33E-11	Mrps24	protein_coding
ENSMUSG00000022769.7	728.806	-0.443	0.061	-7.200	6.01E-13	7.49E-11	Sdf2l1	protein_coding
ENSMUSG00000030678.7	7128.976	-0.231	0.032	-7.172	7.39E-13	9.13E-11	Maz	protein_coding
ENSMUSG00000058833.11	931.967	-0.369	0.051	-7.167	7.69E-13	9.42E-11	2810428I15Rik	protein_coding
ENSMUSG00000034730.16	27122.310	-0.276	0.039	-7.128	1.02E-12	1.24E-10	Adgrb1	protein_coding
ENSMUSG00000061451.13	11977.343	-0.251	0.035	-7.088	1.36E-12	1.64E-10	Tmem151a	protein_coding
ENSMUSG00000041740.16	11504.920	-0.149	0.021	-7.074	1.51E-12	1.80E-10	Rnf10	protein_coding
ENSMUSG00000024614.6	3589.782	0.224	0.032	7.069	1.56E-12	1.85E-10	Tmx3	protein_coding
ENSMUSG00000020331.9	4651.453	-0.258	0.037	-7.038	1.95E-12	2.29E-10	Hcn2	protein_coding
ENSMUSG00000048644.8	13242.837	-0.200	0.028	-7.026	2.13E-12	2.48E-10	Ctxn1	protein_coding
ENSMUSG00000055692.18	4008.625	-0.274	0.039	-6.977	3.01E-12	3.47E-10	Tmem191c	protein_coding
ENSMUSG00000055302.5	7188.557	-0.217	0.031	-6.975	3.07E-12	3.52E-10	Mrfap1	protein_coding
ENSMUSG00000029603.15	3810.347	-0.304	0.044	-6.973	3.11E-12	3.52E-10	Dtx1	protein_coding
ENSMUSG00000096847.1	8989.196	-0.249	0.036	-6.972	3.12E-12	3.52E-10	Tmem151b	protein_coding
ENSMUSG00000044876.15	1459.512	-0.308	0.044	-6.971	3.15E-12	3.52E-10	Zfp444	protein_coding
ENSMUSG00000004951.10	347.637	-0.536	0.077	-6.968	3.21E-12	3.56E-10	Hspb1	protein_coding
ENSMUSG00000034863.9	3777.063	-0.246	0.035	-6.962	3.36E-12	3.70E-10	Ano8	protein_coding
ENSMUSG00000040972.8	3318.215	-0.300	0.043	-6.945	3.78E-12	4.13E-10	Igsf21	protein_coding
ENSMUSG00000107002.1	1016.151	-0.440	0.064	-6.928	4.27E-12	4.60E-10	0610012G03Rik	protein_coding
ENSMUSG00000033735.9	929.202	-0.358	0.052	-6.928	4.28E-12	4.60E-10	Spr	protein_coding

ENSMUSG00000015094.16	8669.392	-0.187	0.027	-6.927	4.31E-12	4.60E-10	Npdc1	protein_coding
ENSMUSG00000052253.5	1244.723	-0.330	0.048	-6.922	4.46E-12	4.72E-10	Zfp622	protein_coding
ENSMUSG00000024790.7	351.811	-0.469	0.068	-6.906	4.99E-12	5.25E-10	Sac3d1	protein_coding
ENSMUSG00000021816.10	14173.920	0.181	0.026	6.855	7.13E-12	7.39E-10	Ppp3cb	protein_coding
ENSMUSG00000034168.7	3658.536	-0.354	0.052	-6.843	7.73E-12	7.95E-10	Irf2bpl	protein_coding
ENSMUSG00000073131.12	2191.714	0.247	0.036	6.827	8.65E-12	8.84E-10	Vma21	protein_coding
ENSMUSG00000051403.9	8650.197	-0.249	0.037	-6.805	1.01E-11	1.02E-09	Ppp1r37	protein_coding
ENSMUSG00000052135.8	733.321	-0.424	0.062	-6.802	1.03E-11	1.04E-09	Foxo6	protein_coding
ENSMUSG00000037843.6	1653.770	-0.429	0.063	-6.793	1.10E-11	1.10E-09	Vstm2l	protein_coding
ENSMUSG00000000838.17	3510.761	0.217	0.032	6.791	1.11E-11	1.11E-09	Fmr1	protein_coding
ENSMUSG00000007836.6	3841.505	-0.282	0.042	-6.785	1.16E-11	1.15E-09	Hnrnpa0	protein_coding
ENSMUSG00000040390.13	7779.121	-0.286	0.042	-6.784	1.17E-11	1.15E-09	Map3k10	protein_coding
ENSMUSG00000064115.13	9132.273	0.203	0.030	6.761	1.37E-11	1.33E-09	Cadm2	protein_coding
ENSMUSG00000050138.7	285.223	-0.517	0.077	-6.754	1.44E-11	1.39E-09	Kcnk12	protein_coding
ENSMUSG00000049086.8	1317.001	-0.429	0.064	-6.733	1.66E-11	1.59E-09	Bmyc	protein_coding
ENSMUSG00000039615.8	4325.493	-0.208	0.031	-6.728	1.72E-11	1.64E-09	Stub1	protein_coding
ENSMUSG00000015733.13	9149.727	0.160	0.024	6.701	2.07E-11	1.96E-09	Capza2	protein_coding
ENSMUSG00000030357.10	5983.919	-0.243	0.036	-6.681	2.37E-11	2.22E-09	Fkbp4	protein_coding
ENSMUSG00000041774.14	824.274	-0.345	0.052	-6.661	2.73E-11	2.55E-09	Ydjc	protein_coding
ENSMUSG00000029725.10	261.519	-0.493	0.074	-6.629	3.38E-11	3.14E-09	Ppp1r35	protein_coding
ENSMUSG00000078572.3	288.010	-0.446	0.067	-6.620	3.59E-11	3.31E-09	Ndufaf8	protein_coding
ENSMUSG00000022564.6	16238.624	-0.160	0.024	-6.611	3.81E-11	3.48E-09	Grina	protein_coding
ENSMUSG00000032532.7	7293.557	-0.486	0.074	-6.607	3.93E-11	3.58E-09	Cck	protein_coding
ENSMUSG00000032328.12	18240.021	0.163	0.025	6.600	4.12E-11	3.72E-09	Tmem30a	protein_coding
ENSMUSG00000061479.15	2394.735	-0.254	0.039	-6.598	4.17E-11	3.74E-09	Snrpa	protein_coding
ENSMUSG00000039568.6	4783.271	-0.225	0.034	-6.585	4.55E-11	4.06E-09	Ubald1	protein_coding
ENSMUSG00000032423.12	3578.828	0.226	0.035	6.538	6.25E-11	5.54E-09	Syncrip	protein_coding
ENSMUSG00000027223.15	16938.468	-0.190	0.029	-6.527	6.72E-11	5.92E-09	Mapk8ip1	protein_coding
ENSMUSG00000029823.16	9862.856	0.170	0.026	6.509	7.57E-11	6.63E-09	Luc7l2	protein_coding
ENSMUSG00000026384.13	5677.483	0.210	0.032	6.488	8.71E-11	7.58E-09	Ptpn4	protein_coding
ENSMUSG00000031807.10	741.956	-0.372	0.057	-6.484	8.93E-11	7.72E-09	Pgls	protein_coding
ENSMUSG00000053395.15	2532.831	-0.301	0.047	-6.468	9.93E-11	8.54E-09	Cacng8	protein_coding
ENSMUSG00000018199.8	3434.332	0.210	0.033	6.440	1.19E-10	1.02E-08	Trove2	protein_coding

ENSMUSG00000024925.10	398.444	-0.458	0.071	-6.439	1.20E-10	1.02E-08	Rnaseh2c	protein_coding
ENSMUSG00000015837.15	19576.624	-0.139	0.022	-6.435	1.23E-10	1.04E-08	Sqstm1	protein_coding
ENSMUSG00000028557.10	10518.446	0.160	0.025	6.434	1.24E-10	1.04E-08	Rnf11	protein_coding
ENSMUSG00000020108.4	1777.174	-0.341	0.053	-6.429	1.29E-10	1.07E-08	Ddit4	protein_coding
ENSMUSG00000014956.15	12436.469	0.205	0.032	6.403	1.53E-10	1.27E-08	Ppp1cb	protein_coding
ENSMUSG00000032997.16	3799.357	-0.309	0.048	-6.388	1.68E-10	1.39E-08	Chpf	protein_coding
ENSMUSG00000038462.3	3926.862	-0.229	0.036	-6.379	1.78E-10	1.46E-08	Uqcrfs1	protein_coding
ENSMUSG00000031229.16	11326.121	0.179	0.028	6.366	1.94E-10	1.58E-08	Atrx	protein_coding
ENSMUSG00000055407.14	8844.426	-0.177	0.028	-6.364	1.97E-10	1.60E-08	Map6	protein_coding
ENSMUSG00000021712.14	3636.288	0.216	0.034	6.362	1.99E-10	1.60E-08	Trim23	protein_coding
ENSMUSG00000034974.13	1424.494	-0.281	0.044	-6.347	2.20E-10	1.76E-08	Dapk3	protein_coding
ENSMUSG00000044352.6	4046.109	-0.256	0.040	-6.331	2.44E-10	1.94E-08	Sowaha	protein_coding
ENSMUSG00000055633.6	374.186	-0.443	0.070	-6.320	2.62E-10	2.08E-08	Zfp580	protein_coding
ENSMUSG00000050357.9	2527.478	-0.268	0.043	-6.306	2.87E-10	2.26E-08	Carmil2	protein_coding
ENSMUSG00000033768.16	16716.471	-0.266	0.042	-6.305	2.89E-10	2.26E-08	Nrxn2	protein_coding
ENSMUSG00000041817.14	3530.054	0.189	0.030	6.301	2.96E-10	2.31E-08	Fam169a	protein_coding
ENSMUSG00000042419.8	348.559	-0.419	0.067	-6.273	3.53E-10	2.73E-08	Nfkbil1	protein_coding
ENSMUSG00000043614.13	745.874	-0.363	0.058	-6.269	3.63E-10	2.80E-08	Vps37d	protein_coding
ENSMUSG00000025856.15	1647.820	-0.263	0.042	-6.244	4.25E-10	3.26E-08	Pdgfa	protein_coding
ENSMUSG00000075012.4	2030.838	-0.313	0.050	-6.240	4.36E-10	3.33E-08	Fjx1	protein_coding
ENSMUSG00000002409.18	1582.798	-0.275	0.044	-6.239	4.41E-10	3.34E-08	Dyrk1b	protein_coding
ENSMUSG00000024736.14	16625.506	-0.263	0.042	-6.222	4.92E-10	3.71E-08	Tmem132a	protein_coding
ENSMUSG00000031517.8	55977.624	0.177	0.029	6.217	5.07E-10	3.80E-08	Gpm6a	protein_coding
ENSMUSG00000015599.8	7553.553	-0.209	0.034	-6.207	5.39E-10	4.01E-08	Ttbk1	protein_coding
ENSMUSG00000056665.2	1334.198	-0.282	0.045	-6.207	5.41E-10	4.01E-08	Them6	protein_coding
ENSMUSG00000042831.13	1036.375	-0.295	0.047	-6.206	5.44E-10	4.01E-08	Alkbh6	protein_coding
ENSMUSG00000074576.4	308.268	-0.423	0.068	-6.190	6.02E-10	4.42E-08	Mocs3	protein_coding
ENSMUSG00000059326.6	1431.179	-0.359	0.058	-6.177	6.53E-10	4.77E-08	Csf2ra	protein_coding
ENSMUSG00000035236.17	4391.132	0.198	0.032	6.170	6.85E-10	4.97E-08	Scai	protein_coding
ENSMUSG00000091971.3	486.491	-0.441	0.072	-6.165	7.05E-10	5.09E-08	Hspa1a	protein_coding
ENSMUSG00000025318.13	10013.596	-0.245	0.040	-6.159	7.34E-10	5.28E-08	Jph3	protein_coding
ENSMUSG00000033316.14	7251.847	-0.180	0.029	-6.157	7.43E-10	5.31E-08	Galnt9	protein_coding
ENSMUSG00000000948.16	469.647	0.364	0.059	6.144	8.03E-10	5.72E-08	Gm38393	protein_coding

ENSMUSG00000036430.8	527.997	-0.398	0.065	-6.132	8.68E-10	6.15E-08	Tbcc	protein_coding
ENSMUSG00000005483.10	4357.706	-0.223	0.036	-6.125	9.08E-10	6.40E-08	Dnajb1	protein_coding
ENSMUSG00000021098.14	2293.258	0.231	0.038	6.116	9.57E-10	6.71E-08	4930447C04Rik	protein_coding
ENSMUSG00000043323.16	3537.032	-0.296	0.048	-6.098	1.07E-09	7.50E-08	Fbrsl1	protein_coding
ENSMUSG00000025326.12	6180.965	0.185	0.030	6.083	1.18E-09	8.18E-08	Ube3a	protein_coding
ENSMUSG00000058301.8	4782.017	-0.166	0.027	-6.071	1.27E-09	8.81E-08	Upf1	protein_coding
ENSMUSG00000022436.15	8130.343	-0.182	0.030	-6.061	1.36E-09	9.32E-08	Sh3bp1	protein_coding
ENSMUSG00000056596.8	3908.967	-0.192	0.032	-6.060	1.36E-09	9.32E-08	Trnp1	protein_coding
ENSMUSG00000078317.6	658.459	-0.361	0.060	-6.027	1.67E-09	1.14E-07	F8a	protein_coding
ENSMUSG00000063160.12	6673.029	-0.228	0.038	-6.025	1.69E-09	1.15E-07	Numbl	protein_coding
ENSMUSG00000031290.14	1214.162	0.279	0.046	6.015	1.79E-09	1.20E-07	Lrch2	protein_coding
ENSMUSG00000062044.15	15185.482	-0.269	0.045	-6.002	1.95E-09	1.30E-07	Lmtk3	protein_coding
ENSMUSG00000020300.14	5960.585	0.171	0.029	5.992	2.08E-09	1.38E-07	Cpeb4	protein_coding
ENSMUSG00000044308.17	10143.177	0.146	0.024	5.987	2.13E-09	1.41E-07	Ubr3	protein_coding
ENSMUSG00000071337.11	6733.098	0.183	0.031	5.980	2.23E-09	1.47E-07	Tia1	protein_coding
ENSMUSG00000094724.7	84.731	-0.455	0.076	-5.961	2.51E-09	1.64E-07	Rnaset2b	protein_coding
ENSMUSG00000051223.14	9029.391	0.155	0.026	5.952	2.65E-09	1.73E-07	Bzw1	protein_coding
ENSMUSG00000019039.13	3290.749	-0.218	0.037	-5.945	2.77E-09	1.80E-07	Dalrd3	protein_coding
ENSMUSG00000010803.13	16408.218	0.175	0.030	5.939	2.86E-09	1.85E-07	Gabra1	protein_coding
ENSMUSG00000040097.15	13012.460	-0.184	0.031	-5.930	3.02E-09	1.95E-07	Flywch1	protein_coding
ENSMUSG00000025034.8	2860.118	-0.266	0.045	-5.923	3.17E-09	2.03E-07	Trim8	protein_coding
ENSMUSG00000090125.3	821.213	-0.352	0.059	-5.922	3.18E-09	2.03E-07	Pou3f1	protein_coding
ENSMUSG00000038880.13	994.468	-0.328	0.055	-5.921	3.21E-09	2.04E-07	Mrps34	protein_coding
ENSMUSG00000035027.18	3835.513	-0.199	0.034	-5.912	3.37E-09	2.13E-07	Map2k2	protein_coding
ENSMUSG00000002107.18	24736.775	0.148	0.025	5.910	3.42E-09	2.15E-07	Celf2	protein_coding
ENSMUSG00000069045.11	2655.272	0.216	0.037	5.869	4.39E-09	2.75E-07	Ddx3y	protein_coding
ENSMUSG00000029267.17	2888.283	0.226	0.039	5.867	4.44E-09	2.77E-07	Mtf2	protein_coding
ENSMUSG00000051067.8	1775.494	-0.320	0.055	-5.865	4.50E-09	2.79E-07	Lingo3	protein_coding
ENSMUSG00000038453.16	22631.224	-0.187	0.032	-5.859	4.65E-09	2.86E-07	Srcin1	protein_coding
ENSMUSG00000028134.11	4494.322	0.174	0.030	5.859	4.65E-09	2.86E-07	Ptbp2	protein_coding
ENSMUSG00000041268.17	18258.846	0.122	0.021	5.851	4.90E-09	3.00E-07	Dmxl2	protein_coding
ENSMUSG00000003814.8	18503.463	-0.164	0.028	-5.841	5.18E-09	3.16E-07	Calr	protein_coding
ENSMUSG00000028782.14	27737.876	-0.206	0.035	-5.839	5.26E-09	3.20E-07	Adgrb2	protein_coding

ENSMUSG00000045763.7	14642.200	-0.293	0.050	-5.837	5.32E-09	3.21E-07	Basp1	protein_coding
ENSMUSG00000052372.10	732.179	0.336	0.058	5.836	5.33E-09	3.21E-07	Il1rapl1	protein_coding
ENSMUSG00000095687.1	105.105	-0.451	0.077	-5.831	5.52E-09	3.31E-07	Rnaset2a	protein_coding
ENSMUSG00000003452.15	2465.031	0.213	0.037	5.800	6.63E-09	3.96E-07	Bicd1	protein_coding
ENSMUSG00000020441.6	598.194	-0.335	0.058	-5.799	6.66E-09	3.96E-07	2310033P09Rik	protein_coding
ENSMUSG00000074405.7	1720.240	-0.275	0.047	-5.798	6.71E-09	3.97E-07	Zfp865	protein_coding
ENSMUSG00000020572.8	2773.572	0.203	0.035	5.794	6.86E-09	4.04E-07	Nampt	protein_coding
ENSMUSG00000031198.4	1380.431	0.254	0.044	5.782	7.38E-09	4.31E-07	Fundc2	protein_coding
ENSMUSG00000008036.11	4712.806	-0.192	0.033	-5.782	7.40E-09	4.31E-07	Ap2s1	protein_coding
ENSMUSG00000049295.16	1721.449	-0.263	0.045	-5.775	7.70E-09	4.45E-07	Zfp219	protein_coding
ENSMUSG00000034755.18	794.634	0.320	0.055	5.775	7.71E-09	4.45E-07	Pcdh11x	protein_coding
ENSMUSG00000040118.15	10049.579	0.213	0.037	5.768	8.01E-09	4.61E-07	Cacna2d1	protein_coding
ENSMUSG00000038255.6	3058.671	-0.299	0.052	-5.766	8.10E-09	4.64E-07	Neurod2	protein_coding
ENSMUSG00000018846.8	3644.024	0.198	0.034	5.764	8.23E-09	4.70E-07	Pank3	protein_coding
ENSMUSG00000034949.18	4768.263	-0.230	0.040	-5.739	9.53E-09	5.40E-07	Zfr2	protein_coding
ENSMUSG00000026034.17	7364.418	0.166	0.029	5.739	9.54E-09	5.40E-07	Clk1	protein_coding
ENSMUSG00000035890.9	2361.305	-0.244	0.043	-5.737	9.63E-09	5.43E-07	Rnf126	protein_coding
ENSMUSG00000053080.10	2091.640	-0.242	0.042	-5.726	1.03E-08	5.78E-07	2700081O15Rik	protein_coding
ENSMUSG00000068267.5	6104.550	-0.187	0.033	-5.725	1.03E-08	5.78E-07	Cenpb	protein_coding
ENSMUSG00000079658.9	4230.038	0.196	0.034	5.722	1.05E-08	5.86E-07	Eloc	protein_coding
ENSMUSG00000039556.16	219.852	-0.434	0.076	-5.718	1.08E-08	5.95E-07	Ppp1r3f	protein_coding
ENSMUSG00000048385.8	4629.500	-0.289	0.050	-5.718	1.08E-08	5.95E-07	Scrt1	protein_coding
ENSMUSG00000067786.16	5994.250	-0.314	0.055	-5.714	1.10E-08	6.07E-07	Nnat	protein_coding
ENSMUSG00000038406.17	9771.705	-0.185	0.032	-5.707	1.15E-08	6.31E-07	Scaf1	protein_coding
ENSMUSG00000053166.14	1615.999	-0.317	0.056	-5.706	1.16E-08	6.33E-07	Cdh22	protein_coding
ENSMUSG00000087403.9	1788.113	0.212	0.037	5.700	1.20E-08	6.51E-07	Kantr	protein_coding
ENSMUSG00000026223.15	32457.444	-0.155	0.027	-5.700	1.20E-08	6.51E-07	Itm2c	protein_coding
ENSMUSG00000040659.3	14846.723	-0.157	0.028	-5.691	1.27E-08	6.84E-07	Efhd2	protein_coding
ENSMUSG00000021288.18	31753.493	-0.161	0.028	-5.687	1.30E-08	6.97E-07	Klc1	protein_coding
ENSMUSG00000021687.14	10555.336	0.133	0.023	5.683	1.33E-08	7.11E-07	Scamp1	protein_coding
ENSMUSG00000021508.10	2905.289	-0.278	0.049	-5.652	1.59E-08	8.48E-07	Cxcl14	protein_coding
ENSMUSG00000022208.11	20087.777	-0.195	0.035	-5.651	1.59E-08	8.48E-07	Jph4	protein_coding
ENSMUSG00000071637.5	99.518	-0.412	0.073	-5.644	1.66E-08	8.78E-07	Cebpd	protein_coding

ENSMUSG00000049807.16	7991.453	-0.216	0.038	-5.641	1.69E-08	8.92E-07	Arhgap23	protein_coding
ENSMUSG00000004931.11	1109.715	-0.310	0.055	-5.641	1.70E-08	8.92E-07	Apba3	protein_coding
ENSMUSG00000046321.8	3321.863	-0.266	0.047	-5.632	1.78E-08	9.33E-07	Hs3st2	protein_coding
ENSMUSG00000037458.14	8412.219	0.147	0.026	5.628	1.82E-08	9.49E-07	Azin1	protein_coding
ENSMUSG00000034336.3	10806.241	-0.172	0.031	-5.627	1.84E-08	9.55E-07	Ina	protein_coding
ENSMUSG00000037771.11	4816.919	-0.326	0.058	-5.624	1.86E-08	9.66E-07	Slc32a1	protein_coding
ENSMUSG00000045176.3	920.352	-0.325	0.058	-5.622	1.89E-08	9.71E-07	Borcs6	protein_coding
ENSMUSG00000024772.9	1644.249	-0.236	0.042	-5.622	1.89E-08	9.71E-07	Ehd1	protein_coding
ENSMUSG00000060152.14	1332.594	-0.261	0.047	-5.604	2.09E-08	1.07E-06	Pop5	protein_coding
ENSMUSG00000031246.14	3153.318	0.203	0.036	5.603	2.11E-08	1.08E-06	Sh3bgrl	protein_coding
ENSMUSG00000037904.14	399.479	-0.382	0.068	-5.583	2.37E-08	1.20E-06	Ankrd9	protein_coding
ENSMUSG00000048920.8	1834.603	-0.229	0.041	-5.568	2.58E-08	1.31E-06	Fkrp	protein_coding
ENSMUSG00000025531.14	2657.289	0.199	0.036	5.564	2.64E-08	1.33E-06	Chm	protein_coding
ENSMUSG00000046658.16	1330.931	-0.243	0.044	-5.560	2.70E-08	1.36E-06	Zfp316	protein_coding
ENSMUSG00000035285.6	3131.244	-0.234	0.042	-5.553	2.81E-08	1.41E-06	Nat14	protein_coding
ENSMUSG00000020483.14	23272.940	-0.119	0.021	-5.547	2.90E-08	1.45E-06	Dynll2	protein_coding
ENSMUSG00000045348.16	5271.072	-0.174	0.031	-5.546	2.92E-08	1.46E-06	Nyap1	protein_coding
ENSMUSG00000037217.15	31058.872	-0.196	0.035	-5.537	3.07E-08	1.52E-06	Syn1	protein_coding
ENSMUSG00000033953.10	26464.608	0.163	0.029	5.528	3.24E-08	1.60E-06	Ppp3r1	protein_coding
ENSMUSG00000030397.10	4389.887	-0.200	0.036	-5.523	3.32E-08	1.64E-06	Mark4	protein_coding
ENSMUSG00000025903.14	723.674	0.313	0.057	5.519	3.41E-08	1.68E-06	Lypla1	protein_coding
ENSMUSG00000002504.14	2214.771	-0.243	0.044	-5.518	3.43E-08	1.68E-06	Slc9a3r2	protein_coding
ENSMUSG00000015337.5	358.345	-0.382	0.069	-5.513	3.52E-08	1.72E-06	Endog	protein_coding
ENSMUSG00000026761.12	2719.665	0.201	0.036	5.512	3.55E-08	1.73E-06	Orc4	protein_coding
ENSMUSG00000054708.17	2697.355	-0.223	0.040	-5.502	3.75E-08	1.82E-06	Ankrd24	protein_coding
ENSMUSG00000060708.4	318.361	-0.382	0.069	-5.500	3.80E-08	1.83E-06	Bloc1s4	protein_coding
ENSMUSG00000075467.4	1622.585	-0.244	0.044	-5.498	3.83E-08	1.84E-06	Dnlz	protein_coding
ENSMUSG00000058709.11	3548.943	-0.197	0.036	-5.495	3.91E-08	1.88E-06	Egln2	protein_coding
ENSMUSG00000070705.3	990.083	-0.275	0.050	-5.484	4.15E-08	1.98E-06	Eid2b	protein_coding
ENSMUSG00000022338.6	2616.035	0.185	0.034	5.481	4.24E-08	2.02E-06	Eny2	protein_coding
ENSMUSG00000068457.14	856.903	0.278	0.051	5.480	4.26E-08	2.02E-06	Uty	protein_coding
ENSMUSG00000035992.15	3098.212	0.184	0.034	5.475	4.36E-08	2.06E-06	Fnip1	protein_coding
ENSMUSG00000023272.3	1147.467	-0.253	0.046	-5.468	4.55E-08	2.13E-06	Creld2	protein_coding

ENSMUSG00000022623.15	12024.963	-0.231	0.042	-5.468	4.56E-08	2.13E-06	Shank3	protein_coding
ENSMUSG00000024121.13	2237.865	-0.224	0.041	-5.468	4.56E-08	2.13E-06	Atp6v0c	protein_coding
ENSMUSG00000085795.8	1446.007	-0.297	0.054	-5.466	4.61E-08	2.15E-06	Zfp703	protein_coding
ENSMUSG00000041556.8	1977.040	-0.255	0.047	-5.455	4.90E-08	2.28E-06	Fbxo2	protein_coding
ENSMUSG00000033701.13	1654.348	-0.210	0.038	-5.454	4.93E-08	2.28E-06	Acbd6	protein_coding
ENSMUSG00000042208.15	2290.630	0.193	0.036	5.448	5.08E-08	2.35E-06	0610010F05Rik	protein_coding
ENSMUSG00000032575.16	2227.003	-0.266	0.049	-5.448	5.09E-08	2.35E-06	Manf	protein_coding
ENSMUSG00000026977.17	3132.445	0.178	0.033	5.439	5.35E-08	2.46E-06	March7	protein_coding
ENSMUSG00000094483.2	22417.478	0.165	0.030	5.428	5.69E-08	2.60E-06	Purb	protein_coding
ENSMUSG00000027602.9	4853.485	-0.180	0.033	-5.425	5.80E-08	2.65E-06	Map1lc3a	protein_coding
ENSMUSG00000036745.15	12721.836	0.138	0.025	5.409	6.34E-08	2.88E-06	Ttll7	protein_coding
ENSMUSG00000046449.15	2597.548	0.190	0.035	5.400	6.66E-08	3.02E-06	C77370	protein_coding
ENSMUSG00000040459.11	7215.855	0.188	0.035	5.395	6.85E-08	3.10E-06	Arglu1	protein_coding
ENSMUSG00000004929.12	2702.844	-0.232	0.043	-5.392	6.99E-08	3.15E-06	Thop1	protein_coding
ENSMUSG00000027589.14	4786.133	0.149	0.028	5.386	7.21E-08	3.22E-06	Pcmtd2	protein_coding
ENSMUSG00000051695.6	5522.616	-0.226	0.042	-5.383	7.31E-08	3.26E-06	Pcbp1	protein_coding
ENSMUSG00000036622.15	8902.447	-0.245	0.045	-5.381	7.40E-08	3.29E-06	Atp13a2	protein_coding
ENSMUSG00000041309.17	320.631	-0.406	0.076	-5.368	7.94E-08	3.52E-06	Nkx6-2	protein_coding
ENSMUSG00000000787.12	10537.439	0.143	0.027	5.364	8.12E-08	3.59E-06	Ddx3x	protein_coding
ENSMUSG00000038916.7	2861.898	-0.210	0.039	-5.363	8.18E-08	3.60E-06	Soga3	protein_coding
ENSMUSG00000045318.6	685.804	-0.338	0.063	-5.359	8.36E-08	3.67E-06	Adra2c	protein_coding
ENSMUSG00000051703.14	2997.577	-0.244	0.045	-5.358	8.43E-08	3.69E-06	Tmem198	protein_coding
ENSMUSG00000024962.13	1859.051	-0.253	0.047	-5.347	8.95E-08	3.90E-06	Vegfb	protein_coding
ENSMUSG00000038174.14	6834.272	0.178	0.033	5.342	9.18E-08	3.99E-06	Fam126b	protein_coding
ENSMUSG00000033186.8	3199.245	0.179	0.034	5.341	9.22E-08	3.99E-06	Mzt1	protein_coding
ENSMUSG00000052516.17	4902.321	0.161	0.030	5.341	9.23E-08	3.99E-06	Robo2	protein_coding
ENSMUSG00000021087.18	48418.926	-0.122	0.023	-5.330	9.81E-08	4.23E-06	Rtn1	protein_coding
ENSMUSG00000015092.9	3057.939	-0.203	0.038	-5.320	1.04E-07	4.46E-06	Edf1	protein_coding
ENSMUSG00000056708.5	3385.904	-0.301	0.057	-5.319	1.04E-07	4.46E-06	Ier5	protein_coding
ENSMUSG00000046470.5	210.046	-0.413	0.078	-5.314	1.07E-07	4.57E-06	Sox18	protein_coding
ENSMUSG00000002763.16	2890.377	-0.212	0.040	-5.314	1.07E-07	4.57E-06	Pex6	protein_coding
ENSMUSG00000039195.4	3311.054	-0.215	0.040	-5.311	1.09E-07	4.63E-06	1110008P14Rik	protein_coding
ENSMUSG00000066721.3	414.366	-0.331	0.062	-5.308	1.11E-07	4.69E-06	Zfp575	protein_coding

ENSMUSG00000009207.15	2318.693	0.194	0.037	5.304	1.13E-07	4.77E-06	Lnpk	protein_coding
ENSMUSG00000029050.15	8189.933	-0.147	0.028	-5.304	1.13E-07	4.77E-06	Ski	protein_coding
ENSMUSG00000026775.9	4447.565	0.153	0.029	5.294	1.20E-07	5.03E-06	Yme1l1	protein_coding
ENSMUSG00000036353.13	1322.658	0.254	0.048	5.293	1.21E-07	5.04E-06	P2ry12	protein_coding
ENSMUSG00000037740.8	1717.082	-0.227	0.043	-5.287	1.24E-07	5.19E-06	Mrps26	protein_coding
ENSMUSG00000078566.8	4013.503	0.168	0.032	5.275	1.33E-07	5.51E-06	Bnip3	protein_coding
ENSMUSG00000020167.14	1439.219	-0.229	0.044	-5.263	1.42E-07	5.88E-06	Tcf3	protein_coding
ENSMUSG00000022463.7	13537.677	-0.137	0.026	-5.262	1.43E-07	5.89E-06	Srebf2	protein_coding
ENSMUSG00000055531.12	6485.973	0.143	0.027	5.258	1.46E-07	6.00E-06	Cpsf6	protein_coding
ENSMUSG00000034675.17	15930.776	-0.167	0.032	-5.256	1.47E-07	6.05E-06	Dbn1	protein_coding
ENSMUSG00000019139.10	1684.677	-0.283	0.054	-5.255	1.48E-07	6.06E-06	Isyna1	protein_coding
ENSMUSG00000037126.15	20850.649	-0.206	0.039	-5.251	1.51E-07	6.17E-06	Psd	protein_coding
ENSMUSG00000030847.8	358.786	-0.339	0.065	-5.248	1.54E-07	6.26E-06	Bag3	protein_coding
ENSMUSG00000030846.15	5172.613	0.147	0.028	5.245	1.56E-07	6.35E-06	Tial1	protein_coding
ENSMUSG00000035640.18	14251.594	-0.216	0.041	-5.240	1.61E-07	6.51E-06	Cbarp	protein_coding
ENSMUSG00000030759.16	6813.646	0.150	0.029	5.230	1.70E-07	6.84E-06	Far1	protein_coding
ENSMUSG00000050761.3	275.782	-0.392	0.075	-5.226	1.73E-07	6.95E-06	Gp1bb	protein_coding
ENSMUSG00000056724.14	2154.307	-0.204	0.039	-5.221	1.78E-07	7.13E-06	Nbeal2	protein_coding
ENSMUSG00000056917.12	795.472	-0.309	0.059	-5.218	1.80E-07	7.20E-06	Sipa1	protein_coding
ENSMUSG00000067847.13	632.458	-0.326	0.063	-5.212	1.87E-07	7.44E-06	Romo1	protein_coding
ENSMUSG00000018293.4	3211.829	-0.247	0.047	-5.210	1.89E-07	7.51E-06	Pfn1	protein_coding
ENSMUSG00000025876.15	7317.289	-0.199	0.038	-5.209	1.90E-07	7.51E-06	Unc5a	protein_coding
ENSMUSG00000066324.2	5860.911	0.159	0.031	5.206	1.93E-07	7.63E-06	Impad1	protein_coding
ENSMUSG00000020734.13	2767.447	-0.212	0.041	-5.204	1.95E-07	7.66E-06	Grin2c	protein_coding
ENSMUSG00000039477.16	7457.360	-0.220	0.042	-5.202	1.97E-07	7.72E-06	Tnrc18	protein_coding
ENSMUSG00000035967.15	5004.849	0.156	0.030	5.202	1.97E-07	7.72E-06	Ints6l	protein_coding
ENSMUSG00000028851.6	3810.918	-0.168	0.032	-5.200	1.99E-07	7.79E-06	Nudc	protein_coding
ENSMUSG00000051515.9	535.314	-0.323	0.062	-5.197	2.02E-07	7.88E-06	Fam181b	protein_coding
ENSMUSG00000056501.3	54.047	-0.389	0.075	-5.193	2.07E-07	NA	Cebpb	protein_coding
ENSMUSG00000022378.13	6416.637	0.159	0.031	5.193	2.07E-07	8.04E-06	Fam49b	protein_coding
ENSMUSG00000032115.14	8110.712	-0.147	0.028	-5.190	2.11E-07	8.15E-06	Hyou1	protein_coding
ENSMUSG00000002274.12	1329.323	-0.315	0.061	-5.185	2.16E-07	8.33E-06	Metrn	protein_coding
ENSMUSG00000056342.16	11761.357	0.126	0.024	5.181	2.21E-07	8.51E-06	Usp34	protein_coding

ENSMUSG00000001376.17	4005.142	0.163	0.032	5.148	2.64E-07	1.01E-05	Vps50	protein_coding
ENSMUSG00000027692.16	12325.366	0.120	0.023	5.147	2.65E-07	1.01E-05	Tnik	protein_coding
ENSMUSG00000020232.17	579.014	-0.308	0.060	-5.147	2.65E-07	1.01E-05	Hmg20b	protein_coding
ENSMUSG00000025375.15	20218.030	-0.175	0.034	-5.144	2.68E-07	1.02E-05	Aatk	protein_coding
ENSMUSG00000037234.17	7525.498	0.127	0.025	5.140	2.75E-07	1.04E-05	Hook3	protein_coding
ENSMUSG00000036438.12	56102.042	0.127	0.025	5.133	2.86E-07	1.08E-05	Calm2	protein_coding
ENSMUSG00000032407.14	3961.227	0.160	0.031	5.131	2.88E-07	1.09E-05	U2surp	protein_coding
ENSMUSG00000055148.7	294.128	-0.388	0.076	-5.124	3.00E-07	1.13E-05	Klf2	protein_coding
ENSMUSG00000049556.5	14621.710	-0.207	0.040	-5.123	3.01E-07	1.13E-05	Lingo1	protein_coding
ENSMUSG00000006435.15	10351.969	-0.162	0.032	-5.121	3.04E-07	1.14E-05	Neurl1a	protein_coding
ENSMUSG00000030034.11	470.611	-0.339	0.066	-5.118	3.09E-07	1.15E-05	Ino80b	protein_coding
ENSMUSG00000037060.3	536.011	-0.327	0.064	-5.113	3.17E-07	1.18E-05	Prkcdbp	protein_coding
ENSMUSG00000031292.14	10932.327	0.128	0.025	5.096	3.46E-07	1.28E-05	Cdkl5	protein_coding
ENSMUSG00000042116.3	1488.888	-0.245	0.048	-5.096	3.47E-07	1.28E-05	Vwa1	protein_coding
ENSMUSG00000031834.15	7480.213	-0.167	0.033	-5.096	3.48E-07	1.28E-05	Pik3r2	protein_coding
ENSMUSG00000030376.8	19848.950	-0.153	0.030	-5.096	3.48E-07	1.28E-05	Slc8a2	protein_coding
ENSMUSG00000046822.12	4720.074	-0.163	0.032	-5.095	3.48E-07	1.28E-05	Slc39a3	protein_coding
ENSMUSG00000033510.14	2291.394	-0.187	0.037	-5.092	3.54E-07	1.30E-05	Otud7a	protein_coding
ENSMUSG00000025825.12	2008.505	-0.187	0.037	-5.088	3.62E-07	1.32E-05	Iscu	protein_coding
ENSMUSG00000041560.12	2512.904	-0.209	0.041	-5.085	3.67E-07	1.34E-05	Nop53	protein_coding
ENSMUSG00000046574.8	4281.415	-0.157	0.031	-5.078	3.81E-07	1.39E-05	Prr12	protein_coding
ENSMUSG00000063765.11	516.745	-0.335	0.066	-5.076	3.86E-07	1.40E-05	Chadl	protein_coding
ENSMUSG00000021693.19	4302.149	0.154	0.030	5.066	4.07E-07	1.47E-05	Kif2a	protein_coding
ENSMUSG00000029463.5	1956.332	-0.204	0.040	-5.064	4.11E-07	1.49E-05	Fam216a	protein_coding
ENSMUSG00000046699.14	2150.233	0.227	0.045	5.057	4.25E-07	1.53E-05	Slitrk4	protein_coding
ENSMUSG00000032485.14	5744.882	-0.138	0.027	-5.052	4.37E-07	1.57E-05	Scap	protein_coding
ENSMUSG00000023845.6	2653.618	0.185	0.037	5.051	4.40E-07	1.58E-05	Lnpep	protein_coding
ENSMUSG00000026739.13	2076.753	0.185	0.037	5.033	4.82E-07	1.72E-05	Bmi1	protein_coding
ENSMUSG00000023353.14	12847.615	-0.169	0.034	-5.032	4.85E-07	1.73E-05	Agap3	protein_coding
ENSMUSG00000038550.10	753.517	0.264	0.052	5.031	4.87E-07	1.73E-05	Ciart	protein_coding
ENSMUSG00000020152.7	13507.899	0.134	0.027	5.031	4.88E-07	1.73E-05	Actr2	protein_coding
ENSMUSG00000022175.8	1373.659	-0.223	0.044	-5.028	4.95E-07	1.75E-05	Lrp10	protein_coding
ENSMUSG00000087006.3	626.778	-0.335	0.067	-5.028	4.95E-07	1.75E-05	Gm13889	protein_coding

ENSMUSG00000000202.9	458.018	-0.328	0.065	-5.020	5.17E-07	1.82E-05	Btbd17	protein_coding
ENSMUSG00000020362.13	3115.284	0.171	0.034	5.016	5.27E-07	1.85E-05	Cnot6	protein_coding
ENSMUSG00000096768.7	261.480	-0.321	0.064	-5.014	5.32E-07	1.86E-05	Erdr1	protein_coding
ENSMUSG00000029478.16	13430.336	-0.221	0.044	-5.011	5.41E-07	1.89E-05	Ncor2	protein_coding
ENSMUSG00000021134.17	14604.316	0.139	0.028	5.011	5.43E-07	1.89E-05	Srsf5	protein_coding
ENSMUSG00000037608.16	7420.576	0.163	0.033	5.010	5.43E-07	1.89E-05	Bclaf1	protein_coding
ENSMUSG00000091811.2	933.535	-0.243	0.048	-5.010	5.46E-07	1.89E-05	Inafm1	protein_coding
ENSMUSG00000031878.18	2484.679	0.184	0.037	5.008	5.51E-07	1.90E-05	Nae1	protein_coding
ENSMUSG00000022565.15	16094.195	-0.198	0.039	-5.007	5.53E-07	1.91E-05	Plec	protein_coding
ENSMUSG00000048616.4	242.835	-0.370	0.074	-5.003	5.64E-07	1.94E-05	Nog	protein_coding
ENSMUSG00000020687.13	3395.348	0.157	0.031	5.001	5.71E-07	1.96E-05	Cdc27	protein_coding
ENSMUSG00000038034.15	10518.716	-0.185	0.037	-4.995	5.88E-07	2.01E-05	Igsf8	protein_coding
ENSMUSG00000071172.12	6515.638	0.145	0.029	4.987	6.13E-07	2.09E-05	Srsf3	protein_coding
ENSMUSG00000042570.14	2205.728	-0.228	0.046	-4.985	6.21E-07	2.11E-05	Mier2	protein_coding
ENSMUSG00000040855.15	13418.012	0.150	0.030	4.982	6.31E-07	2.14E-05	Reps2	protein_coding
ENSMUSG00000031565.18	4317.262	-0.197	0.040	-4.980	6.37E-07	2.16E-05	Fgfr1	protein_coding
ENSMUSG00000027087.11	3732.754	0.154	0.031	4.977	6.47E-07	2.19E-05	Itgav	protein_coding
ENSMUSG00000058441.7	4556.246	-0.194	0.039	-4.972	6.62E-07	2.23E-05	Panx2	protein_coding
ENSMUSG00000025384.15	1196.626	-0.252	0.051	-4.971	6.66E-07	2.24E-05	Faap100	protein_coding
ENSMUSG00000042745.9	339.512	-0.359	0.072	-4.970	6.69E-07	2.24E-05	Id1	protein_coding
ENSMUSG00000044024.15	5485.464	-0.217	0.044	-4.963	6.92E-07	2.32E-05	Rell2	protein_coding
ENSMUSG00000019194.15	9276.650	-0.162	0.033	-4.961	7.02E-07	2.34E-05	Scn1b	protein_coding
ENSMUSG00000059213.6	50720.774	-0.191	0.038	-4.959	7.07E-07	2.36E-05	Ddn	protein_coding
ENSMUSG00000025135.12	1345.399	-0.209	0.042	-4.954	7.29E-07	2.42E-05	Anapc11	protein_coding
ENSMUSG00000029655.17	2534.672	0.171	0.035	4.952	7.34E-07	2.43E-05	N4bp2l2	protein_coding
ENSMUSG00000043535.13	5199.795	0.138	0.028	4.952	7.36E-07	2.44E-05	Setx	protein_coding
ENSMUSG00000047067.7	734.036	-0.287	0.058	-4.950	7.42E-07	2.45E-05	Dusp28	protein_coding
ENSMUSG00000004151.17	3418.371	0.187	0.038	4.945	7.61E-07	2.51E-05	Etv1	protein_coding
ENSMUSG00000047428.14	2016.425	-0.227	0.046	-4.943	7.71E-07	2.53E-05	Dlk2	protein_coding
ENSMUSG00000021775.10	6286.107	0.173	0.035	4.940	7.80E-07	2.56E-05	Nr1d2	protein_coding
ENSMUSG00000035133.9	6349.622	0.142	0.029	4.939	7.84E-07	2.56E-05	Arhgap5	protein_coding
ENSMUSG00000031833.10	17478.809	-0.162	0.033	-4.935	8.00E-07	2.61E-05	Mast3	protein_coding
ENSMUSG00000029030.14	6057.226	-0.144	0.029	-4.931	8.18E-07	2.66E-05	Tprgl	protein_coding

ENSMUSG00000038777.19	1051.875	-0.252	0.051	-4.930	8.21E-07	2.66E-05	Sema6c	protein_coding
ENSMUSG00000093803.3	830.699	-0.278	0.056	-4.930	8.22E-07	2.66E-05	Ppp2r3d	protein_coding
ENSMUSG00000036990.13	4374.919	0.148	0.030	4.925	8.44E-07	2.72E-05	Otud4	protein_coding
ENSMUSG00000038884.14	4858.358	-0.162	0.033	-4.924	8.47E-07	2.73E-05	A230050P20Rik	protein_coding
ENSMUSG00000027002.13	28005.151	0.117	0.024	4.915	8.87E-07	2.85E-05	Nckap1	protein_coding
ENSMUSG00000061028.7	4790.275	-0.152	0.031	-4.913	8.97E-07	2.87E-05	Clasrp	protein_coding
ENSMUSG00000001065.15	1672.734	-0.219	0.045	-4.912	9.00E-07	2.88E-05	Zfp276	protein_coding
ENSMUSG00000037475.15	3076.755	0.171	0.035	4.908	9.20E-07	2.93E-05	Thoc2	protein_coding
ENSMUSG00000060601.13	2186.335	-0.210	0.043	-4.906	9.28E-07	2.95E-05	Nr1h2	protein_coding
ENSMUSG00000029570.5	908.815	-0.263	0.054	-4.906	9.29E-07	2.95E-05	Lfng	protein_coding
ENSMUSG00000026211.17	1272.041	-0.211	0.043	-4.903	9.45E-07	2.99E-05	Obsl1	protein_coding
ENSMUSG00000030590.15	1298.243	-0.210	0.043	-4.899	9.63E-07	3.04E-05	Fam98c	protein_coding
ENSMUSG00000073062.3	575.373	0.283	0.058	4.899	9.66E-07	3.04E-05	Zxdb	protein_coding
ENSMUSG00000040938.16	1202.488	-0.239	0.049	-4.894	9.88E-07	3.11E-05	Slc16a11	protein_coding
ENSMUSG00000019894.14	3098.387	0.172	0.035	4.893	9.93E-07	3.12E-05	Slc6a15	protein_coding
ENSMUSG00000021486.6	4014.659	-0.153	0.031	-4.891	1.00E-06	3.14E-05	Prelid1	protein_coding
ENSMUSG00000001802.16	4406.809	-0.192	0.039	-4.887	1.02E-06	3.19E-05	Lrp3	protein_coding
ENSMUSG00000020684.14	11478.503	-0.166	0.034	-4.886	1.03E-06	3.21E-05	Rasl10b	protein_coding
ENSMUSG00000064368.1	1677.830	0.318	0.065	4.882	1.05E-06	3.26E-05	mt-Nd6	protein_coding
ENSMUSG00000047044.7	1084.476	-0.246	0.050	-4.876	1.08E-06	3.36E-05	D030056L22Rik	protein_coding
ENSMUSG00000037416.12	4449.057	0.136	0.028	4.870	1.11E-06	3.45E-05	Dmxl1	protein_coding
ENSMUSG00000043542.12	2796.284	0.174	0.036	4.868	1.13E-06	3.48E-05	Zc2hc1a	protein_coding
ENSMUSG00000048004.15	987.452	0.249	0.051	4.866	1.14E-06	3.51E-05	Tmem196	protein_coding
ENSMUSG00000039830.8	865.981	-0.324	0.067	-4.854	1.21E-06	3.72E-05	Olig2	protein_coding
ENSMUSG00000042423.9	2957.524	-0.203	0.042	-4.853	1.21E-06	3.72E-05	Fbrs	protein_coding
ENSMUSG00000036565.7	14935.223	-0.178	0.037	-4.846	1.26E-06	3.86E-05	Ttyh3	protein_coding
ENSMUSG00000035283.4	934.890	-0.284	0.059	-4.843	1.28E-06	3.90E-05	Adrb1	protein_coding
ENSMUSG00000070436.12	1080.476	-0.319	0.066	-4.842	1.28E-06	3.90E-05	Serpinh1	protein_coding
ENSMUSG00000045180.13	4364.229	0.155	0.032	4.839	1.31E-06	3.97E-05	Shroom2	protein_coding
ENSMUSG00000075700.9	7753.872	0.142	0.029	4.834	1.34E-06	4.06E-05	Selenot	protein_coding
ENSMUSG00000071073.4	1390.579	-0.210	0.044	-4.826	1.40E-06	4.21E-05	Lrrc73	protein_coding
ENSMUSG00000041203.9	1652.704	-0.371	0.077	-4.823	1.41E-06	4.26E-05	2310036O22Rik	protein_coding
ENSMUSG00000026824.11	5376.861	0.141	0.029	4.823	1.42E-06	4.26E-05	Kcnj3	protein_coding

ENSMUSG00000025323.10	1241.555	0.224	0.046	4.822	1.42E-06	4.27E-05	Sp4	protein_coding
ENSMUSG00000023495.13	6842.935	-0.166	0.034	-4.821	1.43E-06	4.28E-05	Pcbp4	protein_coding
ENSMUSG00000021278.7	100.978	-0.375	0.078	-4.816	1.46E-06	4.37E-05	Amn	protein_coding
ENSMUSG00000053310.11	27828.582	-0.232	0.048	-4.811	1.50E-06	4.47E-05	Nrgn	protein_coding
ENSMUSG00000031848.15	694.391	-0.314	0.065	-4.809	1.52E-06	4.51E-05	Lsm4	protein_coding
ENSMUSG00000025982.13	17771.702	0.134	0.028	4.808	1.52E-06	4.51E-05	Sf3b1	protein_coding
ENSMUSG00000017291.13	8501.026	0.125	0.026	4.807	1.54E-06	4.55E-05	Taok1	protein_coding
ENSMUSG00000078578.9	10894.424	0.109	0.023	4.805	1.55E-06	4.57E-05	Ube2d3	protein_coding
ENSMUSG00000031840.12	21304.049	-0.147	0.031	-4.805	1.55E-06	4.57E-05	Rab3a	protein_coding
ENSMUSG00000037400.17	7409.180	0.186	0.039	4.801	1.58E-06	4.64E-05	Atp11b	protein_coding
ENSMUSG00000040681.14	2148.067	-0.196	0.041	-4.797	1.61E-06	4.73E-05	Hmgn1	protein_coding
ENSMUSG00000047143.3	61.797	-0.356	0.074	-4.795	1.63E-06	NA	Dmrta2	protein_coding
ENSMUSG00000007721.6	2184.727	-0.203	0.042	-4.794	1.64E-06	4.80E-05	Ccdc124	protein_coding
ENSMUSG00000022523.9	6306.080	0.162	0.034	4.793	1.65E-06	4.81E-05	Fgf12	protein_coding
ENSMUSG00000024856.10	922.780	-0.236	0.049	-4.787	1.69E-06	4.94E-05	Cdk2ap2	protein_coding
ENSMUSG00000032288.9	645.548	-0.306	0.064	-4.782	1.73E-06	5.04E-05	Imp3	protein_coding
ENSMUSG00000034724.17	2431.909	0.177	0.037	4.782	1.74E-06	5.04E-05	Cnot6l	protein_coding
ENSMUSG00000035390.16	15931.575	-0.159	0.033	-4.782	1.74E-06	5.04E-05	Brsk1	protein_coding
ENSMUSG00000091337.3	8697.179	0.124	0.026	4.777	1.78E-06	5.14E-05	Eid1	protein_coding
ENSMUSG00000048249.14	3025.014	0.172	0.036	4.769	1.85E-06	5.34E-05	Crebrf	protein_coding
ENSMUSG00000025860.14	5249.636	0.145	0.030	4.768	1.86E-06	5.37E-05	Xiap	protein_coding
ENSMUSG00000059208.14	6162.945	-0.144	0.030	-4.760	1.93E-06	5.57E-05	Hnrnpm	protein_coding
ENSMUSG00000000247.11	2006.169	-0.268	0.056	-4.759	1.95E-06	5.59E-05	Lhx2	protein_coding
ENSMUSG00000033763.14	11198.805	-0.179	0.038	-4.755	1.99E-06	5.69E-05	Mtss1l	protein_coding
ENSMUSG00000022791.16	15942.079	-0.168	0.035	-4.752	2.01E-06	5.75E-05	Tnk2	protein_coding
ENSMUSG00000025208.8	1147.573	-0.216	0.046	-4.750	2.03E-06	5.80E-05	Mrpl43	protein_coding
ENSMUSG00000066607.5	6124.985	-0.188	0.039	-4.750	2.04E-06	5.80E-05	6030419C18Rik	protein_coding
ENSMUSG00000026904.17	15224.425	0.137	0.029	4.735	2.19E-06	6.21E-05	Slc4a10	protein_coding
ENSMUSG00000051185.9	1430.540	-0.200	0.042	-4.731	2.23E-06	6.33E-05	Fam174a	protein_coding
ENSMUSG00000019188.16	3920.503	-0.151	0.032	-4.730	2.25E-06	6.37E-05	H13	protein_coding
ENSMUSG00000024750.10	6056.675	0.145	0.031	4.718	2.38E-06	6.71E-05	Zfand5	protein_coding
ENSMUSG00000029840.8	16026.815	0.122	0.026	4.716	2.41E-06	6.79E-05	Mtpn	protein_coding
ENSMUSG00000019877.10	45123.913	0.122	0.026	4.711	2.47E-06	6.94E-05	Serinc1	protein_coding

ENSMUSG00000019790.17	7045.484	0.145	0.031	4.707	2.51E-06	7.05E-05	Stxbp5	protein_coding
ENSMUSG00000059742.10	5538.595	0.186	0.040	4.706	2.53E-06	7.09E-05	Kcnh7	protein_coding
ENSMUSG00000026153.15	1816.296	0.177	0.038	4.705	2.54E-06	7.11E-05	Fam135a	protein_coding
ENSMUSG00000025245.14	3365.111	0.144	0.031	4.700	2.60E-06	7.25E-05	Lztfl1	protein_coding
ENSMUSG00000033917.15	8206.649	-0.137	0.029	-4.696	2.65E-06	7.38E-05	Gde1	protein_coding
ENSMUSG00000020664.10	5140.353	0.128	0.027	4.695	2.66E-06	7.40E-05	Dld	protein_coding
ENSMUSG00000027799.12	17367.439	0.107	0.023	4.693	2.70E-06	7.46E-05	Nbea	protein_coding
ENSMUSG00000074247.10	3266.009	-0.170	0.036	-4.692	2.71E-06	7.49E-05	Dda1	protein_coding
ENSMUSG00000019158.9	510.387	-0.331	0.071	-4.684	2.81E-06	7.76E-05	Tmem160	protein_coding
ENSMUSG00000025656.17	22196.141	0.112	0.024	4.683	2.83E-06	7.78E-05	Arhgef9	protein_coding
ENSMUSG00000004364.14	6524.337	0.124	0.027	4.681	2.85E-06	7.83E-05	Cul3	protein_coding
ENSMUSG00000030869.11	1832.502	-0.196	0.042	-4.671	3.00E-06	8.23E-05	Ndufab1	protein_coding
ENSMUSG00000078348.4	876.837	-0.245	0.052	-4.663	3.11E-06	8.51E-05	Sf3b5	protein_coding
ENSMUSG00000090100.7	4611.823	0.143	0.031	4.663	3.11E-06	8.51E-05	Ttbk2	protein_coding
ENSMUSG00000045045.7	1931.610	-0.229	0.049	-4.659	3.18E-06	8.68E-05	Lrfn4	protein_coding
ENSMUSG00000001833.17	20175.465	0.161	0.035	4.657	3.20E-06	8.71E-05	Sept7	protein_coding
ENSMUSG00000016356.17	325.596	-0.323	0.069	-4.652	3.29E-06	8.93E-05	Col20a1	protein_coding
ENSMUSG00000045252.11	1473.132	-0.202	0.043	-4.650	3.31E-06	8.98E-05	Zfp574	protein_coding
ENSMUSG00000045948.8	1289.180	-0.223	0.048	-4.649	3.34E-06	9.04E-05	Mrps12	protein_coding
ENSMUSG00000020385.16	4178.366	0.144	0.031	4.648	3.36E-06	9.07E-05	Clk4	protein_coding
ENSMUSG00000047264.8	1198.834	-0.257	0.055	-4.644	3.41E-06	9.19E-05	Zfp358	protein_coding
ENSMUSG00000001240.13	470.613	-0.321	0.069	-4.643	3.43E-06	9.22E-05	Ramp2	protein_coding
ENSMUSG00000024940.10	1955.799	-0.229	0.049	-4.643	3.44E-06	9.24E-05	Ltbp3	protein_coding
ENSMUSG00000006262.15	1634.485	0.204	0.044	4.642	3.45E-06	9.25E-05	Mob1b	protein_coding
ENSMUSG00000037703.14	14556.195	-0.122	0.026	-4.641	3.47E-06	9.29E-05	Lzts3	protein_coding
ENSMUSG00000036902.11	3218.860	0.156	0.034	4.633	3.61E-06	9.63E-05	Neto2	protein_coding
ENSMUSG00000020230.15	3639.145	-0.149	0.032	-4.628	3.69E-06	9.83E-05	Prmt2	protein_coding
ENSMUSG00000038894.7	5506.232	-0.203	0.044	-4.625	3.75E-06	9.96E-05	Irs2	protein_coding
ENSMUSG00000035711.4	819.414	-0.272	0.059	-4.624	3.77E-06	0.000100076	Dok3	protein_coding
ENSMUSG00000047260.4	1015.646	-0.262	0.057	-4.620	3.83E-06	0.000101432	Emc6	protein_coding
ENSMUSG00000031601.16	4603.816	0.149	0.032	4.618	3.87E-06	0.000102354	Cnot7	protein_coding
ENSMUSG00000030795.18	16057.247	0.116	0.025	4.615	3.93E-06	0.000103736	Fus	protein_coding
ENSMUSG00000031532.6	9864.035	-0.114	0.025	-4.612	3.98E-06	0.000104878	Saraf	protein_coding

ENSMUSG00000047547.14	6542.985	-0.144	0.031	-4.610	4.03E-06	0.000105843	Cltb	protein_coding
ENSMUSG00000026568.6	1975.408	-0.204	0.044	-4.609	4.05E-06	0.000106264	Mpc2	protein_coding
ENSMUSG00000054863.8	4024.588	-0.158	0.034	-4.608	4.06E-06	0.000106264	Fam19a5	protein_coding
ENSMUSG00000020516.15	2835.399	0.188	0.041	4.605	4.13E-06	0.000108077	Rps6kb1	protein_coding
ENSMUSG00000031808.7	5653.677	-0.173	0.038	-4.604	4.15E-06	0.000108224	Slc27a1	protein_coding
ENSMUSG00000033545.14	7345.622	-0.159	0.034	-4.603	4.17E-06	0.000108534	Znrf1	protein_coding
ENSMUSG00000020519.5	636.117	-0.268	0.058	-4.602	4.19E-06	0.000108815	Sap30l	protein_coding
ENSMUSG00000006731.10	6768.022	-0.177	0.038	-4.602	4.19E-06	0.000108815	B4galnt1	protein_coding
ENSMUSG00000007603.8	4004.766	-0.167	0.036	-4.600	4.22E-06	0.000109398	Dus3l	protein_coding
ENSMUSG00000027770.5	4331.871	0.154	0.033	4.599	4.24E-06	0.000109727	Dhx36	protein_coding
ENSMUSG00000029836.15	1422.449	0.222	0.048	4.590	4.43E-06	0.000114375	Cbx3	protein_coding
ENSMUSG00000044496.6	1250.320	-0.210	0.046	-4.585	4.54E-06	0.000116849	2510039O18Rik	protein_coding
ENSMUSG00000084883.2	3214.214	-0.154	0.034	-4.584	4.56E-06	0.000117123	Ccdc85c	protein_coding
ENSMUSG00000007029.16	2620.861	-0.177	0.039	-4.583	4.58E-06	0.000117302	Vars	protein_coding
ENSMUSG00000027834.15	15446.353	0.176	0.038	4.580	4.66E-06	0.000119075	Serpini1	protein_coding
ENSMUSG00000021072.12	1862.858	0.176	0.038	4.579	4.67E-06	0.000119333	Tmx1	protein_coding
ENSMUSG00000026207.16	9317.618	-0.198	0.043	-4.577	4.71E-06	0.00012008	Speg	protein_coding
ENSMUSG00000038517.15	3557.348	-0.190	0.042	-4.577	4.72E-06	0.00012008	Tbkbp1	protein_coding
ENSMUSG00000050029.7	1637.095	0.185	0.040	4.575	4.77E-06	0.000121114	Rap2c	protein_coding
ENSMUSG00000095407.1	600.076	-0.295	0.064	-4.572	4.82E-06	0.000122283	Tmem200c	protein_coding
ENSMUSG00000062257.13	10301.174	0.111	0.024	4.571	4.86E-06	0.000123076	Opcml	protein_coding
ENSMUSG00000030265.14	5480.667	0.134	0.030	4.556	5.21E-06	0.000131681	Kras	protein_coding
ENSMUSG00000013833.15	2838.964	-0.201	0.044	-4.552	5.30E-06	0.000133685	Med16	protein_coding
ENSMUSG00000036894.3	2226.362	-0.161	0.035	-4.552	5.32E-06	0.00013394	Rap2b	protein_coding
ENSMUSG00000058239.13	2221.399	-0.230	0.050	-4.551	5.33E-06	0.00013394	Usf2	protein_coding
ENSMUSG00000048696.11	665.995	-0.311	0.068	-4.548	5.41E-06	0.000135744	Mex3d	protein_coding
ENSMUSG00000029455.14	3876.341	-0.181	0.040	-4.547	5.43E-06	0.000135908	Aldh2	protein_coding
ENSMUSG00000028020.16	8391.639	0.125	0.027	4.546	5.47E-06	0.000136763	Glrb	protein_coding
ENSMUSG00000022674.14	1482.532	0.216	0.047	4.545	5.49E-06	0.000136928	Ube2v2	protein_coding
ENSMUSG00000041995.8	373.668	-0.286	0.063	-4.545	5.50E-06	0.000136938	Zbed3	protein_coding
ENSMUSG00000027499.12	8780.793	0.149	0.033	4.540	5.61E-06	0.000139523	Pkia	protein_coding
ENSMUSG00000071862.2	4644.870	0.188	0.041	4.539	5.65E-06	0.000140139	Lrrtm2	protein_coding
ENSMUSG00000045009.9	3353.794	-0.196	0.043	-4.537	5.70E-06	0.000141146	Prrt3	protein_coding

ENSMUSG00000043004.13	9114.897	0.143	0.031	4.530	5.89E-06	0.000145436	Gng2	protein_coding
ENSMUSG00000037373.12	7825.328	-0.121	0.027	-4.530	5.89E-06	0.000145436	Ctbp1	protein_coding
ENSMUSG00000038630.8	2580.223	0.187	0.041	4.524	6.06E-06	0.000149124	Zkscan16	protein_coding
ENSMUSG00000020484.18	5645.125	-0.138	0.030	-4.523	6.10E-06	0.000149888	Xbp1	protein_coding
ENSMUSG00000023020.2	1406.775	-0.209	0.046	-4.520	6.17E-06	0.000151298	Cox14	protein_coding
ENSMUSG00000024083.15	31156.328	0.121	0.027	4.518	6.24E-06	0.00015261	Pja2	protein_coding
ENSMUSG00000048047.3	1736.097	0.171	0.038	4.518	6.26E-06	0.000152787	Zbtb33	protein_coding
ENSMUSG00000043496.7	1953.831	-0.179	0.040	-4.516	6.31E-06	0.000153754	Tril	protein_coding
ENSMUSG00000005506.16	13353.198	0.095	0.021	4.514	6.36E-06	0.000154784	Celf1	protein_coding
ENSMUSG00000028546.17	5077.304	0.159	0.035	4.514	6.37E-06	0.000154784	Elavl4	protein_coding
ENSMUSG00000020863.15	10148.131	0.107	0.024	4.513	6.40E-06	0.000155242	Luc7l3	protein_coding
ENSMUSG00000021218.10	10278.274	0.109	0.024	4.512	6.43E-06	0.000155755	Gdi2	protein_coding
ENSMUSG00000033434.15	1421.061	-0.223	0.049	-4.511	6.46E-06	0.00015628	Gtpbp6	protein_coding
ENSMUSG00000034560.6	2558.676	0.154	0.034	4.505	6.62E-06	0.000159685	Washc4	protein_coding
ENSMUSG00000023909.3	2443.085	-0.173	0.038	-4.505	6.63E-06	0.000159685	Paqr4	protein_coding
ENSMUSG00000056895.4	321.522	-0.317	0.070	-4.504	6.68E-06	0.000160654	Hist3h2ba	protein_coding
ENSMUSG00000022533.13	4202.503	0.133	0.030	4.501	6.77E-06	0.000162515	Atp13a3	protein_coding
ENSMUSG00000068859.5	311.802	-0.342	0.076	-4.500	6.78E-06	0.000162622	Sp9	protein_coding
ENSMUSG00000035277.15	895.639	-0.264	0.059	-4.499	6.84E-06	0.000163641	Arx	protein_coding
ENSMUSG00000037706.17	9050.516	-0.134	0.030	-4.498	6.86E-06	0.000163987	Cd81	protein_coding
ENSMUSG00000035847.15	29924.472	0.143	0.032	4.497	6.89E-06	0.000164307	Ids	protein_coding
ENSMUSG00000033697.15	5123.079	-0.151	0.034	-4.496	6.93E-06	0.000164938	Arhgap39	protein_coding
ENSMUSG00000030428.16	24779.030	-0.111	0.025	-4.495	6.96E-06	0.00016538	Ttyh1	protein_coding
ENSMUSG00000022016.15	15407.076	0.116	0.026	4.491	7.08E-06	0.000168078	Akap11	protein_coding
ENSMUSG00000021929.8	4476.846	0.131	0.029	4.488	7.20E-06	0.000170433	Kpna3	protein_coding
ENSMUSG00000022055.7	17096.884	-0.135	0.030	-4.487	7.21E-06	0.000170433	Nefl	protein_coding
ENSMUSG00000024480.7	1666.321	0.175	0.039	4.486	7.27E-06	0.000171703	Ap3s1	protein_coding
ENSMUSG00000020198.8	12550.656	-0.109	0.024	-4.479	7.51E-06	0.000177063	Ap3d1	protein_coding
ENSMUSG00000021986.7	1210.314	-0.221	0.049	-4.463	8.09E-06	0.000190245	Amer2	protein_coding
ENSMUSG00000060860.8	716.132	-0.237	0.053	-4.460	8.19E-06	0.000192468	Ube2s	protein_coding
ENSMUSG00000055373.8	8076.292	0.205	0.046	4.456	8.36E-06	0.000196117	Fut9	protein_coding
ENSMUSG00000026721.15	7315.541	0.153	0.034	4.453	8.48E-06	0.000198542	Rabgap1l	protein_coding
ENSMUSG00000039753.16	5065.642	0.163	0.037	4.450	8.59E-06	0.000200829	Fbxl5	protein_coding

ENSMUSG00000003863.18	9987.005	-0.154	0.035	-4.447	8.70E-06	0.000203089	Ppfia3	protein_coding
ENSMUSG00000046546.3	627.519	-0.283	0.064	-4.445	8.78E-06	0.000204521	Fam43a	protein_coding
ENSMUSG00000025964.15	6403.633	0.116	0.026	4.444	8.81E-06	0.000204922	Adam23	protein_coding
ENSMUSG00000021448.7	2913.272	-0.205	0.046	-4.444	8.83E-06	0.000204922	Shc3	protein_coding
ENSMUSG00000013736.16	1778.055	0.202	0.045	4.444	8.84E-06	0.000204922	Trnt1	protein_coding
ENSMUSG00000050212.4	51.204	-0.331	0.075	-4.438	9.09E-06	NA	Eva1b	protein_coding
ENSMUSG00000029571.14	8178.343	0.140	0.032	4.437	9.11E-06	0.000210555	Tmem106b	protein_coding
ENSMUSG00000028248.15	15642.569	0.120	0.027	4.437	9.11E-06	0.000210555	Pnisr	protein_coding
ENSMUSG00000033597.8	11960.629	-0.183	0.041	-4.435	9.21E-06	0.000212492	Caskin1	protein_coding
ENSMUSG00000028861.13	1115.207	-0.200	0.045	-4.429	9.46E-06	0.000217932	Mrps15	protein_coding
ENSMUSG00000031770.16	2880.839	-0.164	0.037	-4.428	9.49E-06	0.000218217	Herpud1	protein_coding
ENSMUSG00000031201.17	1463.773	0.204	0.046	4.425	9.62E-06	0.00022056	Brcc3	protein_coding
ENSMUSG00000067194.6	7792.697	0.125	0.028	4.423	9.75E-06	0.00022317	Eif1ax	protein_coding
ENSMUSG00000003948.17	7125.403	0.149	0.034	4.422	9.78E-06	0.000223459	Mmd	protein_coding
ENSMUSG00000030019.6	1165.017	-0.222	0.050	-4.421	9.82E-06	0.000223954	Fbxl14	protein_coding
ENSMUSG00000034993.7	2137.182	-0.246	0.056	-4.417	1.00E-05	0.000227796	Vat1	protein_coding
ENSMUSG00000000581.8	1412.740	0.187	0.042	4.415	1.01E-05	0.000228819	C1d	protein_coding
ENSMUSG00000034209.4	1747.091	-0.216	0.049	-4.415	1.01E-05	0.000229164	Rasl10a	protein_coding
ENSMUSG00000045427.13	5664.287	0.113	0.026	4.413	1.02E-05	0.000230862	Hnrnph2	protein_coding
ENSMUSG00000029804.16	10451.836	0.117	0.027	4.411	1.03E-05	0.000232308	Herc3	protein_coding
ENSMUSG00000034818.16	18439.030	-0.122	0.028	-4.407	1.05E-05	0.000236272	Celf5	protein_coding
ENSMUSG00000006476.19	26311.380	-0.148	0.034	-4.405	1.06E-05	0.000237847	Nsmf	protein_coding
ENSMUSG00000029599.13	2489.169	-0.156	0.035	-4.401	1.08E-05	0.000242271	Ddx54	protein_coding
ENSMUSG00000020018.6	472.875	-0.280	0.064	-4.392	1.12E-05	0.000251563	Snrpf	protein_coding
ENSMUSG00000034799.16	28898.141	-0.137	0.031	-4.392	1.12E-05	0.000251563	Unc13a	protein_coding
ENSMUSG00000022905.12	7678.400	0.117	0.027	4.392	1.12E-05	0.000251563	Kpna1	protein_coding
ENSMUSG00000037907.16	5736.492	-0.176	0.040	-4.390	1.14E-05	0.000253539	Ankrd13b	protein_coding
ENSMUSG00000013662.5	4588.092	0.130	0.030	4.384	1.17E-05	0.000259846	Atad1	protein_coding
ENSMUSG00000035864.14	81951.713	0.086	0.020	4.382	1.18E-05	0.000262211	Syt1	protein_coding
ENSMUSG00000031327.10	2238.036	0.178	0.041	4.381	1.18E-05	0.000262211	Chic1	protein_coding
ENSMUSG00000020153.14	2840.089	-0.166	0.038	-4.380	1.19E-05	0.00026326	Ndufs7	protein_coding
ENSMUSG00000034390.16	10701.327	-0.136	0.031	-4.380	1.19E-05	0.00026326	Cmip	protein_coding
ENSMUSG00000042742.7	1676.939	0.181	0.041	4.369	1.25E-05	0.000276209	Bmt2	protein_coding

ENSMUSG00000069565.12	1852.233	-0.192	0.044	-4.368	1.25E-05	0.000276593	Dazap1	protein_coding
ENSMUSG00000069662.5	5724.284	0.148	0.034	4.368	1.25E-05	0.000276641	Marcks	protein_coding
ENSMUSG00000036873.13	965.281	-0.222	0.051	-4.367	1.26E-05	0.000277121	2410004B18Rik	protein_coding
ENSMUSG00000052837.6	2384.262	-0.331	0.076	-4.366	1.27E-05	0.000278709	Junb	protein_coding
ENSMUSG00000022788.16	1769.344	0.183	0.042	4.365	1.27E-05	0.000278945	Fgd4	protein_coding
ENSMUSG00000030122.12	13277.412	-0.141	0.032	-4.364	1.28E-05	0.000280127	Ptms	protein_coding
ENSMUSG00000028458.12	3921.537	-0.176	0.040	-4.363	1.28E-05	0.000280127	Tesk1	protein_coding
ENSMUSG00000062014.12	10151.507	0.126	0.029	4.363	1.28E-05	0.000280731	Gmfb	protein_coding
ENSMUSG00000052759.7	102.270	-0.340	0.078	-4.361	1.29E-05	0.000282183	Gpr25	protein_coding
ENSMUSG00000020189.14	6913.507	0.154	0.035	4.359	1.30E-05	0.000283992	Osbpl8	protein_coding
ENSMUSG00000020311.17	2031.130	0.172	0.039	4.358	1.31E-05	0.000285575	Erlec1	protein_coding
ENSMUSG00000025854.15	1644.819	-0.209	0.048	-4.357	1.32E-05	0.000286563	Fam20c	protein_coding
ENSMUSG00000037990.18	2964.628	-0.151	0.035	-4.350	1.36E-05	0.000295356	Sh3rf3	protein_coding
ENSMUSG00000020733.3	1627.682	-0.179	0.041	-4.347	1.38E-05	0.000298669	Slc9a3r1	protein_coding
ENSMUSG00000027895.9	4259.428	-0.163	0.037	-4.345	1.40E-05	0.000301629	Kcnc4	protein_coding
ENSMUSG00000061518.10	4540.822	-0.176	0.040	-4.342	1.41E-05	0.000304655	Cox5b	protein_coding
ENSMUSG00000003068.16	2864.272	-0.155	0.036	-4.341	1.42E-05	0.000306021	Stk11	protein_coding
ENSMUSG00000020644.9	2510.015	0.168	0.039	4.340	1.43E-05	0.000306748	Id2	protein_coding
ENSMUSG00000019370.10	48977.554	-0.110	0.025	-4.339	1.43E-05	0.000307524	Calm3	protein_coding
ENSMUSG00000056076.13	4572.935	-0.126	0.029	-4.333	1.47E-05	0.000315347	Eif3b	protein_coding
ENSMUSG00000075318.12	20326.797	0.113	0.026	4.327	1.51E-05	0.000323474	Scn2a	protein_coding
ENSMUSG00000032598.8	4467.029	-0.148	0.034	-4.326	1.52E-05	0.000325315	Nckipsd	protein_coding
ENSMUSG00000022018.7	400.258	-0.314	0.073	-4.325	1.53E-05	0.000325993	Rgcc	protein_coding
ENSMUSG00000019804.12	4526.883	-0.173	0.040	-4.322	1.55E-05	0.000330308	Snx3	protein_coding
ENSMUSG00000037492.16	2313.850	0.162	0.037	4.321	1.55E-05	0.000330308	Zmat4	protein_coding
ENSMUSG00000039478.15	4192.281	0.132	0.031	4.319	1.57E-05	0.000333283	Micu3	protein_coding
ENSMUSG00000003308.15	3154.709	-0.158	0.036	-4.318	1.57E-05	0.000333553	Keap1	protein_coding
ENSMUSG00000021743.5	2664.576	-0.207	0.048	-4.313	1.61E-05	0.000341381	Fezf2	protein_coding
ENSMUSG00000029211.11	4683.663	0.179	0.042	4.308	1.65E-05	0.000349022	Gabra4	protein_coding
ENSMUSG00000031988.10	6278.764	-0.112	0.026	-4.304	1.68E-05	0.000353949	Vps26b	protein_coding
ENSMUSG00000003199.16	3136.118	-0.154	0.036	-4.302	1.69E-05	0.000357183	Mpnd	protein_coding
ENSMUSG00000017548.15	2011.574	0.160	0.037	4.300	1.70E-05	0.000359017	Suz12	protein_coding
ENSMUSG00000024245.4	3806.038	-0.163	0.038	-4.299	1.72E-05	0.000361009	Tmem178	protein_coding

ENSMUSG00000037014.4	1005.966	-0.231	0.054	-4.296	1.74E-05	0.000364634	Sstr4	protein_coding
ENSMUSG00000049097.9	3011.872	-0.191	0.044	-4.294	1.75E-05	0.000367564	Ankrd34a	protein_coding
ENSMUSG00000038172.14	4929.237	0.129	0.030	4.294	1.76E-05	0.000367704	Ttc39b	protein_coding
ENSMUSG00000029060.17	7297.864	-0.182	0.042	-4.293	1.77E-05	0.000369177	Mib2	protein_coding
ENSMUSG00000047036.8	12837.570	0.121	0.028	4.291	1.78E-05	0.000371372	Zfp445	protein_coding
ENSMUSG00000015937.14	3022.496	-0.149	0.035	-4.288	1.80E-05	0.000376126	H2afy	protein_coding
ENSMUSG00000011257.19	1585.018	-0.198	0.046	-4.287	1.81E-05	0.000377661	Pabpc4	protein_coding
ENSMUSG00000052957.7	260.629	-0.309	0.072	-4.284	1.83E-05	0.000381259	Gas1	protein_coding
ENSMUSG00000049265.7	1449.372	-0.206	0.048	-4.284	1.84E-05	0.000381862	Kcnk3	protein_coding
ENSMUSG00000039041.15	2054.062	-0.163	0.038	-4.283	1.84E-05	0.000381933	Adrm1	protein_coding
ENSMUSG00000037933.16	6487.731	-0.129	0.030	-4.282	1.85E-05	0.000383869	Bicd2	protein_coding
ENSMUSG00000024966.8	8582.309	-0.153	0.036	-4.279	1.88E-05	0.000388025	Stip1	protein_coding
ENSMUSG00000020388.12	336.076	-0.279	0.065	-4.278	1.88E-05	0.000388724	Pdlim4	protein_coding
ENSMUSG00000020620.14	1924.488	0.159	0.037	4.277	1.89E-05	0.000390028	Abca8b	protein_coding
ENSMUSG00000030087.11	1344.089	-0.193	0.045	-4.276	1.91E-05	0.000392009	Klf15	protein_coding
ENSMUSG00000031342.17	39572.714	0.119	0.028	4.269	1.96E-05	0.00040268	Gpm6b	protein_coding
ENSMUSG00000013236.16	27146.871	-0.134	0.031	-4.267	1.98E-05	0.000406718	Ptprs	protein_coding
ENSMUSG00000029413.14	1390.187	-0.185	0.043	-4.265	2.00E-05	0.000408877	Naaa	protein_coding
ENSMUSG00000038128.6	8906.811	0.150	0.035	4.262	2.02E-05	0.000413875	Camk4	protein_coding
ENSMUSG00000020235.16	3274.937	-0.141	0.033	-4.257	2.07E-05	0.000423816	Fzr1	protein_coding
ENSMUSG00000032589.14	40459.199	-0.155	0.037	-4.252	2.12E-05	0.00043149	Bsn	protein_coding
ENSMUSG00000055296.14	6748.077	0.121	0.028	4.251	2.13E-05	0.00043329	Tmem245	protein_coding
ENSMUSG00000002771.12	1042.647	-0.250	0.059	-4.246	2.17E-05	0.000441663	Grin2d	protein_coding
ENSMUSG00000032239.10	892.081	-0.241	0.057	-4.244	2.20E-05	0.000446137	Rp9	protein_coding
ENSMUSG00000026959.13	29292.710	-0.125	0.030	-4.243	2.21E-05	0.000447187	Grin1	protein_coding
ENSMUSG00000094870.7	2011.578	0.175	0.041	4.242	2.21E-05	0.000447762	Zfp131	protein_coding
ENSMUSG00000062563.15	988.821	-0.204	0.048	-4.240	2.23E-05	0.000451897	Cys1	protein_coding
ENSMUSG00000032293.8	3859.027	0.137	0.032	4.236	2.28E-05	0.000459809	Ireb2	protein_coding
ENSMUSG00000059824.12	2799.446	-0.183	0.043	-4.235	2.28E-05	0.000459809	Dbp	protein_coding
ENSMUSG00000000078.7	2735.456	0.147	0.035	4.232	2.32E-05	0.00046661	Klf6	protein_coding
ENSMUSG00000047126.17	34456.705	0.106	0.025	4.231	2.32E-05	0.000466772	Cltc	protein_coding
ENSMUSG00000024261.5	12393.616	0.119	0.028	4.226	2.38E-05	0.000477297	Syt4	protein_coding
ENSMUSG00000004934.14	1057.440	-0.221	0.052	-4.224	2.40E-05	0.000480258	Pias4	protein_coding

ENSMUSG00000017221.14	5706.538	-0.114	0.027	-4.218	2.46E-05	0.00049307	Psmd3	protein_coding
ENSMUSG00000060275.11	388.874	-0.273	0.065	-4.211	2.54E-05	0.000507677	Nrg2	protein_coding
ENSMUSG00000030409.15	1512.995	-0.217	0.052	-4.209	2.57E-05	0.000512652	Dmpk	protein_coding
ENSMUSG00000024286.7	5882.664	0.131	0.031	4.200	2.67E-05	0.000532722	Ccny	protein_coding
ENSMUSG00000074358.9	392.893	-0.277	0.066	-4.199	2.68E-05	0.000533091	Ccdc61	protein_coding
ENSMUSG00000045103.17	4424.498	0.119	0.028	4.199	2.68E-05	0.000533091	Dmd	protein_coding
ENSMUSG00000036138.16	3507.773	-0.158	0.038	-4.198	2.69E-05	0.000533884	Acaa1a	protein_coding
ENSMUSG00000031239.5	1736.406	0.177	0.042	4.192	2.76E-05	0.000547255	Itm2a	protein_coding
ENSMUSG00000003226.7	9429.758	0.112	0.027	4.190	2.79E-05	0.000551715	Ranbp2	protein_coding
ENSMUSG00000053716.9	3136.885	-0.175	0.042	-4.187	2.83E-05	0.00055887	Dusp7	protein_coding
ENSMUSG00000032297.11	1513.449	-0.225	0.054	-4.186	2.84E-05	0.000559749	Celf6	protein_coding
ENSMUSG00000040957.14	1077.296	-0.225	0.054	-4.186	2.84E-05	0.000559749	Cables1	protein_coding
ENSMUSG00000020135.13	11839.130	-0.160	0.038	-4.185	2.86E-05	0.00056206	Apc2	protein_coding
ENSMUSG00000034880.7	466.094	-0.280	0.067	-4.177	2.95E-05	0.000580158	Mrpl34	protein_coding
ENSMUSG00000018800.14	3119.502	0.142	0.034	4.177	2.96E-05	0.000580158	Abca5	protein_coding
ENSMUSG00000051495.7	1462.992	-0.191	0.046	-4.176	2.96E-05	0.000580562	Irf2bp2	protein_coding
ENSMUSG00000024212.8	4763.208	-0.133	0.032	-4.175	2.98E-05	0.000581535	Mllt1	protein_coding
ENSMUSG00000026484.13	1334.780	0.196	0.047	4.175	2.98E-05	0.000581535	Rnf2	protein_coding
ENSMUSG00000003528.14	1484.074	-0.192	0.046	-4.175	2.98E-05	0.000581535	Slc25a1	protein_coding
ENSMUSG00000031242.7	3444.417	0.155	0.037	4.173	3.00E-05	0.000584929	2610002M06Rik	protein_coding
ENSMUSG00000017412.15	12016.001	0.146	0.035	4.169	3.06E-05	0.000595306	Cacnb4	protein_coding
ENSMUSG00000028524.21	9249.259	0.096	0.023	4.169	3.06E-05	0.000595306	Sgip1	protein_coding
ENSMUSG00000078570.10	939.177	-0.225	0.054	-4.168	3.07E-05	0.000596795	1110065P20Rik	protein_coding
ENSMUSG00000003345.16	6717.407	-0.150	0.036	-4.167	3.09E-05	0.000598887	Csnk1g2	protein_coding
ENSMUSG00000060279.14	15957.828	-0.124	0.030	-4.163	3.14E-05	0.000607212	Ap2a1	protein_coding
ENSMUSG00000000326.12	3676.874	-0.126	0.030	-4.159	3.20E-05	0.00061871	Comt	protein_coding
ENSMUSG00000014782.16	457.830	-0.276	0.066	-4.154	3.26E-05	0.0006297	Plekhg4	protein_coding
ENSMUSG00000028572.13	5780.891	0.122	0.029	4.154	3.27E-05	0.000630571	Hook1	protein_coding
ENSMUSG00000022789.14	12472.224	0.096	0.023	4.153	3.27E-05	0.000630571	Dnm1l	protein_coding
ENSMUSG00000028212.16	227.726	0.303	0.073	4.152	3.29E-05	0.000632572	Ccne2	protein_coding
ENSMUSG00000036948.17	3214.988	-0.181	0.044	-4.152	3.29E-05	0.000632572	BC037034	protein_coding
ENSMUSG00000041351.16	16365.580	-0.133	0.032	-4.150	3.33E-05	0.000638546	Rap1gap	protein_coding
ENSMUSG00000027109.16	2620.821	0.175	0.042	4.149	3.34E-05	0.000639651	Sp3	protein_coding

ENSMUSG00000047181.12	2922.636	-0.213	0.051	-4.147	3.36E-05	0.00064256	Samd14	protein_coding
ENSMUSG00000012422.14	2625.639	0.145	0.035	4.147	3.36E-05	0.00064256	Tmem167	protein_coding
ENSMUSG00000007812.16	1771.830	0.157	0.038	4.144	3.41E-05	0.000649331	Zfp655	protein_coding
ENSMUSG00000006676.16	11511.042	-0.123	0.030	-4.141	3.46E-05	0.00065818	Usp19	protein_coding
ENSMUSG00000019428.16	14967.886	-0.153	0.037	-4.135	3.56E-05	0.000675418	Fkbp8	protein_coding
ENSMUSG00000052917.14	2071.501	0.173	0.042	4.131	3.61E-05	0.000683845	Senp7	protein_coding
ENSMUSG00000046791.8	664.713	-0.219	0.053	-4.126	3.70E-05	0.000700254	Riox1	protein_coding
ENSMUSG00000035203.16	18960.868	-0.193	0.047	-4.125	3.71E-05	0.000701119	Epn1	protein_coding
ENSMUSG00000052133.16	2091.271	-0.165	0.040	-4.124	3.72E-05	0.000702311	Sema5b	protein_coding
ENSMUSG00000036815.16	8615.556	0.108	0.026	4.124	3.73E-05	0.000704015	Dpp10	protein_coding
ENSMUSG00000020859.16	21485.161	0.085	0.021	4.120	3.79E-05	0.000713206	Spag9	protein_coding
ENSMUSG00000040640.10	8036.130	0.137	0.033	4.118	3.82E-05	0.000718337	Erc2	protein_coding
ENSMUSG00000031167.16	3869.963	0.142	0.035	4.118	3.82E-05	0.000718337	Rbm3	protein_coding
ENSMUSG00000002968.8	4758.469	-0.166	0.040	-4.116	3.85E-05	0.000721817	Med25	protein_coding
ENSMUSG00000062078.14	13028.032	0.169	0.041	4.116	3.85E-05	0.000721817	Qk	protein_coding
ENSMUSG00000008489.18	4231.942	0.152	0.037	4.116	3.86E-05	0.000721817	Elavl2	protein_coding
ENSMUSG00000001986.16	15880.195	0.125	0.030	4.109	3.98E-05	0.000743749	Gria3	protein_coding
ENSMUSG00000000531.4	2049.137	-0.214	0.052	-4.108	3.99E-05	0.000743749	Grasp	protein_coding
ENSMUSG00000022800.13	4196.382	0.122	0.030	4.108	3.99E-05	0.000743749	Fyttd1	protein_coding
ENSMUSG00000031360.14	2461.223	0.152	0.037	4.108	3.99E-05	0.000743749	Ctps2	protein_coding
ENSMUSG00000040760.10	6638.965	0.107	0.026	4.107	4.02E-05	0.000744945	Appl1	protein_coding
ENSMUSG00000059674.6	837.640	-0.247	0.060	-4.107	4.02E-05	0.000744945	Cdh24	protein_coding
ENSMUSG00000040563.13	9375.418	-0.178	0.043	-4.106	4.02E-05	0.000744945	Plppr2	protein_coding
ENSMUSG00000021266.16	4587.164	-0.120	0.029	-4.102	4.09E-05	0.000757728	Wars	protein_coding
ENSMUSG00000039770.5	3249.533	0.143	0.035	4.098	4.17E-05	0.000770811	Ypel5	protein_coding
ENSMUSG00000033943.15	5184.026	0.119	0.029	4.097	4.19E-05	0.000774115	Mga	protein_coding
ENSMUSG00000031644.19	3130.640	0.137	0.033	4.094	4.24E-05	0.000781971	Nek1	protein_coding
ENSMUSG00000021484.7	4572.064	-0.127	0.031	-4.093	4.26E-05	0.000785302	Lman2	protein_coding
ENSMUSG00000059142.15	975.835	0.215	0.052	4.092	4.29E-05	0.000788438	Zfp945	protein_coding
ENSMUSG00000032336.17	24772.810	0.121	0.030	4.089	4.33E-05	0.000795748	Nptn	protein_coding
ENSMUSG00000005148.7	383.648	-0.269	0.066	-4.086	4.38E-05	0.000804206	Klf5	protein_coding
ENSMUSG00000042133.16	3739.601	0.134	0.033	4.086	4.40E-05	0.000805013	Ppig	protein_coding
ENSMUSG00000027365.14	3378.412	0.154	0.038	4.086	4.40E-05	0.000805013	Trpm7	protein_coding

ENSMUSG00000082229.1	5269.514	0.152	0.037	4.082	4.47E-05	0.000817958	Nap1l2	protein_coding
ENSMUSG00000029253.12	1289.513	0.187	0.046	4.075	4.61E-05	0.000841475	Cenpc1	protein_coding
ENSMUSG00000032253.13	3692.887	0.138	0.034	4.074	4.62E-05	0.00084326	Phip	protein_coding
ENSMUSG00000072969.2	983.647	0.206	0.050	4.073	4.64E-05	0.00084425	Armcx5	protein_coding
ENSMUSG00000071074.9	1986.061	-0.154	0.038	-4.071	4.68E-05	0.000850285	Yipf3	protein_coding
ENSMUSG00000092083.4	1386.745	0.232	0.057	4.070	4.69E-05	0.000852111	Kcnb2	protein_coding
ENSMUSG00000026520.8	1090.736	-0.197	0.049	-4.069	4.72E-05	0.000855596	Pycr2	protein_coding
ENSMUSG00000024097.10	5477.947	0.118	0.029	4.067	4.76E-05	0.000861954	Srsf7	protein_coding
ENSMUSG00000028743.7	805.055	-0.237	0.058	-4.066	4.78E-05	0.000865547	Akr7a5	protein_coding
ENSMUSG00000062373.7	3804.516	0.139	0.034	4.065	4.81E-05	0.00086844	Tmem65	protein_coding
ENSMUSG00000034889.8	1231.390	-0.203	0.050	-4.063	4.84E-05	0.000873683	Cactin	protein_coding
ENSMUSG00000074649.6	2536.414	-0.168	0.041	-4.061	4.88E-05	0.000879005	BC029722	protein_coding
ENSMUSG00000030465.19	35699.300	0.112	0.028	4.058	4.94E-05	0.000888505	Psd3	protein_coding
ENSMUSG00000031788.14	2966.609	-0.173	0.043	-4.058	4.94E-05	0.000888505	Kifc3	protein_coding
ENSMUSG00000058503.11	1510.184	0.175	0.043	4.057	4.96E-05	0.000889668	Fam133b	protein_coding
ENSMUSG00000070639.5	5562.006	0.125	0.031	4.057	4.97E-05	0.000889668	Lrrc8b	protein_coding
ENSMUSG00000060257.2	875.266	-0.258	0.064	-4.057	4.97E-05	0.000889668	Scrt2	protein_coding
ENSMUSG00000033707.8	1706.935	-0.182	0.045	-4.051	5.09E-05	0.000910658	Lrrc24	protein_coding
ENSMUSG00000025372.16	20267.635	-0.109	0.027	-4.045	5.23E-05	0.000933689	Baiap2	protein_coding
ENSMUSG00000062209.15	4757.202	0.137	0.034	4.045	5.24E-05	0.000935336	Erbb4	protein_coding
ENSMUSG00000051518.7	583.086	-0.226	0.056	-4.043	5.28E-05	0.000940818	Rps19bp1	protein_coding
ENSMUSG00000038539.15	553.353	-0.242	0.060	-4.042	5.29E-05	0.000942044	Atf5	protein_coding
ENSMUSG00000028833.13	74877.484	-0.171	0.042	-4.042	5.31E-05	0.0009429	Ncdn	protein_coding
ENSMUSG00000034254.17	5873.582	-0.134	0.033	-4.041	5.33E-05	0.000946517	Agpat1	protein_coding
ENSMUSG00000029208.16	2455.072	0.161	0.040	4.039	5.36E-05	0.000949981	Guf1	protein_coding
ENSMUSG00000021936.13	6893.509	0.121	0.030	4.037	5.41E-05	0.000957892	Mapk8	protein_coding
ENSMUSG00000036442.8	1224.673	-0.195	0.048	-4.032	5.53E-05	0.000978312	Thap11	protein_coding
ENSMUSG00000027660.16	4301.093	0.136	0.034	4.030	5.58E-05	0.000984922	Skil	protein_coding
ENSMUSG00000020238.14	3382.963	-0.159	0.040	-4.030	5.58E-05	0.000984922	Ncln	protein_coding
ENSMUSG00000060002.14	2289.315	0.147	0.036	4.029	5.61E-05	0.000988402	Chpt1	protein_coding
ENSMUSG00000030660.9	1700.586	0.166	0.041	4.022	5.77E-05	0.001014592	Pik3c2a	protein_coding
ENSMUSG00000022452.14	1843.344	-0.167	0.042	-4.021	5.79E-05	0.00101667	Smdt1	protein_coding
ENSMUSG00000030739.18	1801.879	-0.182	0.045	-4.019	5.83E-05	0.001023942	Myh14	protein_coding

ENSMUSG00000061136.14	2131.757	0.150	0.037	4.018	5.87E-05	0.001028287	Prpf40a	protein_coding
ENSMUSG00000055675.6	7191.365	-0.113	0.028	-4.014	5.98E-05	0.001046171	Kbtbd11	protein_coding
ENSMUSG00000034636.9	13777.982	0.096	0.024	4.011	6.04E-05	0.001056245	Zyg11b	protein_coding
ENSMUSG00000038552.14	3920.748	-0.128	0.032	-4.008	6.11E-05	0.001067463	Fndc4	protein_coding
ENSMUSG00000001016.12	3901.820	0.124	0.031	4.007	6.15E-05	0.001072221	Ilf2	protein_coding
ENSMUSG00000031007.2	12846.466	0.118	0.029	4.005	6.19E-05	0.001078896	Atp6ap2	protein_coding
ENSMUSG00000010608.15	8299.323	0.107	0.027	4.003	6.24E-05	0.001086461	Rbm25	protein_coding
ENSMUSG00000029328.15	7926.574	0.114	0.028	4.002	6.28E-05	0.001091336	Hnrnpdl	protein_coding
ENSMUSG00000059852.7	299.643	-0.299	0.075	-4.001	6.30E-05	0.001093023	Kcng2	protein_coding
ENSMUSG00000055022.14	19856.627	0.099	0.025	4.001	6.32E-05	0.001095371	Cntn1	protein_coding
ENSMUSG00000029071.16	6646.963	-0.130	0.032	-3.999	6.36E-05	0.001101371	Dvl1	protein_coding
ENSMUSG00000066392.11	14638.938	0.106	0.027	3.995	6.47E-05	0.001118634	Nrxn3	protein_coding
ENSMUSG00000025104.13	5721.694	0.126	0.032	3.991	6.58E-05	0.001137222	Hdgfrp3	protein_coding
ENSMUSG00000036550.16	7045.852	0.096	0.024	3.989	6.63E-05	0.001143655	Cnot1	protein_coding
ENSMUSG00000032740.16	4407.494	0.135	0.034	3.989	6.64E-05	0.001144045	Ccdc88a	protein_coding
ENSMUSG00000021587.5	3960.658	0.142	0.036	3.988	6.67E-05	0.001147926	Pcsk1	protein_coding
ENSMUSG00000017418.13	1451.123	0.186	0.047	3.985	6.74E-05	0.001157767	Arl5b	protein_coding
ENSMUSG00000013663.7	7340.641	0.126	0.032	3.984	6.77E-05	0.001161715	Pten	protein_coding
ENSMUSG00000049577.15	1265.901	-0.231	0.058	-3.978	6.96E-05	0.001193063	Zfpm1	protein_coding
ENSMUSG00000052144.8	3270.947	0.146	0.037	3.977	6.97E-05	0.001193063	Ppp4r2	protein_coding
ENSMUSG00000053550.13	15473.744	-0.137	0.035	-3.977	6.98E-05	0.001193063	Shisa7	protein_coding
ENSMUSG00000052137.11	750.519	0.219	0.055	3.974	7.06E-05	0.001206155	Rbm12b2	protein_coding
ENSMUSG00000035621.13	3133.392	-0.184	0.046	-3.972	7.12E-05	0.001215266	Midn	protein_coding
ENSMUSG00000001270.9	45300.289	-0.125	0.031	-3.971	7.15E-05	0.001217825	Ckb	protein_coding
ENSMUSG00000070576.4	2122.775	-0.173	0.044	-3.971	7.15E-05	0.001217825	Mn1	protein_coding
ENSMUSG00000020948.9	1562.364	0.167	0.042	3.969	7.22E-05	0.001227643	Klhl28	protein_coding
ENSMUSG00000037243.17	2306.567	-0.164	0.041	-3.968	7.25E-05	0.001230676	Zfp692	protein_coding
ENSMUSG00000042104.18	2126.291	0.154	0.039	3.967	7.28E-05	0.001234713	Uggt2	protein_coding
ENSMUSG00000028403.15	3207.858	0.146	0.037	3.967	7.29E-05	0.001234713	Zdhhc21	protein_coding
ENSMUSG00000030605.15	8758.575	-0.122	0.031	-3.965	7.35E-05	0.001243446	Mfge8	protein_coding
ENSMUSG00000047141.5	1396.310	0.162	0.041	3.964	7.36E-05	0.001243446	Zfp654	protein_coding
ENSMUSG00000043099.4	167.706	-0.308	0.078	-3.963	7.40E-05	0.001248498	Hic1	protein_coding
ENSMUSG00000025907.14	5217.351	0.146	0.037	3.962	7.43E-05	0.001252859	Rb1cc1	protein_coding

ENSMUSG00000004319.15	9346.959	0.097	0.025	3.962	7.44E-05	0.001253524	Clcn3	protein_coding
ENSMUSG00000110185.1	2185.219	0.180	0.046	3.959	7.54E-05	0.00126812	Igip	protein_coding
ENSMUSG00000074622.4	896.402	-0.224	0.057	-3.957	7.60E-05	0.001276473	Mafb	protein_coding
ENSMUSG00000018102.4	546.925	-0.236	0.060	-3.955	7.67E-05	0.001285083	Hist1h2bc	protein_coding
ENSMUSG00000041378.1	1295.476	-0.235	0.059	-3.955	7.67E-05	0.001285083	Cldn5	protein_coding
ENSMUSG00000004980.16	23414.877	0.085	0.021	3.951	7.77E-05	0.001298932	Hnrnpa2b1	protein_coding
ENSMUSG00000035437.15	4855.177	0.121	0.031	3.951	7.77E-05	0.001298932	Rabgap1	protein_coding
ENSMUSG00000022139.16	10395.162	0.123	0.031	3.950	7.83E-05	0.001306781	Mbnl2	protein_coding
ENSMUSG00000021537.8	1582.711	0.179	0.045	3.947	7.91E-05	0.001319931	Cetn3	protein_coding
ENSMUSG00000074968.11	5203.695	0.187	0.047	3.946	7.93E-05	0.001319931	Ano3	protein_coding
ENSMUSG00000021453.2	567.834	-0.242	0.061	-3.946	7.93E-05	0.001319931	Gadd45g	protein_coding
ENSMUSG00000021102.4	879.454	-0.207	0.052	-3.944	8.00E-05	0.001329569	Glrx5	protein_coding
ENSMUSG00000047213.14	3844.728	0.143	0.036	3.941	8.10E-05	0.001340933	Ythdf3	protein_coding
ENSMUSG00000058835.14	5010.307	0.124	0.032	3.941	8.10E-05	0.001340933	Abi1	protein_coding
ENSMUSG00000036890.13	1895.182	0.167	0.042	3.941	8.11E-05	0.001340933	Gtdc1	protein_coding
ENSMUSG00000042156.15	5300.384	-0.124	0.031	-3.940	8.14E-05	0.001344518	Dzip1	protein_coding
ENSMUSG00000019966.18	1957.055	0.186	0.047	3.939	8.20E-05	0.001352747	Kitl	protein_coding
ENSMUSG00000067851.11	6551.314	0.120	0.030	3.938	8.21E-05	0.001353206	Arfgef1	protein_coding
ENSMUSG00000051285.17	4803.962	0.132	0.033	3.937	8.26E-05	0.001360409	Pcmtd1	protein_coding
ENSMUSG00000066613.14	1989.437	0.167	0.042	3.935	8.32E-05	0.001368095	Zfp932	protein_coding
ENSMUSG00000021910.15	30861.392	-0.103	0.026	-3.931	8.47E-05	0.001389478	Nisch	protein_coding
ENSMUSG00000024797.12	1974.195	-0.165	0.042	-3.930	8.48E-05	0.001390183	Vps51	protein_coding
ENSMUSG00000037270.18	11067.487	0.102	0.026	3.926	8.65E-05	0.001415958	4932438A13Rik	protein_coding
ENSMUSG00000029403.14	2254.115	0.148	0.038	3.925	8.68E-05	0.001417354	Cdkl2	protein_coding
ENSMUSG00000017707.9	12835.124	0.104	0.027	3.925	8.69E-05	0.001417354	Serinc3	protein_coding
ENSMUSG00000009013.5	8345.902	-0.121	0.031	-3.925	8.69E-05	0.001417354	Dynll1	protein_coding
ENSMUSG00000020063.16	997.776	0.188	0.048	3.922	8.78E-05	0.001430229	Sirt1	protein_coding
ENSMUSG00000025037.6	1626.704	0.158	0.040	3.918	8.91E-05	0.00145044	Maoa	protein_coding
ENSMUSG00000022568.16	3684.525	-0.155	0.039	-3.918	8.95E-05	0.001453865	Scrib	protein_coding
ENSMUSG00000040268.17	5026.734	0.130	0.033	3.917	8.95E-05	0.001453865	Plekha1	protein_coding
ENSMUSG00000022799.4	2750.728	-0.143	0.037	-3.909	9.25E-05	0.001500457	Arhgap31	protein_coding
ENSMUSG00000037098.17	7912.251	-0.114	0.029	-3.909	9.27E-05	0.001502288	Rab11fip3	protein_coding
ENSMUSG00000034647.14	4780.631	0.121	0.031	3.907	9.34E-05	0.001510971	Ankrd12	protein_coding

ENSMUSG00000027965.15	1801.733	0.186	0.048	3.907	9.35E-05	0.001510971	Olfm3	protein_coding
ENSMUSG00000061650.6	1645.251	-0.169	0.043	-3.906	9.40E-05	0.001517923	Med9	protein_coding
ENSMUSG00000029838.11	9071.774	0.120	0.031	3.905	9.42E-05	0.001518331	Ptn	protein_coding
ENSMUSG00000029088.16	2677.045	0.154	0.039	3.902	9.54E-05	0.001535975	Kcnip4	protein_coding
ENSMUSG00000038271.17	3124.942	-0.143	0.037	-3.900	9.63E-05	0.001549383	Iffo1	protein_coding
ENSMUSG00000054452.10	13443.879	-0.178	0.046	-3.897	9.74E-05	0.001565556	Aes	protein_coding
ENSMUSG00000028879.4	6683.620	0.106	0.027	3.896	9.80E-05	0.001572844	Stx12	protein_coding
ENSMUSG00000015468.14	489.075	-0.227	0.058	-3.893	9.90E-05	0.001586131	Notch4	protein_coding
ENSMUSG00000031955.10	2641.582	-0.151	0.039	-3.891	9.98E-05	0.001596701	Bcar1	protein_coding
ENSMUSG00000037221.13	524.173	-0.253	0.065	-3.889	0.00010049	0.001605786	Mospd3	protein_coding
ENSMUSG00000021147.17	3716.970	0.129	0.033	3.888	0.000101159	0.001614649	Wdr37	protein_coding
ENSMUSG00000028252.20	1594.252	0.159	0.041	3.887	0.000101348	0.00161583	Ccnc	protein_coding
ENSMUSG00000038502.16	6790.840	-0.172	0.044	-3.887	0.000101578	0.001617674	Ptov1	protein_coding
ENSMUSG00000072949.6	326.881	-0.289	0.074	-3.885	0.000102364	0.001628349	Acot1	protein_coding
ENSMUSG00000035478.14	4084.279	-0.122	0.031	-3.884	0.000102832	0.001633947	Mbd3	protein_coding
ENSMUSG00000033014.12	3997.628	0.123	0.032	3.881	0.000104103	0.001652283	Trim33	protein_coding
ENSMUSG00000037826.5	4023.394	0.179	0.046	3.876	0.000105987	0.001679534	Ppm1k	protein_coding
ENSMUSG00000060988.12	1689.871	0.196	0.051	3.876	0.000106058	0.001679534	Galnt13	protein_coding
ENSMUSG00000064329.13	6720.124	0.133	0.034	3.876	0.000106273	0.001679584	Scn1a	protein_coding
ENSMUSG00000020571.12	6811.223	-0.129	0.033	-3.876	0.000106366	0.001679584	Pdia6	protein_coding
ENSMUSG00000026361.9	2575.398	0.146	0.038	3.875	0.000106419	0.001679584	Cdc73	protein_coding
ENSMUSG00000055707.13	2668.208	-0.133	0.034	-3.875	0.000106584	0.001680313	Klhl26	protein_coding
ENSMUSG00000002058.13	945.810	-0.192	0.049	-3.875	0.000106797	0.001681779	Unc119	protein_coding
ENSMUSG00000022483.16	131.226	-0.300	0.077	-3.874	0.00010715	0.001685463	Col2a1	protein_coding
ENSMUSG00000038679.16	1150.411	0.181	0.047	3.873	0.000107459	0.001688429	Trps1	protein_coding
ENSMUSG00000020594.14	10067.640	0.110	0.028	3.872	0.000107785	0.001691669	Pum2	protein_coding
ENSMUSG00000027162.7	3312.108	0.148	0.038	3.871	0.000108372	0.0016971	Lin7c	protein_coding
ENSMUSG00000030016.14	6253.297	0.127	0.033	3.868	0.000109528	0.001713304	Zfp638	protein_coding
ENSMUSG00000020827.18	19435.072	-0.095	0.024	-3.867	0.000110159	0.001721259	Mink1	protein_coding
ENSMUSG00000084845.9	1727.111	-0.182	0.047	-3.867	0.000110322	0.001721905	Tmem240	protein_coding
ENSMUSG00000023991.16	1764.699	-0.174	0.045	-3.866	0.00011073	0.001724608	Foxp4	protein_coding
ENSMUSG00000044134.9	727.135	-0.214	0.055	-3.866	0.00011074	0.001724608	Fam109a	protein_coding
ENSMUSG00000059518.14	1311.902	-0.162	0.042	-3.865	0.000111207	0.001728093	Znhit1	protein_coding

ENSMUSG00000023067.13	1378.022	-0.208	0.054	-3.865	0.000111209	0.001728093	Cdkn1a	protein_coding
ENSMUSG00000066551.12	3091.449	0.129	0.033	3.863	0.000112057	0.001739349	Hmgb1	protein_coding
ENSMUSG00000041459.15	11620.754	0.104	0.027	3.859	0.000113968	0.001767069	Tardbp	protein_coding
ENSMUSG00000079442.12	1858.365	-0.145	0.038	-3.857	0.000114793	0.001777903	St6galnac4	protein_coding
ENSMUSG00000025862.14	3380.772	0.154	0.040	3.853	0.000116727	0.00180588	Stag2	protein_coding
ENSMUSG00000062931.15	909.360	0.181	0.047	3.848	0.000119181	0.001841815	Zfp938	protein_coding
ENSMUSG00000034165.16	1161.221	-0.209	0.054	-3.847	0.000119623	0.001846618	Ccnd3	protein_coding
ENSMUSG00000061024.8	787.701	-0.201	0.052	-3.846	0.000120278	0.001854705	Rrs1	protein_coding
ENSMUSG00000041638.18	5761.890	-0.116	0.030	-3.843	0.000121428	0.001870387	Gcn1l1	protein_coding
ENSMUSG00000039137.18	2368.206	-0.219	0.057	-3.841	0.000122754	0.001888749	Whrn	protein_coding
ENSMUSG00000019796.12	8042.054	-0.104	0.027	-3.840	0.000123087	0.001891813	Lrp11	protein_coding
ENSMUSG00000051146.2	1924.225	-0.192	0.050	-3.840	0.000123239	0.001892089	Camk2n2	protein_coding
ENSMUSG00000035632.16	2933.993	-0.132	0.034	-3.838	0.000123822	0.001898956	Cnot3	protein_coding
ENSMUSG00000040229.11	419.140	0.253	0.066	3.836	0.000124951	0.001912898	Gpr34	protein_coding
ENSMUSG00000028919.11	1137.518	-0.204	0.053	-3.836	0.000125002	0.001912898	Arhgef19	protein_coding
ENSMUSG00000046836.13	3236.576	0.125	0.033	3.836	0.000125207	0.001913962	Brox	protein_coding
ENSMUSG00000005871.14	23902.687	0.117	0.031	3.831	0.000127486	0.001946679	Apc	protein_coding
ENSMUSG00000007850.16	14203.591	0.105	0.027	3.829	0.000128691	0.001962953	Hnrnph1	protein_coding
ENSMUSG00000020627.10	3732.272	-0.145	0.038	-3.825	0.000130783	0.001992705	Klhl29	protein_coding
ENSMUSG00000047879.16	6869.032	0.139	0.036	3.821	0.000132897	0.002022735	Usp14	protein_coding
ENSMUSG00000070520.4	908.389	-0.189	0.050	-3.820	0.000133648	0.002031972	Nsmce3	protein_coding
ENSMUSG00000080316.10	2956.360	0.126	0.033	3.819	0.000133831	0.002032557	Spaca6	protein_coding
ENSMUSG00000039661.14	4133.984	-0.149	0.039	-3.817	0.000134835	0.002045603	Dusp26	protein_coding
ENSMUSG00000020130.9	1633.491	0.151	0.039	3.816	0.000135618	0.002053205	Tbc1d15	protein_coding
ENSMUSG00000030495.12	1675.080	-0.179	0.047	-3.816	0.000135627	0.002053205	Slc7a10	protein_coding
ENSMUSG00000025626.16	1581.392	0.151	0.040	3.812	0.000137886	0.002085161	Phf6	protein_coding
ENSMUSG00000035596.14	10480.264	-0.131	0.034	-3.811	0.000138614	0.002093923	Mboat7	protein_coding
ENSMUSG00000017740.17	35519.639	-0.128	0.034	-3.810	0.000139153	0.002099809	Slc12a5	protein_coding
ENSMUSG00000030729.17	23544.131	0.112	0.029	3.809	0.000139501	0.00210261	Pgm2l1	protein_coding
ENSMUSG00000040124.5	439.603	0.232	0.061	3.809	0.000139637	0.00210261	Gorab	protein_coding
ENSMUSG00000025020.11	7466.519	-0.150	0.039	-3.808	0.000140089	0.00210717	Slit1	protein_coding
ENSMUSG00000074922.1	419.314	-0.264	0.069	-3.807	0.000140464	0.002110549	Fam122a	protein_coding
ENSMUSG00000034227.7	473.187	-0.287	0.075	-3.805	0.000141739	0.00212745	Foxj1	protein_coding

ENSMUSG00000034610.14	2173.374	0.145	0.038	3.801	0.000143915	0.002155897	Zcchc11	protein_coding
ENSMUSG00000021054.6	1725.853	0.165	0.043	3.801	0.000143941	0.002155897	Sgpp1	protein_coding
ENSMUSG00000046785.8	10590.732	0.106	0.028	3.800	0.000144417	0.002160742	Epm2aip1	protein_coding
ENSMUSG00000069049.11	2658.010	0.196	0.052	3.799	0.0001451	0.00216865	Eif2s3y	protein_coding
ENSMUSG00000023175.15	13864.858	-0.190	0.050	-3.797	0.000146454	0.002186568	Bsg	protein_coding
ENSMUSG00000027339.15	3532.234	-0.168	0.044	-3.794	0.000148241	0.002210907	Rassf2	protein_coding
ENSMUSG00000037808.13	1045.376	0.200	0.053	3.788	0.000152089	0.002265902	Fam76b	protein_coding
ENSMUSG00000052726.15	1728.732	0.161	0.043	3.786	0.000152928	0.002275993	Kcnt2	protein_coding
ENSMUSG00000025894.11	1329.652	0.157	0.041	3.783	0.00015488	0.002302619	Aasdhppt	protein_coding
ENSMUSG00000040797.16	12574.165	-0.136	0.036	-3.781	0.000156071	0.00231787	Iqsec3	protein_coding
ENSMUSG00000024661.6	47673.458	-0.259	0.069	-3.781	0.000156483	0.002321543	Fth1	protein_coding
ENSMUSG00000070803.6	159.831	-0.294	0.078	-3.780	0.000156995	0.002326691	Cited4	protein_coding
ENSMUSG00000058355.8	4905.180	0.110	0.029	3.779	0.0001572	0.002327294	Abce1	protein_coding
ENSMUSG00000051579.10	1296.991	0.170	0.045	3.776	0.000159489	0.002358699	Tceal8	protein_coding
ENSMUSG00000028719.8	3093.061	0.126	0.033	3.773	0.00016139	0.002384233	Cmpk1	protein_coding
ENSMUSG00000032637.15	11366.564	-0.120	0.032	-3.772	0.000161712	0.002384233	Atxn2l	protein_coding
ENSMUSG00000050222.10	298.849	-0.272	0.072	-3.772	0.000161813	0.002384233	Il17d	protein_coding
ENSMUSG00000033307.7	3077.700	-0.159	0.042	-3.772	0.000162062	0.002384233	Mif	protein_coding
ENSMUSG00000059474.13	1622.313	0.162	0.043	3.770	0.000163051	0.002396291	Mbtd1	protein_coding
ENSMUSG00000036529.17	13115.271	-0.095	0.025	-3.769	0.000164016	0.00240796	Sbf1	protein_coding
ENSMUSG00000011831.16	4898.911	0.135	0.036	3.768	0.000164787	0.002416769	Evi5	protein_coding
ENSMUSG00000006699.17	12147.616	0.116	0.031	3.766	0.000166057	0.002432849	Cdc42	protein_coding
ENSMUSG00000040010.10	3577.071	-0.130	0.034	-3.762	0.000168233	0.002462174	Slc7a5	protein_coding
ENSMUSG00000063704.12	199.479	-0.291	0.077	-3.762	0.000168586	0.002464777	Mapk15	protein_coding
ENSMUSG00000027466.15	5162.882	-0.126	0.033	-3.760	0.000170022	0.002482566	Rbck1	protein_coding
ENSMUSG00000033964.12	1408.394	0.160	0.042	3.759	0.000170299	0.002482566	Zbtb41	protein_coding
ENSMUSG00000054477.15	2111.492	-0.161	0.043	-3.759	0.000170425	0.002482566	Kcnn2	protein_coding
ENSMUSG00000064367.1	151372.231	0.084	0.022	3.759	0.000170507	0.002482566	mt-Nd5	protein_coding
ENSMUSG00000030276.19	1201.185	-0.203	0.054	-3.759	0.000170793	0.002484172	Ttll3	protein_coding
ENSMUSG00000005625.15	3947.932	-0.115	0.031	-3.757	0.000172059	0.002499994	Psmd4	protein_coding
ENSMUSG00000012296.15	1517.257	-0.155	0.041	-3.756	0.000172798	0.002508144	Tjap1	protein_coding
ENSMUSG00000030237.14	2531.906	0.158	0.042	3.754	0.000173927	0.002521945	Slco1a4	protein_coding
ENSMUSG00000034994.10	54876.426	-0.100	0.027	-3.754	0.000174207	0.002523412	Eef2	protein_coding

ENSMUSG00000026753.5	2592.882	0.145	0.039	3.753	0.000174886	0.002530633	Ppp6c	protein_coding
ENSMUSG00000062604.11	12371.138	0.091	0.024	3.752	0.000175635	0.002538877	Srpk2	protein_coding
ENSMUSG00000028937.14	12117.157	-0.112	0.030	-3.749	0.000177496	0.002563139	Acot7	protein_coding
ENSMUSG00000047013.15	8527.247	-0.132	0.035	-3.748	0.000178128	0.00256964	Fbxo41	protein_coding
ENSMUSG00000025738.7	50611.909	-0.124	0.033	-3.746	0.00017983	0.002591535	Fbxl16	protein_coding
ENSMUSG00000021959.15	1773.887	-0.176	0.047	-3.742	0.00018226	0.002621544	Lats2	protein_coding
ENSMUSG00000037622.14	4442.374	-0.143	0.038	-3.742	0.000182375	0.002621544	Wdtc1	protein_coding
ENSMUSG00000063887.13	3216.069	0.132	0.035	3.742	0.00018247	0.002621544	Nlgn1	protein_coding
ENSMUSG00000045967.11	7787.592	0.096	0.026	3.741	0.000183111	0.002628072	Gpr158	protein_coding
ENSMUSG00000004460.16	2562.283	-0.165	0.044	-3.741	0.000183643	0.002633023	Dnajb11	protein_coding
ENSMUSG00000000804.14	9146.575	0.090	0.024	3.739	0.00018509	0.002649443	Usp32	protein_coding
ENSMUSG00000019726.11	5389.856	0.115	0.031	3.738	0.000185164	0.002649443	Lyst	protein_coding
ENSMUSG00000034259.7	725.291	-0.202	0.054	-3.736	0.000186866	0.002671083	Exosc4	protein_coding
ENSMUSG00000041935.9	3876.310	0.130	0.035	3.735	0.000187907	0.002683245	AW549877	protein_coding
ENSMUSG00000022037.14	29068.723	-0.103	0.028	-3.732	0.000190023	0.002708213	Clu	protein_coding
ENSMUSG00000028063.15	2615.100	-0.164	0.044	-3.732	0.000190223	0.002708213	Lmna	protein_coding
ENSMUSG00000038664.16	21283.798	0.095	0.025	3.732	0.000190232	0.002708213	Herc1	protein_coding
ENSMUSG00000109336.1	5085.260	-0.125	0.033	-3.731	0.000190811	0.002711178	Samd4b	protein_coding
ENSMUSG00000022203.6	728.461	-0.239	0.064	-3.731	0.000190825	0.002711178	Efs	protein_coding
ENSMUSG00000034312.14	16351.984	-0.106	0.029	-3.730	0.000191242	0.002711628	Iqsec1	protein_coding
ENSMUSG00000031202.5	2265.154	0.149	0.040	3.729	0.000192131	0.002721508	Rab39b	protein_coding
ENSMUSG00000045589.7	17877.210	0.138	0.037	3.729	0.000192569	0.002724453	Frrs1l	protein_coding
ENSMUSG00000074212.7	6138.466	0.103	0.028	3.728	0.000192726	0.002724453	Dnajb14	protein_coding
ENSMUSG00000035597.19	3227.089	0.134	0.036	3.727	0.00019384	0.002734712	Prpf39	protein_coding
ENSMUSG00000025575.14	3365.043	-0.126	0.034	-3.725	0.000195403	0.002753781	Cant1	protein_coding
ENSMUSG00000031827.13	3256.800	-0.177	0.048	-3.725	0.000195582	0.002753781	Cotl1	protein_coding
ENSMUSG00000063358.15	29527.718	0.100	0.027	3.721	0.000198664	0.002794387	Mapk1	protein_coding
ENSMUSG00000033793.12	7455.181	0.114	0.031	3.716	0.00020244	0.002844653	Atp6v1h	protein_coding
ENSMUSG00000036896.5	1476.221	-0.163	0.044	-3.715	0.000203081	0.002850819	C1qc	protein_coding
ENSMUSG00000057715.13	4004.061	0.146	0.039	3.713	0.000204922	0.002873808	A830018L16Rik	protein_coding
ENSMUSG00000068328.9	2853.439	-0.151	0.041	-3.710	0.000206946	0.002899304	Aup1	protein_coding
ENSMUSG00000025451.15	2447.654	0.127	0.034	3.710	0.000207303	0.002901039	Paip1	protein_coding
ENSMUSG00000021733.9	1627.277	0.149	0.040	3.710	0.000207482	0.002901039	Slc4a7	protein_coding

ENSMUSG00000079523.8	2574.010	-0.217	0.058	-3.709	0.000207909	0.002904135	Tmsb10	protein_coding
ENSMUSG00000051790.15	13196.225	-0.130	0.035	-3.708	0.000208546	0.002910151	Nlgn2	protein_coding
ENSMUSG00000005893.14	7353.490	0.096	0.026	3.708	0.000208819	0.002910586	Nr2c2	protein_coding
ENSMUSG00000002718.14	3337.236	0.122	0.033	3.708	0.000209155	0.002910586	Cse1l	protein_coding
ENSMUSG00000009470.16	4049.508	0.121	0.033	3.708	0.000209197	0.002910586	Tnpo1	protein_coding
ENSMUSG00000025793.15	4599.888	-0.120	0.032	-3.704	0.000212033	0.002947137	Hgs	protein_coding
ENSMUSG00000048578.11	8476.310	-0.131	0.035	-3.704	0.000212248	0.002947209	Mlec	protein_coding
ENSMUSG00000039936.18	2298.493	-0.145	0.039	-3.703	0.000212768	0.002951531	Pik3cd	protein_coding
ENSMUSG00000035226.5	6408.633	-0.128	0.035	-3.700	0.000215733	0.002989721	Rims4	protein_coding
ENSMUSG00000026103.14	23210.226	0.091	0.025	3.699	0.000216606	0.00299886	Gls	protein_coding
ENSMUSG00000031885.14	1076.848	0.173	0.047	3.698	0.000217192	0.003004031	Cbfb	protein_coding
ENSMUSG00000028525.16	6049.386	0.111	0.030	3.698	0.000217548	0.003006001	Pde4b	protein_coding
ENSMUSG00000050812.18	7687.097	0.093	0.025	3.697	0.000218238	0.003009628	AI314180	protein_coding
ENSMUSG00000027797.15	46345.698	0.083	0.022	3.695	0.00022003	0.003031383	Dclk1	protein_coding
ENSMUSG00000002799.6	3010.339	-0.152	0.041	-3.694	0.00022077	0.003036273	Jag2	protein_coding
ENSMUSG00000030583.16	3978.172	-0.120	0.032	-3.694	0.000220816	0.003036273	Sipa1l3	protein_coding
ENSMUSG00000032827.15	17732.730	0.108	0.029	3.690	0.000224321	0.003081468	Ppp1r9a	protein_coding
ENSMUSG00000070808.4	1522.987	-0.180	0.049	-3.685	0.000229088	0.003143883	Gltscr1	protein_coding
ENSMUSG00000045665.4	1427.447	-0.155	0.042	-3.677	0.000235861	0.003232862	Mfsd5	protein_coding
ENSMUSG00000022797.15	4932.903	0.169	0.046	3.677	0.000236031	0.003232862	Tfrc	protein_coding
ENSMUSG00000030431.8	40.243	-0.262	0.071	-3.673	0.000239456	NA	Tmem238	protein_coding
ENSMUSG00000027273.13	101399.955	0.074	0.020	3.671	0.000241331	0.003300402	Snap25	protein_coding
ENSMUSG00000020458.16	28796.564	0.098	0.027	3.671	0.00024143	0.003300402	Rtn4	protein_coding
ENSMUSG00000031557.16	1518.222	0.158	0.043	3.669	0.000243038	0.003319168	Plekha2	protein_coding
ENSMUSG00000001761.7	616.586	-0.208	0.057	-3.667	0.000245563	0.003350401	Smo	protein_coding
ENSMUSG00000067199.4	361.897	-0.243	0.066	-3.664	0.000248458	0.003385093	Frat1	protein_coding
ENSMUSG00000023391.8	328.657	-0.274	0.075	-3.664	0.000248586	0.003385093	Dlx2	protein_coding
ENSMUSG00000028132.15	3460.879	0.149	0.041	3.663	0.000249115	0.003386342	Tmem56	protein_coding
ENSMUSG00000007610.15	1257.687	-0.188	0.051	-3.663	0.000249158	0.003386342	Gtpbp3	protein_coding
ENSMUSG00000022551.7	5871.997	-0.120	0.033	-3.663	0.000249689	0.003390292	Cyc1	protein_coding
ENSMUSG00000026986.14	901.230	0.193	0.053	3.662	0.000250369	0.003396246	Hnmt	protein_coding
ENSMUSG00000052581.13	1444.430	0.193	0.053	3.661	0.00025103	0.003401935	Lrrtm4	protein_coding
ENSMUSG00000024234.6	1127.433	0.178	0.049	3.660	0.000251778	0.003408794	Mtpap	protein_coding

ENSMUSG00000005610.17	31177.861	0.083	0.023	3.660	0.000252341	0.003410107	Eif4g2	protein_coding
ENSMUSG00000046432.12	4685.756	-0.110	0.030	-3.660	0.000252359	0.003410107	Bex3	protein_coding
ENSMUSG00000027006.13	3971.255	0.119	0.032	3.659	0.000252711	0.003411603	Dnajc10	protein_coding
ENSMUSG00000022811.16	5052.471	0.098	0.027	3.656	0.000255786	0.003449812	Zfp148	protein_coding
ENSMUSG00000021750.15	20236.274	-0.144	0.039	-3.655	0.000256796	0.003460114	Fam107a	protein_coding
ENSMUSG00000058756.13	38093.905	-0.135	0.037	-3.655	0.000257641	0.003468188	Thra	protein_coding
ENSMUSG00000063273.11	3315.081	0.112	0.031	3.654	0.000258196	0.003472345	Naa15	protein_coding
ENSMUSG00000024325.8	1734.000	-0.165	0.045	-3.654	0.000258665	0.003472915	Ring1	protein_coding
ENSMUSG00000024425.3	15580.524	-0.126	0.034	-3.653	0.000258731	0.003472915	Ndfip1	protein_coding
ENSMUSG00000035773.6	432.181	-0.241	0.066	-3.653	0.000259441	0.003476721	Kiss1r	protein_coding
ENSMUSG00000033350.7	6240.698	-0.131	0.036	-3.653	0.000259508	0.003476721	Chst2	protein_coding
ENSMUSG00000039585.15	8822.190	0.092	0.025	3.652	0.000260578	0.003487734	Myo9a	protein_coding
ENSMUSG00000034488.14	5436.135	0.157	0.043	3.650	0.000261928	0.003501568	Edil3	protein_coding
ENSMUSG00000020015.10	9014.222	0.120	0.033	3.650	0.000262108	0.003501568	Cdk17	protein_coding
ENSMUSG00000041685.15	2509.995	0.144	0.039	3.648	0.000264138	0.003525015	Fcho2	protein_coding
ENSMUSG00000051451.6	2638.249	-0.164	0.045	-3.648	0.000264364	0.003525015	Crebzf	protein_coding
ENSMUSG00000028552.13	12314.819	0.109	0.030	3.645	0.000267664	0.003565651	Eps15	protein_coding
ENSMUSG00000004056.15	4656.402	-0.121	0.033	-3.643	0.000269644	0.003588635	Akt2	protein_coding
ENSMUSG00000063646.16	4331.434	-0.139	0.038	-3.639	0.000273857	0.003641265	Jakmip1	protein_coding
ENSMUSG00000038738.15	32130.870	-0.181	0.050	-3.637	0.000275408	0.00365844	Shank1	protein_coding
ENSMUSG00000044628.4	3894.366	-0.146	0.040	-3.634	0.000278834	0.003698916	Rnf208	protein_coding
ENSMUSG00000040447.15	3755.046	-0.113	0.031	-3.634	0.00027898	0.003698916	Spns2	protein_coding
ENSMUSG00000030788.16	3309.213	0.134	0.037	3.633	0.000280648	0.003716309	Rnf141	protein_coding
ENSMUSG00000089862.8	1466.257	0.154	0.042	3.632	0.000280819	0.003716309	Umad1	protein_coding
ENSMUSG00000051674.14	5330.973	0.117	0.032	3.629	0.00028438	0.003759898	Dcun1d4	protein_coding
ENSMUSG00000000093.6	101.014	-0.282	0.078	-3.625	0.00028932	0.003821631	Tbx2	protein_coding
ENSMUSG00000050373.13	1787.290	-0.161	0.044	-3.624	0.0002906	0.003831569	Snx21	protein_coding
ENSMUSG00000046096.7	3451.773	0.131	0.036	3.623	0.000290866	0.003831569	BC030336	protein_coding
ENSMUSG00000021189.11	1630.956	0.153	0.042	3.623	0.000290888	0.003831569	Atxn3	protein_coding
ENSMUSG00000021326.12	1074.768	-0.196	0.054	-3.623	0.000291679	0.003838392	Trim27	protein_coding
ENSMUSG00000038301.15	6332.728	0.134	0.037	3.622	0.000292006	0.003839113	Snx10	protein_coding
ENSMUSG00000027206.13	3966.601	0.141	0.039	3.621	0.000293067	0.003849478	Cops2	protein_coding
ENSMUSG00000060882.5	3915.755	0.115	0.032	3.621	0.00029386	0.003856302	Kcnd2	protein_coding

ENSMUSG00000057335.11	5456.041	0.104	0.029	3.614	0.000301868	0.0039577	Cep170	protein_coding
ENSMUSG00000025240.9	4441.391	0.120	0.033	3.613	0.000302432	0.003961411	Sacm1l	protein_coding
ENSMUSG00000045608.6	532.576	-0.208	0.058	-3.613	0.000302919	0.00396411	Dbx2	protein_coding
ENSMUSG00000025609.15	5707.663	0.100	0.028	3.612	0.000303599	0.003969318	Mkln1	protein_coding
ENSMUSG00000021400.7	2840.929	-0.162	0.045	-3.608	0.000308153	0.004021399	Wrnip1	protein_coding
ENSMUSG00000029028.14	2985.254	-0.115	0.032	-3.608	0.000308812	0.004026263	Lrrc47	protein_coding
ENSMUSG00000014776.7	882.424	-0.204	0.057	-3.606	0.000310608	0.004045946	Nol3	protein_coding
ENSMUSG00000024870.5	5671.922	-0.113	0.031	-3.601	0.000317196	0.00412104	Rab1b	protein_coding
ENSMUSG00000062300.14	304.768	-0.255	0.071	-3.601	0.000317231	0.00412104	Nectin2	protein_coding
ENSMUSG00000058153.15	16697.456	-0.104	0.029	-3.601	0.00031725	0.00412104	Sez6l	protein_coding
ENSMUSG00000044098.14	1839.150	0.148	0.041	3.600	0.000318823	0.004137652	Rsbn1	protein_coding
ENSMUSG00000028780.13	1510.248	0.195	0.054	3.598	0.000320834	0.00415992	Sema3c	protein_coding
ENSMUSG00000020396.8	2499.672	-0.178	0.049	-3.596	0.000323053	0.004181344	Nefh	protein_coding
ENSMUSG00000038319.14	1901.623	-0.200	0.055	-3.596	0.00032308	0.004181344	Kcnh2	protein_coding
ENSMUSG00000067336.6	8649.416	0.126	0.035	3.596	0.000323542	0.004183493	Bmpr2	protein_coding
ENSMUSG00000058975.7	10061.760	-0.134	0.037	-3.592	0.000328016	0.004237152	Kcnc1	protein_coding
ENSMUSG00000074733.14	4031.357	0.139	0.039	3.592	0.000328294	0.004237152	Zfp950	protein_coding
ENSMUSG00000034006.16	856.322	-0.176	0.049	-3.591	0.000329594	0.004243894	Pqlc1	protein_coding
ENSMUSG00000029436.9	11660.321	-0.124	0.035	-3.591	0.000329679	0.004243894	Mmp17	protein_coding
ENSMUSG00000035863.14	12457.747	-0.122	0.034	-3.591	0.000329719	0.004243894	Palm	protein_coding
ENSMUSG00000022314.9	5778.816	0.124	0.035	3.588	0.000332637	0.004277541	Rad21	protein_coding
ENSMUSG00000063253.11	4454.781	0.116	0.032	3.588	0.000333621	0.004286287	Scoc	protein_coding
ENSMUSG00000036095.11	7669.177	0.141	0.039	3.586	0.000336149	0.004314825	Dgkb	protein_coding
ENSMUSG00000029203.16	4698.903	0.111	0.031	3.585	0.000336463	0.004314937	Ube2k	protein_coding
ENSMUSG00000028528.16	19738.325	0.094	0.026	3.585	0.000336827	0.004315678	Dnajc6	protein_coding
ENSMUSG00000031278.12	4641.231	0.118	0.033	3.585	0.000337503	0.004320405	Acsl4	protein_coding
ENSMUSG00000019254.16	6588.564	-0.126	0.035	-3.583	0.000340157	0.004350432	Ppp1r12c	protein_coding
ENSMUSG00000025393.12	47150.991	-0.074	0.021	-3.582	0.000340688	0.004353265	Atp5b	protein_coding
ENSMUSG00000026241.5	142.068	-0.277	0.077	-3.582	0.000341395	0.004358355	Nppc	protein_coding
ENSMUSG00000044562.12	339.366	-0.260	0.073	-3.577	0.000347001	0.004425907	Rasip1	protein_coding
ENSMUSG00000058793.14	36320.828	-0.074	0.021	-3.576	0.000349139	0.004449158	Cds2	protein_coding
ENSMUSG00000027634.14	23822.698	0.068	0.019	3.575	0.000350648	0.004464351	Ndrg3	protein_coding
ENSMUSG00000022151.16	1726.599	0.146	0.041	3.574	0.00035154	0.004471664	Ttc33	protein_coding

ENSMUSG00000027582.16	3736.896	-0.127	0.036	-3.573	0.000353062	0.004486525	Zgpat	protein_coding
ENSMUSG00000024164.15	77.285	-0.240	0.067	-3.573	0.000353345	0.004486525	C3	protein_coding
ENSMUSG00000041483.14	2332.896	0.123	0.034	3.570	0.000357408	0.004530714	Zfp281	protein_coding
ENSMUSG00000059895.12	2602.634	-0.143	0.040	-3.570	0.000357468	0.004530714	Ptp4a3	protein_coding
ENSMUSG00000023990.18	383.046	-0.239	0.067	-3.569	0.000358127	0.004531529	Tfeb	protein_coding
ENSMUSG00000044501.17	524.901	0.221	0.062	3.569	0.000358207	0.004531529	Zfp758	protein_coding
ENSMUSG00000035969.15	8931.934	-0.109	0.031	-3.567	0.000361347	0.004560332	Rusc2	protein_coding
ENSMUSG00000031748.16	41778.564	-0.072	0.020	-3.567	0.000361423	0.004560332	Gnao1	protein_coding
ENSMUSG00000027438.14	29828.806	0.096	0.027	3.566	0.000361939	0.004562768	Napb	protein_coding
ENSMUSG00000028804.20	7522.495	-0.128	0.036	-3.565	0.000363633	0.004575926	Csmd2	protein_coding
ENSMUSG00000022553.15	2649.414	-0.122	0.034	-3.564	0.000364974	0.004588711	Maf1	protein_coding
ENSMUSG00000030824.17	3914.507	-0.114	0.032	-3.564	0.000365921	0.004592676	Nucb1	protein_coding
ENSMUSG00000007594.10	4333.406	-0.170	0.048	-3.564	0.000365941	0.004592676	Hapln4	protein_coding
ENSMUSG00000019943.10	34531.921	0.096	0.027	3.562	0.000367555	0.004608826	Atp2b1	protein_coding
ENSMUSG00000030180.15	2883.416	0.121	0.034	3.561	0.000369247	0.004625922	Kdm5a	protein_coding
ENSMUSG00000031214.13	3052.052	0.111	0.031	3.560	0.000370293	0.004634912	Ophn1	protein_coding
ENSMUSG00000032745.17	4681.432	0.112	0.031	3.556	0.000376967	0.004714265	Gpbp1	protein_coding
ENSMUSG00000057342.15	1642.843	-0.178	0.050	-3.554	0.000379001	0.004735502	Sphk2	protein_coding
ENSMUSG00000021209.12	1926.483	0.150	0.042	3.552	0.000381708	0.004765092	Ppp4r4	protein_coding
ENSMUSG00000061904.12	16465.016	-0.095	0.027	-3.552	0.000382882	0.004775515	Slc25a3	protein_coding
ENSMUSG00000020745.15	28358.370	0.101	0.028	3.550	0.000385488	0.004803771	Pafah1b1	protein_coding
ENSMUSG00000035798.14	7142.290	0.098	0.028	3.548	0.000388145	0.004832611	Zdhhc17	protein_coding
ENSMUSG00000031592.10	5050.383	0.130	0.037	3.547	0.000389063	0.004839766	Pcm1	protein_coding
ENSMUSG00000048379.8	910.172	0.173	0.049	3.546	0.000391185	0.004861865	Socs4	protein_coding
ENSMUSG00000029212.11	9798.783	0.083	0.024	3.541	0.000397895	0.004940395	Gabrb1	protein_coding
ENSMUSG00000021113.5	2954.815	0.127	0.036	3.541	0.000398204	0.004940395	Snapc1	protein_coding
ENSMUSG00000043460.6	7992.118	-0.144	0.041	-3.540	0.000399449	0.004951478	Elfn2	protein_coding
ENSMUSG00000063802.5	3194.451	-0.147	0.042	-3.540	0.000400096	0.004955138	Hspbp1	protein_coding
ENSMUSG00000073664.11	1934.775	0.140	0.039	3.540	0.00040049	0.004955663	Nbeal1	protein_coding
ENSMUSG00000004054.8	1271.192	-0.156	0.044	-3.536	0.000406173	0.005014959	Map3k11	protein_coding
ENSMUSG00000040488.17	4583.160	-0.156	0.044	-3.536	0.000406349	0.005014959	Ltbp4	protein_coding
ENSMUSG00000034839.5	1478.734	-0.142	0.040	-3.532	0.000412808	0.005090217	Larp6	protein_coding
ENSMUSG00000033423.16	5161.290	-0.094	0.027	-3.531	0.000413301	0.005091843	Eri3	protein_coding

ENSMUSG00000041058.15	2095.836	0.140	0.040	3.531	0.000414531	0.005102527	Wwp1	protein_coding
ENSMUSG00000075232.5	3271.674	0.124	0.035	3.528	0.00041833	0.005144796	Amd1	protein_coding
ENSMUSG00000026864.13	19795.951	-0.133	0.038	-3.527	0.00042058	0.005167966	Hspa5	protein_coding
ENSMUSG00000054000.5	94.976	-0.273	0.078	-3.525	0.000423192	0.005195526	Tusc1	protein_coding
ENSMUSG00000074211.4	297.930	-0.236	0.067	-3.523	0.000427122	0.005237018	Sdhaf1	protein_coding
ENSMUSG00000050288.6	411.682	-0.255	0.072	-3.523	0.000427315	0.005237018	Fzd2	protein_coding
ENSMUSG00000037710.8	2405.474	-0.128	0.036	-3.522	0.000428769	0.005248463	Cisd1	protein_coding
ENSMUSG00000019878.8	1464.577	0.151	0.043	3.521	0.000429366	0.005248463	Hsf2	protein_coding
ENSMUSG00000016995.17	702.461	-0.192	0.054	-3.520	0.000430842	0.00526016	Matn4	protein_coding
ENSMUSG00000068748.7	12731.922	0.118	0.033	3.520	0.000431069	0.00526016	Ptprz1	protein_coding
ENSMUSG00000030086.16	1933.121	-0.136	0.039	-3.518	0.000434824	0.005301389	Chchd6	protein_coding
ENSMUSG00000007050.17	216.569	-0.257	0.073	-3.517	0.000435919	0.00531015	Lsm2	protein_coding
ENSMUSG00000028971.4	255.446	-0.260	0.074	-3.516	0.000437781	0.005327042	Cort	protein_coding
ENSMUSG00000026820.5	1659.747	-0.149	0.042	-3.516	0.000438062	0.005327042	Ptges2	protein_coding
ENSMUSG00000017485.10	5475.424	0.124	0.035	3.515	0.00043918	0.005334737	Top2b	protein_coding
ENSMUSG00000026791.14	1225.822	-0.166	0.047	-3.515	0.000439796	0.005334737	Slc2a8	protein_coding
ENSMUSG00000024392.17	11694.491	-0.106	0.030	-3.515	0.00043983	0.005334737	Bag6	protein_coding
ENSMUSG00000004264.17	2681.058	-0.127	0.036	-3.514	0.000442164	0.005354826	Phb2	protein_coding
ENSMUSG00000020444.19	2960.142	-0.127	0.036	-3.513	0.000442246	0.005354826	Guk1	protein_coding
ENSMUSG00000070802.5	22025.654	-0.118	0.033	-3.513	0.000442679	0.005355469	Pnmal2	protein_coding
ENSMUSG00000024949.17	8737.071	-0.085	0.024	-3.509	0.000450024	0.005439652	Sf1	protein_coding
ENSMUSG00000037326.10	4290.107	-0.131	0.037	-3.508	0.000450669	0.005442781	Capn15	protein_coding
ENSMUSG00000033900.13	6789.646	0.113	0.032	3.507	0.000453308	0.005469965	Map9	protein_coding
ENSMUSG00000046593.3	97.146	0.268	0.076	3.507	0.000453735	0.005470433	Tmem215	protein_coding
ENSMUSG00000022837.13	1432.661	0.179	0.051	3.506	0.000454607	0.00547626	Iqcb1	protein_coding
ENSMUSG00000037999.13	5931.370	0.127	0.036	3.503	0.000459898	0.005535273	Arap2	protein_coding
ENSMUSG00000031860.17	479.662	-0.210	0.060	-3.502	0.000462129	0.005557383	Pbx4	protein_coding
ENSMUSG00000024294.13	9211.754	0.106	0.030	3.501	0.0004642	0.005573328	Mib1	protein_coding
ENSMUSG00000040446.3	3672.494	0.107	0.030	3.501	0.000464246	0.005573328	Rprd1a	protein_coding
ENSMUSG00000025940.6	1464.413	-0.150	0.043	-3.500	0.000465929	0.005588766	Tmem70	protein_coding
ENSMUSG00000021259.5	10067.359	-0.137	0.039	-3.498	0.000468441	0.00561412	Cyp46a1	protein_coding
ENSMUSG00000032187.16	9331.978	-0.099	0.028	-3.496	0.000472183	0.00565253	Smarca4	protein_coding
ENSMUSG00000036499.9	3153.080	0.143	0.041	3.496	0.000472448	0.00565253	Eea1	protein_coding

ENSMUSG00000008348.9	1368.538	-0.158	0.045	-3.495	0.000473279	0.005653172	Ubc	protein_coding
ENSMUSG00000058240.13	3214.449	0.115	0.033	3.495	0.000473304	0.005653172	Cryzl1	protein_coding
ENSMUSG00000026781.15	3085.593	0.135	0.039	3.495	0.000473706	0.005653185	Acbd5	protein_coding
ENSMUSG00000024335.19	8629.357	-0.084	0.024	-3.495	0.000474233	0.005654691	Brd2	protein_coding
ENSMUSG00000026434.12	5108.303	0.153	0.044	3.493	0.00047703	0.005678428	Nucks1	protein_coding
ENSMUSG00000042216.13	10116.678	-0.133	0.038	-3.491	0.000482099	0.005730376	Sgsm1	protein_coding
ENSMUSG00000021096.11	11674.416	0.087	0.025	3.490	0.000482207	0.005730376	Ppm1a	protein_coding
ENSMUSG00000000223.13	5815.648	0.110	0.032	3.490	0.000482811	0.005732726	Drp2	protein_coding
ENSMUSG00000021773.10	432.534	-0.210	0.060	-3.488	0.000485758	0.005762861	Comtd1	protein_coding
ENSMUSG00000026889.12	2731.056	0.123	0.035	3.488	0.000487533	0.005779053	Rbm18	protein_coding
ENSMUSG00000022091.5	1953.552	-0.163	0.047	-3.484	0.000493064	0.005839699	Sorbs3	protein_coding
ENSMUSG00000042807.15	2774.596	0.130	0.037	3.481	0.000499202	0.005907435	Hecw2	protein_coding
ENSMUSG00000018974.7	2701.335	-0.116	0.033	-3.481	0.000500257	0.005914952	Sart3	protein_coding
ENSMUSG00000022228.13	7106.188	0.088	0.025	3.480	0.000502213	0.005933106	Zscan26	protein_coding
ENSMUSG00000048707.9	814.301	-0.217	0.062	-3.479	0.000502953	0.005936865	Tprn	protein_coding
ENSMUSG00000004473.10	191.582	-0.263	0.076	-3.479	0.000504198	0.005946578	Clec11a	protein_coding
ENSMUSG00000048834.16	5211.346	0.145	0.042	3.477	0.000507906	0.005985307	Vstm2a	protein_coding
ENSMUSG00000027433.5	2363.510	0.128	0.037	3.476	0.00050854	0.005987772	Xrn2	protein_coding
ENSMUSG00000034566.10	5223.329	0.108	0.031	3.476	0.000509333	0.0059921	Atp5h	protein_coding
ENSMUSG00000024579.7	1094.501	-0.171	0.049	-3.476	0.000509785	0.005992424	Pcyox1l	protein_coding
ENSMUSG00000005057.13	266.087	-0.248	0.071	-3.471	0.000517946	0.006083275	Sh2b2	protein_coding
ENSMUSG00000073639.5	4814.906	0.117	0.034	3.471	0.000519286	0.006093935	Rab18	protein_coding
ENSMUSG00000001911.16	10874.911	-0.170	0.049	-3.470	0.000520772	0.006106292	Nfix	protein_coding
ENSMUSG00000034891.13	12073.629	-0.146	0.042	-3.468	0.000523451	0.006132605	Sncb	protein_coding
ENSMUSG00000007656.13	12599.868	0.124	0.036	3.468	0.000524293	0.00613736	Arpp19	protein_coding
ENSMUSG00000031703.8	11346.985	0.085	0.025	3.467	0.000527212	0.006161351	Itfg1	protein_coding
ENSMUSG00000038628.8	1999.807	0.132	0.038	3.467	0.000527217	0.006161351	Polr3k	protein_coding
ENSMUSG00000021945.8	5020.241	0.099	0.028	3.466	0.000527953	0.006164847	Zmym2	protein_coding
ENSMUSG00000019841.15	2901.951	0.139	0.040	3.463	0.000534624	0.006237578	Rev3l	protein_coding
ENSMUSG00000028124.15	2394.901	0.142	0.041	3.462	0.000535935	0.006242606	Gclm	protein_coding
ENSMUSG00000039126.9	4225.706	0.130	0.037	3.462	0.000535941	0.006242606	Prune2	protein_coding
ENSMUSG00000042155.3	3062.745	0.145	0.042	3.461	0.000538447	0.006266612	Klhl23	protein_coding
ENSMUSG00000049600.8	1115.392	-0.191	0.055	-3.460	0.000539749	0.00627659	Zbtb45	protein_coding

ENSMUSG00000041763.14	2918.798	0.122	0.035	3.460	0.000540632	0.00628167	Tpp2	protein_coding
ENSMUSG00000046798.14	2342.337	0.117	0.034	3.458	0.000544373	0.006318227	Cldn12	protein_coding
ENSMUSG00000039697.16	4676.619	0.105	0.031	3.458	0.000544675	0.006318227	Ncoa7	protein_coding
ENSMUSG00000042699.11	7470.811	0.108	0.031	3.457	0.000545494	0.006318299	Dhx9	protein_coding
ENSMUSG00000024383.8	2222.111	0.135	0.039	3.457	0.000545578	0.006318299	Map3k2	protein_coding
ENSMUSG00000038127.14	3459.048	0.123	0.036	3.457	0.000546442	0.006323116	Ccdc50	protein_coding
ENSMUSG00000022619.5	18252.596	-0.103	0.030	-3.455	0.000549525	0.006353575	Mapk8ip2	protein_coding
ENSMUSG00000003378.9	12661.282	-0.147	0.043	-3.452	0.000555731	0.006413728	Grik5	protein_coding
ENSMUSG00000033184.14	4298.847	0.118	0.034	3.452	0.000555848	0.006413728	Tmed7	protein_coding
ENSMUSG00000062961.7	803.326	-0.201	0.058	-3.452	0.000556093	0.006413728	Ccdc177	protein_coding
ENSMUSG00000010154.7	881.424	-0.175	0.051	-3.451	0.000557525	0.006424984	Spire2	protein_coding
ENSMUSG00000031696.9	8271.302	0.109	0.031	3.449	0.000562603	0.006478204	Vps35	protein_coding
ENSMUSG00000039809.10	17433.164	-0.121	0.035	-3.447	0.000565829	0.006510033	Gabbr2	protein_coding
ENSMUSG00000073775.4	448.991	-0.223	0.065	-3.446	0.000569408	0.006545871	Kti12	protein_coding
ENSMUSG00000022971.18	466.869	-0.220	0.064	-3.445	0.000570219	0.006549854	Ifnar2	protein_coding
ENSMUSG00000002413.15	7727.602	0.107	0.031	3.445	0.000571053	0.006554084	Braf	protein_coding
ENSMUSG00000028278.14	6029.259	0.095	0.028	3.445	0.000571742	0.006556655	Rragd	protein_coding
ENSMUSG00000041180.13	1558.120	0.146	0.042	3.444	0.000572642	0.006561645	Hectd2	protein_coding
ENSMUSG00000020794.13	2123.614	0.126	0.037	3.444	0.000573884	0.00657053	Ube2g1	protein_coding
ENSMUSG00000075376.10	3462.216	0.147	0.043	3.442	0.000577777	0.006607137	Rc3h2	protein_coding
ENSMUSG00000025409.14	3334.404	-0.157	0.046	-3.442	0.000578215	0.006607137	Mbd6	protein_coding
ENSMUSG00000079184.10	2723.726	0.122	0.035	3.442	0.000578488	0.006607137	Mphosph8	protein_coding
ENSMUSG00000035278.9	809.064	-0.186	0.054	-3.440	0.0005817	0.006638451	Plekhj1	protein_coding
ENSMUSG00000025586.17	5259.590	0.106	0.031	3.439	0.000584287	0.006662577	Cpeb1	protein_coding
ENSMUSG00000051166.10	7507.555	0.114	0.033	3.436	0.000590515	0.006728144	Eml5	protein_coding
ENSMUSG00000026831.16	166.521	-0.264	0.077	-3.436	0.000591138	0.006729804	1700007K13Rik	protein_coding
ENSMUSG00000024811.11	8264.088	0.093	0.027	3.434	0.000594847	0.00676656	Tnks2	protein_coding
ENSMUSG00000079598.3	1700.123	-0.137	0.040	-3.430	0.000604521	0.006869955	Clec2l	protein_coding
ENSMUSG00000087408.10	2163.305	-0.145	0.042	-3.429	0.000604911	0.006869955	Cers1	protein_coding
ENSMUSG00000024287.7	1646.626	0.162	0.047	3.428	0.00060865	0.006906849	Thoc1	protein_coding
ENSMUSG00000027342.14	1253.220	-0.156	0.045	-3.427	0.000609931	0.00691582	Pcna	protein_coding
ENSMUSG00000028127.10	3518.106	0.124	0.036	3.426	0.000611973	0.006933399	Abcd3	protein_coding
ENSMUSG00000025870.10	658.683	-0.205	0.060	-3.424	0.000616447	0.006978476	Arl10	protein_coding

ENSMUSG00000029815.12	405.744	-0.236	0.069	-3.424	0.000617888	0.006989177	Malsu1	protein_coding
ENSMUSG00000024096.16	4807.583	-0.100	0.029	-3.423	0.000618642	0.006992088	Ralbp1	protein_coding
ENSMUSG00000071711.11	886.680	-0.165	0.048	-3.423	0.00061973	0.006998773	Mpst	protein_coding
ENSMUSG00000016344.14	1230.770	-0.175	0.051	-3.422	0.000622082	0.007019712	Ppdpf	protein_coding
ENSMUSG00000058624.12	18026.028	0.120	0.035	3.421	0.000623741	0.007032802	Gda	protein_coding
ENSMUSG00000038145.17	4788.358	-0.113	0.033	-3.420	0.000627349	0.007067828	Snrk	protein_coding
ENSMUSG00000062115.15	6353.833	-0.143	0.042	-3.419	0.000629491	0.007086304	Rai1	protein_coding
ENSMUSG00000024347.16	3388.725	-0.138	0.040	-3.416	0.000635592	0.007149267	Psd2	protein_coding
ENSMUSG00000028776.14	367.695	-0.255	0.075	-3.414	0.000639852	0.00719116	Tinagl1	protein_coding
ENSMUSG00000043059.16	1024.499	-0.165	0.048	-3.414	0.000640337	0.00719116	Zfp513	protein_coding
ENSMUSG00000072941.5	383.878	-0.258	0.076	-3.413	0.00064165	0.007200176	Sod3	protein_coding
ENSMUSG00000025508.13	2725.956	-0.139	0.041	-3.412	0.000645058	0.007232173	Rplp2	protein_coding
ENSMUSG00000020366.18	11766.295	0.091	0.027	3.412	0.000645566	0.007232173	Mapk9	protein_coding
ENSMUSG00000057963.9	3037.746	-0.118	0.035	-3.412	0.000646041	0.007232173	Itpk1	protein_coding
ENSMUSG00000031765.8	3412.140	-0.166	0.049	-3.410	0.000649665	0.007258634	Mt1	protein_coding
ENSMUSG00000046556.7	1690.039	-0.166	0.049	-3.410	0.000650533	0.007258634	Zfp319	protein_coding
ENSMUSG00000039483.10	1846.790	-0.150	0.044	-3.410	0.000650715	0.007258634	Asb6	protein_coding
ENSMUSG00000033054.7	1514.406	0.164	0.048	3.409	0.000650854	0.007258634	Npat	protein_coding
ENSMUSG00000046994.9	1499.290	0.145	0.043	3.408	0.000653986	0.007286383	Mars2	protein_coding
ENSMUSG00000029629.17	3178.344	0.125	0.037	3.408	0.000654733	0.007288945	Phf14	protein_coding
ENSMUSG00000038888.8	404.871	-0.220	0.065	-3.408	0.000655288	0.007289362	Ctu1	protein_coding
ENSMUSG00000026796.16	2345.033	-0.144	0.042	-3.407	0.000656009	0.007291629	Fam129b	protein_coding
ENSMUSG00000040721.9	5939.219	-0.125	0.037	-3.407	0.00065706	0.007297565	Zfhx2	protein_coding
ENSMUSG00000040537.17	13537.484	0.076	0.022	3.406	0.000659065	0.007314064	Adam22	protein_coding
ENSMUSG00000074738.2	888.257	-0.181	0.053	-3.406	0.000660047	0.007319198	Fndc10	protein_coding
ENSMUSG00000028883.17	945.381	0.170	0.050	3.405	0.000662172	0.007336996	Sema3a	protein_coding
ENSMUSG00000049482.16	854.706	-0.167	0.049	-3.402	0.000668217	0.007398152	Ctu2	protein_coding
ENSMUSG00000026516.8	5259.000	0.099	0.029	3.401	0.000671249	0.0074161	Nvl	protein_coding
ENSMUSG00000014959.12	4505.750	-0.102	0.030	-3.401	0.000671355	0.0074161	Gorasp2	protein_coding
ENSMUSG00000004677.17	6520.185	-0.107	0.031	-3.401	0.000671416	0.0074161	Myo9b	protein_coding
ENSMUSG00000034265.7	1955.903	-0.174	0.051	-3.400	0.000674577	0.007443278	Zdhhc14	protein_coding
ENSMUSG00000002661.14	327.537	-0.226	0.067	-3.399	0.000675329	0.007443278	Alkbh7	protein_coding
ENSMUSG00000022051.15	5039.275	0.113	0.033	3.399	0.000675939	0.007443278	Bnip3l	protein_coding

ENSMUSG00000012017.3	129.578	-0.263	0.077	-3.399	0.00067599	0.007443278	Scarf2	protein_coding
ENSMUSG00000029291.11	12512.966	0.088	0.026	3.392	0.000692936	0.007617967	Rufy3	protein_coding
ENSMUSG00000028986.12	5032.600	0.104	0.031	3.391	0.00069525	0.007637455	Klhl7	protein_coding
ENSMUSG00000045817.8	817.580	-0.224	0.066	-3.390	0.000698369	0.007665745	Zfp36l2	protein_coding
ENSMUSG00000042032.13	4853.874	0.111	0.033	3.390	0.000699168	0.007668535	Mat2b	protein_coding
ENSMUSG00000028221.3	2005.598	0.127	0.038	3.388	0.00070418	0.007717507	Tmem55a	protein_coding
ENSMUSG00000053293.9	4969.677	-0.113	0.034	-3.384	0.00071566	0.007834232	Pom121	protein_coding
ENSMUSG00000038393.14	1533.545	-0.212	0.063	-3.383	0.000715942	0.007834232	Txnip	protein_coding
ENSMUSG00000026234.12	12042.221	0.100	0.030	3.381	0.000721785	0.007892034	Ncl	protein_coding
ENSMUSG00000011589.8	2335.155	-0.121	0.036	-3.381	0.00072248	0.007893516	Fsd1	protein_coding
ENSMUSG00000036111.8	359.009	-0.227	0.067	-3.380	0.000725051	0.007915466	Lmo1	protein_coding
ENSMUSG00000027346.15	4381.756	0.116	0.034	3.378	0.000730013	0.007957637	Gpcpd1	protein_coding
ENSMUSG00000040009.6	3968.154	-0.105	0.031	-3.378	0.000730043	0.007957637	Gnaz	protein_coding
ENSMUSG00000001229.7	4690.090	-0.104	0.031	-3.377	0.0007339	0.007993503	Dpp9	protein_coding
ENSMUSG00000034220.7	5400.391	-0.141	0.042	-3.376	0.000734838	0.007997531	Gpc1	protein_coding
ENSMUSG00000038467.15	6234.715	-0.099	0.029	-3.375	0.000736821	0.008012925	Chmp4b	protein_coding
ENSMUSG00000058966.13	2889.995	-0.143	0.042	-3.373	0.000744141	0.00808629	Fam57b	protein_coding
ENSMUSG00000047658.6	2097.567	-0.155	0.046	-3.370	0.000750658	0.008150822	Gal3st3	protein_coding
ENSMUSG00000027282.17	3741.159	0.102	0.030	3.370	0.000752059	0.008159754	Mtch2	protein_coding
ENSMUSG00000030275.6	28808.730	0.079	0.023	3.367	0.000760288	0.008242693	Etnk1	protein_coding
ENSMUSG00000045515.3	4033.301	-0.160	0.048	-3.366	0.000763676	0.008273063	Pou3f3	protein_coding
ENSMUSG00000059273.9	3620.776	-0.119	0.035	-3.365	0.00076503	0.008281363	Zc3h4	protein_coding
ENSMUSG00000043881.8	2301.050	0.135	0.040	3.364	0.000769375	0.008322008	Kbtbd7	protein_coding
ENSMUSG00000033128.8	3938.673	-0.138	0.041	-3.363	0.000770802	0.008331048	Gga1	protein_coding
ENSMUSG00000060510.14	2120.750	0.122	0.036	3.363	0.000772028	0.008337901	Zfp266	protein_coding
ENSMUSG00000040734.14	278.776	-0.236	0.070	-3.361	0.000775955	0.008373902	Ppp1r13l	protein_coding
ENSMUSG00000024360.7	4078.360	0.105	0.031	3.359	0.000782515	0.00843823	Etf1	protein_coding
ENSMUSG00000027763.13	4287.676	0.118	0.035	3.358	0.000786309	0.008472659	Mbnl1	protein_coding
ENSMUSG00000037062.13	4405.240	0.096	0.028	3.356	0.000789487	0.008500403	Sh3glb1	protein_coding
ENSMUSG00000045039.9	14917.441	-0.132	0.039	-3.354	0.000795496	0.008558564	Megf8	protein_coding
ENSMUSG00000046792.8	514.953	-0.207	0.062	-3.352	0.000801075	0.008612018	Zfp787	protein_coding
ENSMUSG00000006019.15	653.241	-0.185	0.055	-3.352	0.000802751	0.008623459	Dhx34	protein_coding
ENSMUSG00000027993.16	15899.492	0.089	0.026	3.351	0.000805729	0.008648856	Trim2	protein_coding

ENSMUSG00000042272.17	4019.280	0.101	0.030	3.346	0.000819547	0.008790481	Sestd1	protein_coding
ENSMUSG00000036492.12	219.643	-0.259	0.077	-3.346	0.000820759	0.008796794	Rnf39	protein_coding
ENSMUSG00000021025.8	794.081	-0.236	0.071	-3.345	0.000821468	0.0087977	Nfkbia	protein_coding
ENSMUSG00000022337.6	2355.328	0.130	0.039	3.344	0.000825264	0.008818547	Emc2	protein_coding
ENSMUSG00000027401.9	158.423	0.261	0.078	3.344	0.000825292	0.008818547	Tgm3	protein_coding
ENSMUSG00000015671.11	3369.978	0.104	0.031	3.344	0.00082679	0.008824467	Psma2	protein_coding
ENSMUSG00000047824.13	2998.569	-0.116	0.035	-3.344	0.000827098	0.008824467	Pygo2	protein_coding
ENSMUSG00000057182.14	3437.671	0.111	0.033	3.343	0.000829186	0.008840056	Scn3a	protein_coding
ENSMUSG00000046351.10	1998.731	0.143	0.043	3.338	0.000843855	0.008989637	Zfp322a	protein_coding
ENSMUSG00000063406.11	1350.574	0.147	0.044	3.338	0.000844777	0.008992661	Tmed5	protein_coding
ENSMUSG00000000915.15	4917.733	-0.100	0.030	-3.337	0.000847578	0.009015668	Hip1r	protein_coding
ENSMUSG00000025732.5	274.915	-0.243	0.073	-3.335	0.000853073	0.009067275	Fam195a	protein_coding
ENSMUSG00000018849.6	6010.810	-0.123	0.037	-3.334	0.000856764	0.009099646	Wwc1	protein_coding
ENSMUSG00000024570.6	1011.667	-0.157	0.047	-3.334	0.000857422	0.009099778	Rbfa	protein_coding
ENSMUSG00000039470.15	2248.516	0.144	0.043	3.329	0.000870753	0.009234308	Zdhhc2	protein_coding
ENSMUSG00000038437.11	12141.616	-0.109	0.033	-3.329	0.000873018	0.009251362	Mllt6	protein_coding
ENSMUSG00000037296.7	646.029	-0.193	0.058	-3.325	0.000883533	0.009355753	Lsm1	protein_coding
ENSMUSG00000068882.13	4739.950	0.111	0.033	3.325	0.00088479	0.009362033	Ssb	protein_coding
ENSMUSG00000039656.16	3247.027	-0.127	0.038	-3.324	0.000885938	0.009367149	Rxrb	protein_coding
ENSMUSG00000028575.11	72.690	0.256	0.077	3.323	0.000889627	NA	Eqtn	protein_coding
ENSMUSG00000035131.14	879.241	0.192	0.058	3.321	0.000896052	0.009466984	Brinp3	protein_coding
ENSMUSG00000031865.16	23289.070	-0.099	0.030	-3.319	0.000902132	0.009524085	Dctn1	protein_coding
ENSMUSG00000033411.16	1163.940	0.163	0.049	3.319	0.000903301	0.009529281	Ctdspl2	protein_coding
ENSMUSG00000064293.14	2883.290	0.144	0.043	3.315	0.000915658	0.009652417	Cntn4	protein_coding
ENSMUSG00000035762.10	969.610	0.167	0.050	3.310	0.000931592	0.00980874	Tmem161b	protein_coding
ENSMUSG00000033569.17	5208.784	0.106	0.032	3.310	0.000931879	0.00980874	Adgrb3	protein_coding
ENSMUSG00000043929.16	784.702	0.196	0.059	3.308	0.000938475	0.009870137	Klhl15	protein_coding
ENSMUSG00000090015.8	1686.958	0.137	0.041	3.308	0.000939113	0.009870137	Gm15446	protein_coding
ENSMUSG00000028433.14	2677.349	-0.121	0.036	-3.308	0.00094048	0.009877145	Ubap2	protein_coding
ENSMUSG00000028282.12	856.403	0.169	0.051	3.307	0.000944394	0.009910863	Casp8ap2	protein_coding
ENSMUSG00000031786.7	148.729	-0.236	0.071	-3.305	0.000949804	0.009960221	Drc7	protein_coding
ENSMUSG00000036644.15	12650.004	-0.084	0.025	-3.304	0.000952321	0.009978723	Tbc1d9b	protein_coding
ENSMUSG00000052681.8	2943.987	0.110	0.033	3.304	0.000952984	0.009978723	Rap1b	protein_coding

ENSMUSG00000059325.14	1671.228	-0.180	0.054	-3.303	0.00095657	0.010008828	Hopx	protein_coding
ENSMUSG00000035559.9	444.680	-0.204	0.062	-3.302	0.000959781	0.010034977	Mpv17l2	protein_coding
ENSMUSG00000073411.11	1572.079	-0.220	0.067	-3.302	0.000960493	0.010034978	H2-D1	protein_coding
ENSMUSG00000039989.7	1945.186	-0.131	0.040	-3.301	0.000962519	0.010048696	Cbx4	protein_coding
ENSMUSG00000024958.12	3619.926	-0.118	0.036	-3.300	0.000968146	0.010099966	Gpr137	protein_coding
ENSMUSG00000036620.14	2486.763	-0.120	0.036	-3.299	0.00096983	0.010110045	Mgat4b	protein_coding
ENSMUSG00000026239.14	1137.089	-0.162	0.049	-3.299	0.00097162	0.010121221	Pde6d	protein_coding
ENSMUSG00000010080.15	52.725	-0.194	0.059	-3.297	0.000976777	NA	Epn3	protein_coding
ENSMUSG00000030967.15	2489.935	0.114	0.035	3.296	0.000979561	0.0101964	Zranb1	protein_coding
ENSMUSG00000028005.13	9567.633	0.120	0.036	3.295	0.00098298	0.010220499	Gucy1b3	protein_coding
ENSMUSG00000040123.16	2456.995	0.127	0.038	3.295	0.000983326	0.010220499	Zmym5	protein_coding
ENSMUSG00000028249.15	7637.736	0.093	0.028	3.294	0.000988168	0.010263258	Sdcbp	protein_coding
ENSMUSG00000023938.7	1890.005	-0.166	0.050	-3.292	0.000993009	0.010305239	Aars2	protein_coding
ENSMUSG00000029246.14	826.366	0.165	0.050	3.292	0.000993672	0.010305239	Ppat	protein_coding
ENSMUSG00000020608.7	2057.113	0.123	0.037	3.291	0.00099684	0.010323634	Smc6	protein_coding
ENSMUSG00000044576.6	709.716	-0.175	0.053	-3.291	0.000996911	0.010323634	Garem2	protein_coding
ENSMUSG00000021770.10	4247.179	0.098	0.030	3.291	0.000998698	0.010330755	Samd8	protein_coding
ENSMUSG00000023232.17	711.636	-0.187	0.057	-3.291	0.000999065	0.010330755	Serinc2	protein_coding
ENSMUSG00000060780.2	2235.204	-0.127	0.039	-3.289	0.001005085	0.010385384	Lrrtm1	protein_coding
ENSMUSG00000090000.1	906.811	0.167	0.051	3.289	0.001006772	0.010395199	Ier3ip1	protein_coding
ENSMUSG00000022292.15	1995.888	0.127	0.039	3.286	0.001016444	0.010478072	Rrm2b	protein_coding
ENSMUSG00000038822.15	3430.263	0.123	0.037	3.286	0.00101702	0.010478072	Hace1	protein_coding
ENSMUSG00000052751.16	1837.640	-0.159	0.048	-3.286	0.001017029	0.010478072	Repin1	protein_coding
ENSMUSG00000055421.8	5189.959	0.135	0.041	3.285	0.00101858	0.01048367	Pcdh9	protein_coding
ENSMUSG00000004892.13	12932.699	-0.130	0.039	-3.285	0.00101906	0.01048367	Bcan	protein_coding
ENSMUSG00000030062.7	6516.429	-0.091	0.028	-3.285	0.001019978	0.010485467	Rpn1	protein_coding
ENSMUSG00000074781.5	6408.179	0.091	0.028	3.285	0.001020917	0.010487471	Ube2n	protein_coding
ENSMUSG00000039219.18	3177.541	0.139	0.042	3.284	0.001024189	0.010513413	Arid4b	protein_coding
ENSMUSG00000040423.10	2495.050	0.150	0.046	3.284	0.001024954	0.010513609	Rc3h1	protein_coding
ENSMUSG00000041028.15	16381.306	0.081	0.025	3.282	0.001029288	0.010550394	Ghitm	protein_coding
ENSMUSG00000032932.14	2452.164	0.130	0.040	3.282	0.001031	0.010560255	Hspa13	protein_coding
ENSMUSG00000079509.10	1216.756	0.149	0.045	3.281	0.001032595	0.010568918	Zfx	protein_coding
ENSMUSG00000026374.14	4138.734	0.114	0.035	3.281	0.001036196	0.010598078	Tsn	protein_coding

ENSMUSG00000042834.14	1520.390	0.141	0.043	3.280	0.001037175	0.010600398	Nrep	protein_coding
ENSMUSG00000030030.5	1374.621	-0.135	0.041	-3.280	0.001039751	0.010611329	1700003E16Rik	protein_coding
ENSMUSG00000015461.15	3114.680	-0.116	0.036	-3.279	0.001043408	0.010634412	Atf6b	protein_coding
ENSMUSG00000042625.16	6523.260	-0.117	0.036	-3.279	0.001043522	0.010634412	Safb2	protein_coding
ENSMUSG00000048078.16	11210.618	-0.097	0.030	-3.276	0.001051896	0.010707286	Tenm4	protein_coding
ENSMUSG00000067608.4	82.546	0.121	0.037	3.276	0.001052192	0.010707286	Pcna-ps2	protein_coding
ENSMUSG00000060681.15	6340.197	0.098	0.030	3.275	0.001054849	0.01071885	Slc9a6	protein_coding
ENSMUSG00000025040.13	1562.073	0.152	0.047	3.273	0.001064185	0.010805922	Fundc1	protein_coding
ENSMUSG00000039220.16	2901.628	-0.135	0.041	-3.272	0.001066937	0.010826066	Ppp1r10	protein_coding
ENSMUSG00000022450.5	1721.600	-0.150	0.046	-3.272	0.001067949	0.010828541	Ndufa6	protein_coding
ENSMUSG00000039157.12	5637.059	-0.117	0.036	-3.271	0.001069794	0.010839454	Fam102a	protein_coding
ENSMUSG00000030020.13	12163.407	0.079	0.024	3.270	0.001074363	0.010877923	Prickle2	protein_coding
ENSMUSG00000023806.9	274.647	-0.233	0.071	-3.270	0.001076353	0.010890251	Rsph3b	protein_coding
ENSMUSG00000024479.2	7699.821	0.120	0.037	3.270	0.001077186	0.010890863	Mal2	protein_coding
ENSMUSG00000028691.12	4838.204	0.118	0.036	3.268	0.001081194	0.01092355	Prdx1	protein_coding
ENSMUSG00000078862.10	853.625	0.162	0.049	3.268	0.001082399	0.010927883	Gm14326	protein_coding
ENSMUSG00000074406.5	562.418	-0.192	0.059	-3.266	0.001091782	0.01101473	Zfp628	protein_coding
ENSMUSG00000025199.16	3538.759	0.110	0.034	3.264	0.001097039	0.011059844	Chuk	protein_coding
ENSMUSG00000060166.5	5776.769	-0.152	0.047	-3.263	0.001102649	0.011108455	Zdhhc8	protein_coding
ENSMUSG00000040710.10	854.910	0.166	0.051	3.263	0.001104082	0.011114948	St8sia4	protein_coding
ENSMUSG00000023025.15	2263.371	0.135	0.041	3.260	0.001112166	0.011188344	Larp4	protein_coding
ENSMUSG00000040549.16	9504.899	0.090	0.028	3.259	0.001119567	0.011254762	Ckap5	protein_coding
ENSMUSG00000042460.5	1130.914	0.171	0.053	3.258	0.001120667	0.011257794	C1galt1	protein_coding
ENSMUSG00000037351.9	15502.008	-0.074	0.023	-3.257	0.001123991	0.011283145	Actr1b	protein_coding
ENSMUSG00000034912.17	2397.017	0.123	0.038	3.257	0.001125093	0.011286164	Mdga2	protein_coding
ENSMUSG00000031661.12	735.868	-0.183	0.056	-3.256	0.001129441	0.011320972	Nkd1	protein_coding
ENSMUSG00000026037.14	1496.651	0.148	0.046	3.256	0.001130708	0.011320972	Orc2	protein_coding
ENSMUSG00000028975.16	2085.046	-0.130	0.040	-3.256	0.001130973	0.011320972	Pex14	protein_coding
ENSMUSG00000021967.3	654.203	-0.199	0.061	-3.255	0.00113421	0.011345316	Mrpl57	protein_coding
ENSMUSG00000041120.6	2304.267	-0.174	0.054	-3.255	0.001135604	0.011348028	Nbl1	protein_coding
ENSMUSG00000001729.14	7839.082	-0.095	0.029	-3.254	0.001136091	0.011348028	Akt1	protein_coding
ENSMUSG00000073433.10	1949.666	-0.148	0.046	-3.253	0.001143275	0.011410745	Arhgdig	protein_coding
ENSMUSG00000031431.13	3665.975	-0.154	0.047	-3.252	0.001144699	0.011410745	Tsc22d3	protein_coding

ENSMUSG00000027011.14	2595.216	0.120	0.037	3.252	0.001144799	0.011410745	Ube2e3	protein_coding
ENSMUSG00000020307.15	1248.299	-0.159	0.049	-3.251	0.001149793	0.011449701	Cdc34	protein_coding
ENSMUSG00000032580.12	9284.368	0.088	0.027	3.249	0.001156264	0.011500625	Rbm5	protein_coding
ENSMUSG00000023017.9	4202.407	-0.103	0.032	-3.249	0.001158385	0.011513595	Asic1	protein_coding
ENSMUSG00000062647.16	6556.315	-0.095	0.029	-3.249	0.001160023	0.011521747	Rpl7a	protein_coding
ENSMUSG00000022342.5	3981.111	0.127	0.039	3.248	0.00116211	0.01153435	Kcnv1	protein_coding
ENSMUSG00000025227.14	1287.587	-0.137	0.042	-3.248	0.001162954	0.011534607	Mfsd13a	protein_coding
ENSMUSG00000036466.17	832.379	-0.184	0.057	-3.246	0.001172227	0.011618398	Megf11	protein_coding
ENSMUSG00000031622.16	5257.382	-0.113	0.035	-3.244	0.00117991	0.011679163	Sin3b	protein_coding
ENSMUSG00000019802.13	4650.027	0.101	0.031	3.244	0.001180015	0.011679163	Sec63	protein_coding
ENSMUSG00000048807.2	1114.240	-0.183	0.056	-3.243	0.001180971	0.011680421	Slc35e4	protein_coding
ENSMUSG00000021294.7	891.365	-0.203	0.063	-3.242	0.001187944	0.011741155	Kif26a	protein_coding
ENSMUSG00000046364.14	3816.168	-0.139	0.043	-3.240	0.001194232	0.01179503	Rpl27a	protein_coding
ENSMUSG00000040455.17	2611.528	0.114	0.035	3.239	0.001197441	0.011818441	Usp45	protein_coding
ENSMUSG00000028522.16	1614.640	0.151	0.047	3.239	0.001198504	0.011820659	Mier1	protein_coding
ENSMUSG00000028312.19	625.633	0.178	0.055	3.237	0.001209496	0.011920727	Smc2	protein_coding
ENSMUSG00000040044.11	1500.497	0.149	0.046	3.236	0.001210654	0.011923803	Orc3	protein_coding
ENSMUSG00000058690.14	6122.043	0.086	0.027	3.233	0.001226965	0.012076012	Ccser2	protein_coding
ENSMUSG00000057561.9	2079.058	0.130	0.040	3.230	0.001237426	0.012170466	Eif1a	protein_coding
ENSMUSG00000044349.15	146191.718	0.070	0.022	3.229	0.001242187	0.012208778	Snhg11	protein_coding
ENSMUSG00000030061.16	3302.816	0.110	0.034	3.229	0.00124313	0.012209526	Uba3	protein_coding
ENSMUSG00000024073.14	11043.469	0.078	0.024	3.228	0.001245903	0.012228245	Birc6	protein_coding
ENSMUSG00000031134.16	3420.313	0.100	0.031	3.228	0.001247013	0.012230623	Rbmx	protein_coding
ENSMUSG00000000276.11	4939.458	0.107	0.033	3.226	0.001254276	0.012293297	Dgke	protein_coding
ENSMUSG00000005732.14	1458.257	-0.155	0.048	-3.226	0.001256647	0.012307975	Ranbp1	protein_coding
ENSMUSG00000032046.15	11123.209	-0.114	0.035	-3.225	0.001257803	0.012310745	Abhd12	protein_coding
ENSMUSG00000044700.15	5147.725	-0.116	0.036	-3.225	0.001259262	0.012316471	Tmem201	protein_coding
ENSMUSG00000070000.13	5323.079	-0.133	0.041	-3.224	0.001262348	0.012336226	Fcho1	protein_coding
ENSMUSG00000024109.18	17820.089	0.067	0.021	3.224	0.001263032	0.012336226	Nrxn1	protein_coding
ENSMUSG00000021596.16	3988.672	0.154	0.048	3.222	0.001271759	0.012412862	Mctp1	protein_coding
ENSMUSG00000022311.15	2725.836	0.124	0.038	3.222	0.001273945	0.012425594	Csmd3	protein_coding
ENSMUSG00000037486.18	2066.521	0.116	0.036	3.221	0.001279157	0.012467798	Asxl2	protein_coding
ENSMUSG00000021109.13	2897.629	0.128	0.040	3.220	0.001282863	0.012494086	Hif1a	protein_coding

ENSMUSG00000054499.9	1480.281	-0.174	0.054	-3.220	0.001283627	0.012494086	Dedd2	protein_coding
ENSMUSG00000021550.5	333.007	-0.217	0.068	-3.217	0.001295406	0.012600036	2210016F16Rik	protein_coding
ENSMUSG00000059851.15	1090.301	-0.146	0.045	-3.212	0.001316059	0.01278192	Kmt5c	protein_coding
ENSMUSG00000028603.15	2744.473	0.115	0.036	3.212	0.001316755	0.01278192	Scp2	protein_coding
ENSMUSG00000109901.1	1107.645	0.158	0.049	3.212	0.001316826	0.01278192	Chmp1b	protein_coding
ENSMUSG00000060073.9	3337.244	0.108	0.034	3.211	0.001323825	0.012841014	Psma3	protein_coding
ENSMUSG00000049796.4	130.381	-0.250	0.078	-3.207	0.001339931	0.012988296	Crh	protein_coding
ENSMUSG00000022119.15	2986.386	0.107	0.033	3.206	0.001344242	0.013013251	Rbm26	protein_coding
ENSMUSG00000025059.16	1440.527	0.129	0.040	3.206	0.001344352	0.013013251	Gk	protein_coding
ENSMUSG00000002342.17	1460.558	-0.158	0.049	-3.204	0.001354686	0.0130953	Tmem161a	protein_coding
ENSMUSG00000049659.15	5001.066	0.111	0.035	3.204	0.001356068	0.013099675	Aftph	protein_coding
ENSMUSG00000045210.8	4305.115	0.095	0.030	3.202	0.001364134	0.013168566	Vcpip1	protein_coding
ENSMUSG00000054737.11	755.876	0.166	0.052	3.202	0.001365578	0.0131705	Zfp182	protein_coding
ENSMUSG00000022240.9	10594.049	-0.101	0.032	-3.202	0.001366203	0.0131705	Ctnnd2	protein_coding
ENSMUSG00000061689.15	15796.965	-0.117	0.037	-3.201	0.001370406	0.013201984	Dlgap4	protein_coding
ENSMUSG00000028676.17	3582.685	0.115	0.036	3.199	0.001379168	0.013277314	Srsf10	protein_coding
ENSMUSG00000034653.11	2352.634	0.127	0.040	3.198	0.001384598	0.013320492	Ythdc2	protein_coding
ENSMUSG00000028034.15	7704.143	0.090	0.028	3.197	0.001386512	0.013329809	Fubp1	protein_coding
ENSMUSG00000022490.6	2260.824	-0.140	0.044	-3.196	0.001394811	0.01340045	Ppp1r1a	protein_coding
ENSMUSG00000034297.14	4542.876	0.116	0.036	3.195	0.001398469	0.01341179	Med13	protein_coding
ENSMUSG00000066798.3	814.080	0.173	0.054	3.195	0.001398689	0.01341179	Zbtb6	protein_coding
ENSMUSG00000092486.1	288.292	0.221	0.069	3.195	0.001399077	0.01341179	2610524H06Rik	protein_coding
ENSMUSG00000021704.7	2964.747	0.103	0.032	3.195	0.001399797	0.01341179	Mtx3	protein_coding
ENSMUSG00000050822.11	358.190	-0.243	0.076	-3.192	0.001411448	0.013509196	Slc29a4	protein_coding
ENSMUSG00000002980.14	726.290	-0.210	0.066	-3.192	0.001411881	0.013509196	Bcam	protein_coding
ENSMUSG00000033342.13	2345.788	0.114	0.036	3.188	0.001432329	0.013689355	Plppr5	protein_coding
ENSMUSG00000020949.9	4557.417	0.105	0.033	3.188	0.001432652	0.013689355	Fkbp3	protein_coding
ENSMUSG00000022307.15	15880.246	0.091	0.028	3.187	0.001435099	0.013703448	Oxr1	protein_coding
ENSMUSG00000021068.16	5199.583	0.118	0.037	3.187	0.001437177	0.013713998	Nin	protein_coding
ENSMUSG00000036202.15	1965.542	0.115	0.036	3.186	0.001441559	0.013737866	Rif1	protein_coding
ENSMUSG00000034154.15	2088.869	0.111	0.035	3.186	0.001441628	0.013737866	Ino80	protein_coding
ENSMUSG00000025993.10	543.093	0.216	0.068	3.185	0.001447043	0.013780148	Slc40a1	protein_coding
ENSMUSG00000054942.13	5935.442	0.092	0.029	3.184	0.001450629	0.013800101	Miga1	protein_coding

ENSMUSG00000023010.14	13319.872	-0.077	0.024	-3.184	0.001451096	0.013800101	Tmbim6	protein_coding
ENSMUSG00000064065.15	6992.609	0.102	0.032	3.183	0.001458973	0.013856307	Ipcef1	protein_coding
ENSMUSG00000027459.16	262.946	-0.237	0.075	-3.182	0.001462978	0.013884996	Fam110a	protein_coding
ENSMUSG00000045538.6	524.597	-0.189	0.059	-3.182	0.00146458	0.013890839	Ddx28	protein_coding
ENSMUSG00000036887.5	1424.052	-0.147	0.046	-3.180	0.001471136	0.013943033	C1qa	protein_coding
ENSMUSG00000021226.7	240.802	-0.237	0.074	-3.180	0.001472061	0.013943033	Acot2	protein_coding
ENSMUSG00000027900.15	974.332	0.158	0.050	3.180	0.001473356	0.013945926	Dram2	protein_coding
ENSMUSG00000054162.15	3467.321	0.163	0.051	3.179	0.001477668	0.013977351	Spock3	protein_coding
ENSMUSG00000031333.7	2188.897	0.110	0.035	3.177	0.001487104	0.014057174	Abcb7	protein_coding
ENSMUSG00000087370.3	3783.515	0.144	0.045	3.177	0.001488924	0.014064944	Tmem170b	protein_coding
ENSMUSG00000037138.17	3870.592	-0.102	0.032	-3.176	0.001491173	0.014076754	Aff3	protein_coding
ENSMUSG00000025949.16	3455.633	0.092	0.029	3.176	0.001495106	0.014104433	Pikfyve	protein_coding
ENSMUSG00000024112.16	6634.118	-0.161	0.051	-3.175	0.001500171	0.014142748	Cacna1h	protein_coding
ENSMUSG00000072501.4	3698.171	0.119	0.037	3.174	0.001502066	0.014151145	Phf20l1	protein_coding
ENSMUSG00000053929.16	6660.372	-0.078	0.025	-3.173	0.001510194	0.014208725	Cyhr1	protein_coding
ENSMUSG00000044365.15	2164.088	0.114	0.036	3.171	0.001516542	0.014258933	Cxxc4	protein_coding
ENSMUSG00000039704.6	2477.362	0.107	0.034	3.169	0.001527699	0.014348442	Lmbrd2	protein_coding
ENSMUSG00000048388.3	21205.677	0.102	0.032	3.169	0.001528098	0.014348442	Fam171b	protein_coding
ENSMUSG00000059890.16	4402.156	0.096	0.030	3.169	0.001530878	0.014364973	Ube4a	protein_coding
ENSMUSG00000028657.14	5154.742	0.094	0.030	3.168	0.00153702	0.014413014	Ppt1	protein_coding
ENSMUSG00000001750.15	1037.739	-0.155	0.049	-3.167	0.001541289	0.014443436	Tcirg1	protein_coding
ENSMUSG00000026159.13	5669.656	0.093	0.029	3.166	0.001545988	0.01447784	Agfg1	protein_coding
ENSMUSG00000019856.14	1660.072	0.163	0.052	3.165	0.001550176	0.014507423	Fam184a	protein_coding
ENSMUSG00000035007.5	2906.173	-0.107	0.034	-3.165	0.001552752	0.014521885	Rundc1	protein_coding
ENSMUSG00000021494.8	2258.826	-0.110	0.035	-3.164	0.001553898	0.014522965	Ddx41	protein_coding
ENSMUSG00000030878.11	637.753	-0.184	0.058	-3.164	0.001554981	0.014523465	Cdr2	protein_coding
ENSMUSG00000029516.19	9066.984	-0.117	0.037	-3.162	0.001566851	0.014624634	Cit	protein_coding
ENSMUSG00000006575.14	14526.732	-0.116	0.037	-3.161	0.00156979	0.014642373	Rundc3a	protein_coding
ENSMUSG00000020836.15	3065.815	-0.128	0.041	-3.159	0.00158417	0.014766726	Coro6	protein_coding
ENSMUSG00000035513.19	1056.523	-0.189	0.060	-3.158	0.001586156	0.014775467	Ntng2	protein_coding
ENSMUSG00000060301.6	1702.396	0.124	0.039	3.156	0.001597592	0.014872165	2610008E11Rik	protein_coding
ENSMUSG00000025158.7	3045.088	-0.134	0.042	-3.156	0.001600936	0.014893457	Rfng	protein_coding
ENSMUSG00000029359.13	1229.718	-0.185	0.059	-3.154	0.001611436	0.014981256	Tesc	protein_coding

ENSMUSG00000050587.14	5701.233	0.101	0.032	3.152	0.001622717	0.015076186	Lrrc4c	protein_coding
ENSMUSG00000025731.15	799.947	-0.193	0.061	-3.151	0.001625744	0.015093818	Mettl26	protein_coding
ENSMUSG00000028465.16	3626.520	-0.132	0.042	-3.151	0.001627405	0.015093818	Tln1	protein_coding
ENSMUSG00000031343.13	8450.417	0.093	0.030	3.151	0.001628328	0.015093818	Gabra3	protein_coding
ENSMUSG00000073563.2	2982.742	0.098	0.031	3.151	0.001629513	0.015093818	Csnk1g3	protein_coding
ENSMUSG00000028382.15	1249.187	0.158	0.050	3.150	0.00162997	0.015093818	Ptbp3	protein_coding
ENSMUSG00000035206.10	2559.734	-0.137	0.044	-3.150	0.001632945	0.015111444	Sppl2b	protein_coding
ENSMUSG00000026728.9	2018.692	-0.191	0.061	-3.150	0.001634475	0.015115676	Vim	protein_coding
ENSMUSG00000060126.14	8546.325	-0.150	0.048	-3.149	0.001636845	0.015127666	Tpt1	protein_coding
ENSMUSG00000008683.16	6385.038	0.081	0.026	3.149	0.001639494	0.015142216	Rps15a	protein_coding
ENSMUSG00000020205.8	862.068	-0.176	0.056	-3.147	0.00164857	0.015216076	Phlda1	protein_coding
ENSMUSG00000031729.6	5618.037	0.095	0.030	3.146	0.001652701	0.015244218	Ist1	protein_coding
ENSMUSG00000054976.14	2215.629	0.145	0.046	3.146	0.001655313	0.015258328	Nyap2	protein_coding
ENSMUSG00000049252.17	4318.109	0.155	0.049	3.145	0.001663276	0.015321711	Lrp1b	protein_coding
ENSMUSG00000022884.14	26994.710	0.083	0.027	3.144	0.001664929	0.015326917	Eif4a2	protein_coding
ENSMUSG00000022760.15	830.444	-0.187	0.059	-3.143	0.001672566	0.015387169	Thap7	protein_coding
ENSMUSG00000032737.13	2278.939	-0.130	0.041	-3.140	0.001691813	0.015554086	Inppl1	protein_coding
ENSMUSG00000051062.6	1090.086	-0.165	0.053	-3.134	0.001724812	0.01584713	Fbll1	protein_coding
ENSMUSG00000008575.17	4789.824	0.105	0.034	3.133	0.001728109	0.015867081	Nfib	protein_coding
ENSMUSG00000052040.10	9479.757	-0.080	0.026	-3.133	0.001729726	0.01587159	Klf13	protein_coding
ENSMUSG00000031591.14	3161.398	0.102	0.033	3.133	0.001732631	0.015887901	Asah1	protein_coding
ENSMUSG00000044147.8	898.342	-0.190	0.061	-3.132	0.001735842	0.015906994	Arf6	protein_coding
ENSMUSG00000043670.4	5540.276	-0.113	0.036	-3.131	0.001743691	0.015968536	Diras1	protein_coding
ENSMUSG00000002910.11	636.054	-0.210	0.067	-3.129	0.001751567	0.016030254	Arrdc2	protein_coding
ENSMUSG00000037685.15	16500.266	0.084	0.027	3.129	0.001756823	0.016067921	Atp8a1	protein_coding
ENSMUSG00000024924.14	4599.902	0.094	0.030	3.126	0.001773924	0.016213801	Vldlr	protein_coding
ENSMUSG00000025171.1	342.850	-0.208	0.066	-3.125	0.001775184	0.016214804	Ubtd1	protein_coding
ENSMUSG00000000420.15	1901.030	0.115	0.037	3.125	0.001777889	0.016228999	Galnt1	protein_coding
ENSMUSG00000022391.15	13369.638	-0.068	0.022	-3.122	0.001793432	0.016360276	Rangap1	protein_coding
ENSMUSG00000004591.16	1625.558	0.125	0.040	3.122	0.001798365	0.016390308	Pkn2	protein_coding
ENSMUSG00000041930.7	460.537	-0.203	0.065	-3.122	0.00179905	0.016390308	Fam222a	protein_coding
ENSMUSG00000024908.14	4768.656	0.092	0.029	3.120	0.001805472	0.016438195	Ppp6r3	protein_coding
ENSMUSG00000006651.8	57322.095	-0.112	0.036	-3.120	0.00181073	0.016475424	Aplp1	protein_coding

ENSMUSG00000042323.16	3024.193	0.113	0.036	3.118	0.001817993	0.016530835	Pbrm1	protein_coding
ENSMUSG00000066037.14	5069.949	0.108	0.035	3.117	0.001824343	0.016577878	Hnrnpr	protein_coding
ENSMUSG00000031451.6	3746.138	-0.115	0.037	-3.116	0.001831739	0.016634363	Gas6	protein_coding
ENSMUSG00000045136.6	4269.495	-0.136	0.044	-3.116	0.001834055	0.016635021	Tubb2b	protein_coding
ENSMUSG00000005442.13	12064.247	-0.119	0.038	-3.116	0.001834172	0.016635021	Cic	protein_coding
ENSMUSG00000039275.9	5285.538	-0.090	0.029	-3.114	0.00184317	0.016705878	Foxk2	protein_coding
ENSMUSG00000052296.7	10285.219	-0.104	0.033	-3.113	0.001850117	0.016753009	Ppp6r1	protein_coding
ENSMUSG00000028538.12	2014.598	-0.124	0.040	-3.113	0.001850747	0.016753009	St3gal3	protein_coding
ENSMUSG00000032030.16	3010.361	0.118	0.038	3.113	0.001852007	0.016753646	Cul5	protein_coding
ENSMUSG00000028747.10	216.931	-0.227	0.073	-3.112	0.001860288	0.01681777	Htr6	protein_coding
ENSMUSG00000034480.18	1439.452	0.134	0.043	3.111	0.001862143	0.016823749	Diaph2	protein_coding
ENSMUSG00000061306.15	3574.673	-0.105	0.034	-3.111	0.001864407	0.01683341	Slc38a10	protein_coding
ENSMUSG00000029344.14	823.111	-0.168	0.054	-3.110	0.00186772	0.016848737	Tpst2	protein_coding
ENSMUSG00000031486.15	1036.728	-0.165	0.053	-3.110	0.001868495	0.016848737	Adgra2	protein_coding
ENSMUSG00000046711.15	1186.150	-0.145	0.047	-3.109	0.001877607	0.016920071	Hmga1	protein_coding
ENSMUSG00000000902.13	2814.029	-0.111	0.036	-3.108	0.001881497	0.016935965	Smarcb1	protein_coding
ENSMUSG00000019852.7	10251.713	0.074	0.024	3.108	0.001882601	0.016935965	Arfgef3	protein_coding
ENSMUSG00000028670.14	3152.069	-0.150	0.048	-3.108	0.001882976	0.016935965	Lypla2	protein_coding
ENSMUSG00000017677.11	5274.898	0.121	0.039	3.108	0.001885523	0.016948058	Wsb1	protein_coding
ENSMUSG00000020463.15	2433.737	0.133	0.043	3.107	0.001886945	0.016950032	Ppp4r3b	protein_coding
ENSMUSG00000034593.16	34855.551	0.079	0.026	3.107	0.001892328	0.016984954	Myo5a	protein_coding
ENSMUSG00000028911.16	3089.587	-0.103	0.033	-3.106	0.001894007	0.016984954	Srsf4	protein_coding
ENSMUSG00000032913.13	2355.151	0.111	0.036	3.106	0.001894448	0.016984954	Lrig2	protein_coding
ENSMUSG00000020866.17	9702.261	-0.140	0.045	-3.106	0.00189846	0.017010103	Cacna1g	protein_coding
ENSMUSG00000021302.10	2796.741	0.132	0.043	3.105	0.001904887	0.017056844	Ggps1	protein_coding
ENSMUSG00000025823.9	3072.387	-0.138	0.044	-3.104	0.001910209	0.017090395	Pdia4	protein_coding
ENSMUSG00000093930.1	13245.928	0.103	0.033	3.104	0.001911211	0.017090395	Hmgcs1	protein_coding
ENSMUSG00000046985.11	2546.388	0.103	0.033	3.104	0.001912271	0.017090395	Tapt1	protein_coding
ENSMUSG00000000355.13	1609.907	0.132	0.042	3.103	0.001914319	0.017097853	Mcts1	protein_coding
ENSMUSG00000029608.10	28222.538	-0.098	0.032	-3.103	0.001916738	0.017108615	Rph3a	protein_coding
ENSMUSG00000026883.17	7739.318	-0.124	0.040	-3.100	0.001935263	0.017252119	Dab2ip	protein_coding
ENSMUSG00000026028.12	11084.594	0.089	0.029	3.099	0.001940477	0.017287667	Trak2	protein_coding
ENSMUSG00000022054.11	7336.520	-0.160	0.051	-3.098	0.001947636	0.017340479	Nefm	protein_coding

ENSMUSG00000042063.11	785.545	0.153	0.050	3.098	0.001948929	0.017341037	Zfp386	protein_coding
ENSMUSG00000033249.10	826.873	-0.179	0.058	-3.098	0.001951264	0.017350858	Hsf4	protein_coding
ENSMUSG00000026594.14	2487.217	0.110	0.035	3.095	0.001970326	0.017496816	Ralgps2	protein_coding
ENSMUSG00000040414.7	1387.129	-0.126	0.041	-3.095	0.001971265	0.017496816	Slc25a28	protein_coding
ENSMUSG00000029406.15	18972.160	-0.120	0.039	-3.094	0.001972426	0.017496816	Pitpnm2	protein_coding
ENSMUSG00000032966.14	18962.860	-0.096	0.031	-3.094	0.001972644	0.017496816	Fkbp1a	protein_coding
ENSMUSG00000070709.12	490.840	0.196	0.063	3.093	0.001984738	0.017591033	Zfp974	protein_coding
ENSMUSG00000035172.15	520.123	-0.182	0.059	-3.092	0.001985762	0.017591033	Plekhh3	protein_coding
ENSMUSG00000034160.13	23360.071	0.068	0.022	3.092	0.001987704	0.017597174	Ogt	protein_coding
ENSMUSG00000019146.2	3840.417	-0.104	0.034	-3.092	0.001989165	0.017599052	Cacng2	protein_coding
ENSMUSG00000040811.15	8066.826	-0.112	0.036	-3.091	0.001997348	0.017660366	Eml2	protein_coding
ENSMUSG00000018068.13	1445.517	0.129	0.042	3.088	0.002013871	0.017795297	Ints2	protein_coding
ENSMUSG00000025892.16	5004.834	0.097	0.031	3.087	0.002020053	0.01783874	Gria4	protein_coding
ENSMUSG00000030861.15	2932.393	0.102	0.033	3.087	0.002024775	0.017869245	Acadsb	protein_coding
ENSMUSG00000020650.15	2199.452	0.119	0.039	3.086	0.002026117	0.017869894	Bcap29	protein_coding
ENSMUSG00000070509.15	2119.425	-0.136	0.044	-3.086	0.002030679	0.017898926	Rgma	protein_coding
ENSMUSG00000020305.13	809.235	0.170	0.055	3.085	0.002033927	0.017903052	Asb3	protein_coding
ENSMUSG00000030595.15	889.640	-0.182	0.059	-3.085	0.002034614	0.017903052	Nfkbib	protein_coding
ENSMUSG00000034037.14	771.798	-0.208	0.068	-3.085	0.002034957	0.017903052	Fgd5	protein_coding
ENSMUSG00000030774.13	20460.957	0.083	0.027	3.085	0.002036561	0.017905982	Pak1	protein_coding
ENSMUSG00000010054.4	3373.730	-0.104	0.034	-3.082	0.002058254	0.018080479	Tusc2	protein_coding
ENSMUSG00000027203.15	724.529	-0.173	0.056	-3.082	0.002058973	0.018080479	Dut	protein_coding
ENSMUSG00000022176.11	406.252	-0.220	0.071	-3.080	0.002066791	0.018137824	Rem2	protein_coding
ENSMUSG00000036295.5	3100.128	0.117	0.038	3.080	0.00207103	0.018163718	Lrrn3	protein_coding
ENSMUSG00000027544.16	927.360	-0.153	0.050	-3.080	0.002072687	0.018166944	Nfatc2	protein_coding
ENSMUSG00000041609.16	3776.343	-0.114	0.037	-3.079	0.002076616	0.018190075	Bicdl1	protein_coding
ENSMUSG00000037643.14	3082.726	0.103	0.033	3.078	0.002080861	0.018215931	Prkci	protein_coding
ENSMUSG00000023915.4	4185.831	-0.111	0.036	-3.078	0.002087118	0.018258393	Tnfrsf21	protein_coding
ENSMUSG00000025060.14	5397.162	0.092	0.030	3.077	0.002089472	0.018258393	Slk	protein_coding
ENSMUSG00000025016.10	8262.641	0.091	0.030	3.077	0.002089598	0.018258393	Tm9sf3	protein_coding
ENSMUSG00000020811.16	4170.770	-0.117	0.038	-3.076	0.002095539	0.01829896	Wscd1	protein_coding
ENSMUSG00000028445.7	1571.805	-0.180	0.058	-3.075	0.00210303	0.018353009	Enho	protein_coding
ENSMUSG00000027637.3	1202.630	-0.135	0.044	-3.075	0.002107344	0.018376893	1110008F13Rik	protein_coding

ENSMUSG00000001525.10	25815.111	-0.095	0.031	-3.075	0.002108375	0.018376893	Tubb5	protein_coding
ENSMUSG00000045333.15	947.121	-0.197	0.064	-3.074	0.002110179	0.018381251	Zfp423	protein_coding
ENSMUSG00000040536.15	6630.909	0.109	0.035	3.074	0.00211253	0.01839036	Necab1	protein_coding
ENSMUSG00000050705.15	2191.961	-0.117	0.038	-3.073	0.002117915	0.018425862	2310061I04Rik	protein_coding
ENSMUSG00000020868.15	1337.446	-0.136	0.044	-3.072	0.002122833	0.018457257	Xylt2	protein_coding
ENSMUSG00000034656.17	9972.353	-0.128	0.042	-3.072	0.002126837	0.018471794	Cacna1a	protein_coding
ENSMUSG00000052459.13	27929.577	0.082	0.027	3.072	0.002128273	0.018471794	Atp6v1a	protein_coding
ENSMUSG00000006362.16	2195.179	-0.159	0.052	-3.071	0.00213109	0.018471794	Cbfa2t3	protein_coding
ENSMUSG00000036898.16	1348.430	0.136	0.044	3.071	0.002135882	0.018471794	Zfp157	protein_coding
ENSMUSG00000032875.8	11778.126	-0.116	0.038	-3.071	0.002136296	0.018471794	Arhgef17	protein_coding
ENSMUSG00000013878.18	1250.670	0.144	0.047	3.071	0.002136531	0.018471794	Rnf170	protein_coding
ENSMUSG00000022099.16	16684.674	-0.095	0.031	-3.071	0.002136572	0.018471794	Dmtn	protein_coding
ENSMUSG00000036957.8	1249.380	-0.171	0.056	-3.070	0.002137205	0.018471794	Lrfn3	protein_coding
ENSMUSG00000034684.12	574.760	-0.193	0.063	-3.070	0.002138155	0.018471794	Sema3f	protein_coding
ENSMUSG00000041629.7	1294.135	-0.151	0.049	-3.070	0.002138855	0.018471794	Fam104a	protein_coding
ENSMUSG00000022305.12	2067.776	0.133	0.043	3.070	0.002138922	0.018471794	Lrp12	protein_coding
ENSMUSG00000030410.16	6307.371	-0.106	0.035	-3.070	0.002142002	0.018487065	Dmwd	protein_coding
ENSMUSG00000034789.7	2171.990	-0.118	0.039	-3.069	0.00214441	0.018496524	Rab24	protein_coding
ENSMUSG00000046111.17	2048.140	0.110	0.036	3.069	0.002147294	0.018510067	Cep295	protein_coding
ENSMUSG00000026275.13	5571.158	0.089	0.029	3.067	0.002162705	0.018631523	Ppp1r7	protein_coding
ENSMUSG00000035835.14	3654.829	-0.150	0.049	-3.066	0.002167509	0.018661498	Plppr3	protein_coding
ENSMUSG00000031176.8	5495.761	0.100	0.033	3.065	0.00217446	0.018707934	Dynlt3	protein_coding
ENSMUSG00000020440.13	3362.578	-0.142	0.046	-3.065	0.002175557	0.018707934	Arf5	protein_coding
ENSMUSG00000003929.10	1299.587	0.149	0.049	3.065	0.002179312	0.018728794	Zfp81	protein_coding
ENSMUSG00000028444.17	3482.437	-0.119	0.039	-3.064	0.002183534	0.018753645	Cntfr	protein_coding
ENSMUSG00000050830.17	399.358	-0.201	0.066	-3.063	0.002193106	0.018824381	Vwc2	protein_coding
ENSMUSG00000072825.11	18244.681	-0.120	0.039	-3.063	0.002194664	0.018826292	Cep170b	protein_coding
ENSMUSG00000039789.7	1238.902	0.134	0.044	3.061	0.002205487	0.018907624	Zfp597	protein_coding
ENSMUSG00000025875.14	1751.518	-0.142	0.047	-3.059	0.002221177	0.01903056	Tspan17	protein_coding
ENSMUSG00000003184.15	1381.303	-0.144	0.047	-3.058	0.002226506	0.019064629	Irf3	protein_coding
ENSMUSG00000015222.17	69761.750	0.082	0.027	3.058	0.002230129	0.019084056	Map2	protein_coding
ENSMUSG00000052214.9	3306.462	-0.102	0.033	-3.057	0.002233723	0.01909272	Opa3	protein_coding
ENSMUSG00000027030.15	3311.872	0.100	0.033	3.057	0.002233851	0.01909272	Stk39	protein_coding

ENSMUSG00000002108.10	159.195	-0.233	0.076	-3.056	0.002240778	0.019131402	Nr1h3	protein_coding
ENSMUSG00000021071.16	19254.231	-0.078	0.026	-3.056	0.002241092	0.019131402	Trim9	protein_coding
ENSMUSG00000017774.19	1355.492	-0.127	0.042	-3.055	0.002248733	0.019176227	Myo1c	protein_coding
ENSMUSG00000019471.10	5407.596	-0.095	0.031	-3.055	0.002249064	0.019176227	Cdc37	protein_coding
ENSMUSG00000009075.2	1417.401	-0.145	0.048	-3.055	0.002252391	0.019192984	Cabp7	protein_coding
ENSMUSG00000072946.12	1647.531	0.125	0.041	3.054	0.002256223	0.019214022	Ptgr2	protein_coding
ENSMUSG00000030557.17	7978.722	0.102	0.033	3.054	0.002259022	0.019226241	Mef2a	protein_coding
ENSMUSG00000028289.12	3497.949	0.152	0.050	3.052	0.002270438	0.019311744	Epha7	protein_coding
ENSMUSG00000043964.14	645.103	-0.167	0.055	-3.051	0.00227951	0.019365527	Orai3	protein_coding
ENSMUSG00000024163.17	32535.428	-0.084	0.028	-3.051	0.002281852	0.019371937	Mapk8ip3	protein_coding
ENSMUSG00000024217.9	769.455	-0.160	0.052	-3.051	0.002283013	0.019371937	Snrpc	protein_coding
ENSMUSG00000026259.14	15333.253	-0.089	0.029	-3.050	0.002284704	0.019374616	Ngef	protein_coding
ENSMUSG00000005583.16	27198.322	0.126	0.041	3.048	0.002304909	0.019524923	Mef2c	protein_coding
ENSMUSG00000054693.14	4663.605	0.102	0.034	3.048	0.002305199	0.019524923	Adam10	protein_coding
ENSMUSG00000027366.12	2261.430	0.112	0.037	3.048	0.002307203	0.019530164	Sppl2a	protein_coding
ENSMUSG00000030604.9	675.464	0.173	0.057	3.047	0.002310036	0.019537894	Zfp626	protein_coding
ENSMUSG00000039089.15	1723.868	-0.129	0.042	-3.047	0.002310889	0.019537894	L3mbtl3	protein_coding
ENSMUSG00000049321.17	1326.397	0.157	0.052	3.045	0.002330115	0.019688633	Zfp2	protein_coding
ENSMUSG00000045087.8	441.256	-0.225	0.074	-3.041	0.002355771	0.019892317	S1pr5	protein_coding
ENSMUSG00000054793.8	10867.068	-0.093	0.030	-3.041	0.002357043	0.019892317	Cadm4	protein_coding
ENSMUSG00000032184.5	1520.814	-0.150	0.049	-3.041	0.002360453	0.019909173	Lysmd2	protein_coding
ENSMUSG00000022556.9	2655.307	-0.139	0.046	-3.040	0.002362894	0.019909766	Hsf1	protein_coding
ENSMUSG00000020590.16	4527.788	0.089	0.029	3.040	0.002363349	0.019909766	Snx13	protein_coding
ENSMUSG00000035623.14	3477.318	0.100	0.033	3.039	0.002375135	0.019985165	Rsf1	protein_coding
ENSMUSG00000005802.12	3646.402	0.128	0.042	3.039	0.002376731	0.019986661	Slc30a4	protein_coding
ENSMUSG00000036093.7	3868.096	0.090	0.030	3.038	0.002383328	0.020030184	Arl5a	protein_coding
ENSMUSG00000029475.17	1275.278	-0.134	0.044	-3.037	0.002385877	0.020039664	Kdm2b	protein_coding
ENSMUSG00000041168.9	4287.412	-0.093	0.031	-3.037	0.002393344	0.020090408	Lonp1	protein_coding
ENSMUSG00000031217.8	822.786	-0.187	0.062	-3.036	0.002396866	0.020107991	Efnb1	protein_coding
ENSMUSG00000021476.9	10185.776	-0.072	0.024	-3.036	0.002400147	0.020123538	Habp4	protein_coding
ENSMUSG00000028568.16	1398.101	0.135	0.044	3.035	0.002402325	0.020129823	Btf3l4	protein_coding
ENSMUSG00000001829.17	3283.329	-0.093	0.031	-3.034	0.002413163	0.020208628	Clpb	protein_coding
ENSMUSG00000028211.11	953.452	0.147	0.048	3.033	0.002418527	0.020241517	Trp53inp1	protein_coding

ENSMUSG00000024953.16	5501.191	-0.089	0.029	-3.031	0.002433792	0.020357193	Prdx5	protein_coding
ENSMUSG00000022329.14	602.845	0.165	0.055	3.031	0.002435803	0.02036193	Stk3	protein_coding
ENSMUSG00000019888.16	484.961	0.205	0.068	3.031	0.002441078	0.020393928	Mgat4c	protein_coding
ENSMUSG00000029265.4	1839.851	0.118	0.039	3.029	0.002452264	0.020475239	Dr1	protein_coding
ENSMUSG00000025579.14	23494.737	-0.140	0.046	-3.028	0.002459884	0.020520028	Gaa	protein_coding
ENSMUSG00000024947.16	3190.785	-0.110	0.036	-3.028	0.00246054	0.020520028	Men1	protein_coding
ENSMUSG00000059005.13	7946.507	0.083	0.027	3.028	0.002462326	0.020522783	Hnrnpa3	protein_coding
ENSMUSG00000027434.11	157.344	-0.233	0.077	-3.026	0.002475159	0.02061755	Nkx2-2	protein_coding
ENSMUSG00000028917.14	5411.116	-0.092	0.030	-3.026	0.002477821	0.020627532	Plekhm2	protein_coding
ENSMUSG00000000197.7	6805.936	0.101	0.034	3.025	0.002482582	0.020654968	Nalcn	protein_coding
ENSMUSG00000034998.18	612.856	0.174	0.057	3.022	0.002507581	0.020850317	Foxn2	protein_coding
ENSMUSG00000050310.8	4076.484	0.100	0.033	3.022	0.002509601	0.020850317	Rictor	protein_coding
ENSMUSG00000029223.12	23107.798	-0.083	0.027	-3.022	0.0025105	0.020850317	Uchl1	protein_coding
ENSMUSG00000025511.14	379.054	-0.205	0.068	-3.022	0.002512353	0.020853416	Tspan4	protein_coding
ENSMUSG00000018417.14	1410.640	0.150	0.050	3.021	0.002518354	0.020890921	Myo1b	protein_coding
ENSMUSG00000052534.15	10634.226	0.111	0.037	3.019	0.002538114	0.021042462	Pbx1	protein_coding
ENSMUSG00000034040.16	2885.020	-0.102	0.034	-3.018	0.002542304	0.021064803	Wbscr17	protein_coding
ENSMUSG00000021413.9	7240.185	0.098	0.032	3.018	0.002547481	0.021095295	Prpf4b	protein_coding
ENSMUSG00000034936.2	861.964	-0.168	0.056	-3.017	0.00254913	0.021096554	Arl4d	protein_coding
ENSMUSG00000033152.13	6793.555	-0.116	0.039	-3.014	0.002580683	0.021345154	Podxl2	protein_coding
ENSMUSG00000000552.9	2916.151	-0.152	0.050	-3.013	0.002583867	0.021358957	Zfp385a	protein_coding
ENSMUSG00000052934.14	6915.708	-0.097	0.032	-3.009	0.002623033	0.02165467	Fbxo31	protein_coding
ENSMUSG00000068154.5	216.802	-0.226	0.075	-3.009	0.00262425	0.02165467	Insm1	protein_coding
ENSMUSG00000078137.1	2646.828	-0.162	0.054	-3.008	0.002626718	0.02166235	Ankrd63	protein_coding
ENSMUSG00000017747.13	406.008	-0.199	0.066	-3.008	0.002629571	0.021671781	Ghdc	protein_coding
ENSMUSG00000015749.12	4443.365	0.093	0.031	3.008	0.002630936	0.021671781	Anp32e	protein_coding
ENSMUSG00000041073.10	2830.326	-0.125	0.041	-3.008	0.002633845	0.021683069	Nacad	protein_coding
ENSMUSG00000002820.6	1386.867	-0.128	0.043	-3.007	0.002636034	0.021688416	Atg4d	protein_coding
ENSMUSG00000036097.7	4639.637	0.092	0.031	3.007	0.002638062	0.021692441	Fam178a	protein_coding
ENSMUSG00000003970.8	9386.655	-0.070	0.023	-3.006	0.002651398	0.021773239	Rpl8	protein_coding
ENSMUSG00000014426.8	2882.504	-0.097	0.032	-3.005	0.002652253	0.021773239	Map3k4	protein_coding
ENSMUSG00000027893.14	16834.084	0.068	0.023	3.005	0.002652522	0.021773239	Ahcyl1	protein_coding
ENSMUSG00000020589.17	12626.732	0.100	0.033	3.005	0.002654674	0.021778213	Fam49a	protein_coding

ENSMUSG00000021282.17	9602.115	0.080	0.027	3.005	0.002659678	0.021806578	Eif5	protein_coding
ENSMUSG00000031872.14	785.673	-0.176	0.059	-3.004	0.002666136	0.021846814	Bean1	protein_coding
ENSMUSG00000022865.13	2050.309	0.120	0.040	3.003	0.002676554	0.021908723	Cxadr	protein_coding
ENSMUSG00000004937.6	6029.400	-0.096	0.032	-3.003	0.002677614	0.021908723	Sgta	protein_coding
ENSMUSG00000028677.19	7524.714	-0.087	0.029	-3.002	0.002678355	0.021908723	Rnf220	protein_coding
ENSMUSG00000033096.7	2092.395	0.106	0.035	3.002	0.002684184	0.021930954	Apmap	protein_coding
ENSMUSG00000025423.15	3550.861	0.107	0.036	3.000	0.002696309	0.02201725	Pias2	protein_coding
ENSMUSG00000027220.2	11689.569	-0.078	0.026	-3.000	0.002699792	0.022032927	Syt13	protein_coding
ENSMUSG00000022389.14	24485.777	0.087	0.029	3.000	0.00270236	0.022041122	Tef	protein_coding
ENSMUSG00000037286.15	2612.804	0.108	0.036	3.000	0.002704011	0.022041836	Stag1	protein_coding
ENSMUSG00000063297.7	7726.271	0.147	0.049	2.999	0.002707601	0.022053298	Luzp2	protein_coding
ENSMUSG00000024937.14	1673.536	-0.124	0.041	-2.999	0.002709736	0.022053298	Ehbp1l1	protein_coding
ENSMUSG00000067942.5	955.772	0.170	0.057	2.999	0.002710112	0.022053298	Zfp160	protein_coding
ENSMUSG00000034902.17	24405.657	-0.124	0.041	-2.998	0.002713668	0.022069497	Pip5k1c	protein_coding
ENSMUSG00000035246.16	2845.405	0.096	0.032	2.998	0.002717922	0.022091344	Pcyt1b	protein_coding
ENSMUSG00000026585.13	14338.445	0.077	0.026	2.996	0.002731199	0.022183194	Kifap3	protein_coding
ENSMUSG00000029392.12	2482.236	-0.127	0.042	-2.996	0.00273237	0.022183194	Rilpl1	protein_coding
ENSMUSG00000026782.15	10478.011	0.092	0.031	2.995	0.002741619	0.022245472	Abi2	protein_coding
ENSMUSG00000026443.3	5850.729	-0.118	0.039	-2.993	0.002760328	0.022384389	Lrrn2	protein_coding
ENSMUSG00000030035.14	1146.721	-0.140	0.047	-2.992	0.002775717	0.02249624	Wbp1	protein_coding
ENSMUSG00000063052.9	1085.639	0.142	0.047	2.990	0.002786152	0.022563601	Lrrc40	protein_coding
ENSMUSG00000063605.5	54.709	-0.219	0.073	-2.990	0.002786456	NA	Ccdc102a	protein_coding
ENSMUSG00000029404.15	1114.516	-0.144	0.048	-2.990	0.002787231	0.022563601	Arl6ip4	protein_coding
ENSMUSG00000029416.17	1174.106	-0.154	0.051	-2.989	0.002796548	0.022626028	Slc15a4	protein_coding
ENSMUSG00000064061.13	5365.475	0.095	0.032	2.987	0.002818654	0.0227918	Dzip3	protein_coding
ENSMUSG00000051391.9	54193.219	-0.074	0.025	-2.985	0.002831893	0.022873863	Ywhag	protein_coding
ENSMUSG00000034412.13	1305.701	-0.137	0.046	-2.985	0.002832048	0.022873863	Tbc1d10a	protein_coding
ENSMUSG00000009628.14	59.235	0.222	0.074	2.985	0.002832051	NA	Tex15	protein_coding
ENSMUSG00000028541.14	3394.699	-0.119	0.040	-2.984	0.002840989	0.022932929	B4galt2	protein_coding
ENSMUSG00000028484.16	9914.699	0.082	0.028	2.983	0.002854188	0.023026285	Psip1	protein_coding
ENSMUSG00000031647.10	2827.961	0.126	0.042	2.983	0.002857236	0.023037691	Mfap3l	protein_coding
ENSMUSG00000038594.9	1293.424	0.122	0.041	2.982	0.002867	0.023085463	Cep85l	protein_coding
ENSMUSG00000015087.14	8396.827	-0.077	0.026	-2.982	0.002867864	0.023085463	Rabl6	protein_coding

ENSMUSG00000033256.14	1188.943	-0.132	0.044	-2.982	0.002868075	0.023085463	Shf	protein_coding
ENSMUSG00000051243.14	4117.812	-0.136	0.045	-2.981	0.002869952	0.023087387	Islr2	protein_coding
ENSMUSG00000037972.6	8061.827	0.084	0.028	2.981	0.002875158	0.023116071	Snn	protein_coding
ENSMUSG00000026020.9	2015.834	0.117	0.039	2.977	0.002914499	0.023400905	Nop58	protein_coding
ENSMUSG00000062901.2	4053.071	0.109	0.037	2.977	0.002914576	0.023400905	Klhl24	protein_coding
ENSMUSG00000014850.15	1085.959	0.145	0.049	2.977	0.002915566	0.023400905	Msh3	protein_coding
ENSMUSG00000037166.5	330.219	-0.232	0.078	-2.976	0.002924018	0.023455381	Ppp1r14a	protein_coding
ENSMUSG00000003581.14	1540.026	-0.142	0.048	-2.974	0.002940465	0.023560982	Rnf215	protein_coding
ENSMUSG00000002222.14	7426.127	0.092	0.031	2.974	0.002940526	0.023560982	Rmnd5a	protein_coding
ENSMUSG00000063146.11	9410.584	-0.114	0.038	-2.974	0.002943478	0.023571238	Clip2	protein_coding
ENSMUSG00000026678.10	4103.300	0.121	0.041	2.972	0.002956914	0.023664692	Rgs5	protein_coding
ENSMUSG00000021076.6	5244.536	0.087	0.029	2.972	0.002958506	0.023664692	Actr10	protein_coding
ENSMUSG00000025968.16	6733.695	0.087	0.029	2.972	0.002960331	0.023665854	Ndufs1	protein_coding
ENSMUSG00000048988.8	2006.964	-0.158	0.053	-2.971	0.002967148	0.023706908	Elfn1	protein_coding
ENSMUSG00000008855.17	10957.776	-0.139	0.047	-2.971	0.002972506	0.023736259	Hdac5	protein_coding
ENSMUSG00000024268.15	26685.449	-0.089	0.030	-2.968	0.002993118	0.023873797	Celf4	protein_coding
ENSMUSG00000031805.17	439.281	-0.222	0.075	-2.967	0.003010441	0.023998394	Jak3	protein_coding
ENSMUSG00000038538.17	5138.697	0.098	0.033	2.966	0.003012865	0.024004137	Ubn2	protein_coding
ENSMUSG00000044807.13	2524.240	0.117	0.040	2.966	0.003019837	0.024046089	Zfp354c	protein_coding
ENSMUSG00000026203.16	4424.098	-0.104	0.035	-2.965	0.003025097	0.024063972	Dnajb2	protein_coding
ENSMUSG00000037533.19	2966.900	0.098	0.033	2.965	0.003025497	0.024063972	Rapgef6	protein_coding
ENSMUSG00000044667.12	11409.375	0.085	0.029	2.965	0.003027374	0.024065322	Plppr4	protein_coding
ENSMUSG00000013419.7	4639.140	-0.114	0.039	-2.965	0.00303076	0.024078657	Zfp651	protein_coding
ENSMUSG00000024759.13	2211.379	0.137	0.046	2.964	0.003035529	0.024102952	Atl3	protein_coding
ENSMUSG00000006456.10	1367.546	-0.140	0.047	-2.963	0.003048495	0.02419228	Rbm14	protein_coding
ENSMUSG00000043259.15	4062.159	0.097	0.033	2.961	0.003062487	0.024289641	Fam13c	protein_coding
ENSMUSG00000039233.12	3388.504	0.105	0.036	2.961	0.003066807	0.024307159	Tbce	protein_coding
ENSMUSG00000038805.10	272.478	-0.231	0.078	-2.961	0.003069018	0.024307159	Six3	protein_coding
ENSMUSG00000030751.18	3189.382	0.092	0.031	2.961	0.00306987	0.024307159	Psma1	protein_coding
ENSMUSG00000039000.8	4877.043	0.088	0.030	2.959	0.003081999	0.024378678	Ube3c	protein_coding
ENSMUSG00000071302.10	939.612	0.167	0.056	2.959	0.003082362	0.024378678	2610044O15Rik8	protein_coding
ENSMUSG00000030757.13	1787.015	0.130	0.044	2.959	0.003085593	0.024390545	Zkscan2	protein_coding
ENSMUSG00000062627.9	2165.648	0.121	0.041	2.958	0.003092187	0.024428972	Mysm1	protein_coding

ENSMUSG00000031706.7	2777.073	-0.145	0.049	-2.958	0.003096244	0.024447314	Rfx1	protein_coding
ENSMUSG00000020923.17	6791.025	-0.073	0.025	-2.957	0.003106394	0.024513727	Ubtf	protein_coding
ENSMUSG00000096606.2	177.647	-0.227	0.077	-2.956	0.003114249	0.024561963	Tpbgl	protein_coding
ENSMUSG00000020719.14	44186.496	0.079	0.027	2.956	0.003118123	0.024578763	Ddx5	protein_coding
ENSMUSG00000028207.18	3862.112	0.102	0.035	2.956	0.003121483	0.024591495	Asph	protein_coding
ENSMUSG00000031095.15	2138.040	0.143	0.048	2.954	0.003133669	0.024673703	Cul4b	protein_coding
ENSMUSG00000079157.4	3834.770	-0.132	0.045	-2.954	0.003139976	0.024709562	Fam155a	protein_coding
ENSMUSG00000002803.14	2315.856	-0.118	0.040	-2.954	0.00314173	0.024709566	Btbd6	protein_coding
ENSMUSG00000027580.17	627.529	-0.179	0.061	-2.953	0.003151999	0.024776509	Helz2	protein_coding
ENSMUSG00000018661.17	3447.199	-0.087	0.029	-2.952	0.003155231	0.024788086	Cog1	protein_coding
ENSMUSG00000001260.10	1321.826	0.155	0.052	2.952	0.003161423	0.024818092	Gabrg1	protein_coding
ENSMUSG00000044709.6	731.726	-0.158	0.053	-2.951	0.003162572	0.024818092	Gemin7	protein_coding
ENSMUSG00000015363.13	1101.142	-0.137	0.047	-2.951	0.003170458	0.024853316	Trabd	protein_coding
ENSMUSG00000074227.12	596.567	-0.218	0.074	-2.951	0.003170588	0.024853316	Spint2	protein_coding
ENSMUSG00000040021.13	2316.840	0.100	0.034	2.949	0.003182893	0.024935906	Lats1	protein_coding
ENSMUSG00000032977.9	1999.707	-0.132	0.045	-2.949	0.003188428	0.024965388	Fam207a	protein_coding
ENSMUSG00000027457.15	22056.889	-0.100	0.034	-2.948	0.00319418	0.024996544	Snph	protein_coding
ENSMUSG00000019699.16	8256.218	0.073	0.025	2.947	0.003206156	0.02507634	Akt3	protein_coding
ENSMUSG00000010529.6	102.059	-0.230	0.078	-2.947	0.003208969	0.025084418	Gm266	protein_coding
ENSMUSG00000044894.14	2396.720	-0.135	0.046	-2.945	0.003229642	0.025232023	Uqcrq	protein_coding
ENSMUSG00000021831.8	2363.176	0.101	0.034	2.945	0.003231738	0.025234414	Ero1l	protein_coding
ENSMUSG00000026885.13	1480.602	-0.119	0.040	-2.944	0.003243182	0.025302111	Ttll11	protein_coding
ENSMUSG00000037674.15	2769.055	0.106	0.036	2.944	0.003243998	0.025302111	Rfx7	protein_coding
ENSMUSG00000070934.6	3967.576	-0.097	0.033	-2.943	0.003254193	0.025367587	Rraga	protein_coding
ENSMUSG00000030854.17	14938.124	-0.114	0.039	-2.940	0.00328247	0.02557387	Ptpn5	protein_coding
ENSMUSG00000033726.8	353.572	-0.203	0.069	-2.940	0.003285065	0.02557995	Emx1	protein_coding
ENSMUSG00000035696.15	2944.225	0.121	0.041	2.938	0.003304227	0.02571495	Rnf38	protein_coding
ENSMUSG00000004895.9	1761.275	-0.118	0.040	-2.934	0.0033416	0.02599145	Prcc	protein_coding
ENSMUSG00000048490.13	2233.891	0.136	0.047	2.934	0.003346541	0.026013897	Nrip1	protein_coding
ENSMUSG00000029547.10	6976.670	-0.114	0.039	-2.934	0.003348177	0.026013897	Ints1	protein_coding
ENSMUSG00000061111.8	2330.486	-0.123	0.042	-2.933	0.003354452	0.026048289	Fam195b	protein_coding
ENSMUSG00000042256.4	203.995	0.218	0.074	2.932	0.003368861	0.026145773	Ptchd4	protein_coding
ENSMUSG00000029676.15	1022.906	0.148	0.050	2.931	0.003374558	0.026175576	Pot1a	protein_coding

ENSMUSG00000070823.6	2389.175	-0.111	0.038	-2.930	0.003387976	0.026258778	Gm1043	protein_coding
ENSMUSG00000009092.5	103.062	-0.227	0.078	-2.930	0.003389011	0.026258778	Derl3	protein_coding
ENSMUSG00000039715.8	1641.974	-0.141	0.048	-2.929	0.003399997	0.026329427	Wdr34	protein_coding
ENSMUSG00000022434.7	926.459	0.143	0.049	2.928	0.003411668	0.026405297	Fam118a	protein_coding
ENSMUSG00000022108.7	20290.390	-0.077	0.026	-2.928	0.003414487	0.026412612	Itm2b	protein_coding
ENSMUSG00000005779.12	4176.240	-0.092	0.032	-2.927	0.003417663	0.026422679	Psmb4	protein_coding
ENSMUSG00000017754.13	2463.737	-0.110	0.038	-2.927	0.003422757	0.026447555	Pltp	protein_coding
ENSMUSG00000040675.17	800.901	0.158	0.054	2.925	0.003441569	0.026568021	Mthfd1l	protein_coding
ENSMUSG00000009681.10	9052.838	-0.095	0.032	-2.925	0.003442118	0.026568021	Bcr	protein_coding
ENSMUSG00000002111.8	114.203	-0.227	0.077	-2.925	0.003448423	0.026596511	Spi1	protein_coding
ENSMUSG00000029726.16	1730.369	-0.122	0.042	-2.925	0.003449583	0.026596511	Mepce	protein_coding
ENSMUSG00000027981.14	1764.300	0.137	0.047	2.924	0.003453679	0.026613532	Rnpc3	protein_coding
ENSMUSG00000027312.14	13771.411	0.064	0.022	2.924	0.003458092	0.02663298	Atrn	protein_coding
ENSMUSG00000029998.14	5836.564	0.081	0.028	2.923	0.003461463	0.026644382	Pcyox1	protein_coding
ENSMUSG00000036377.18	4071.097	-0.121	0.041	-2.923	0.003463684	0.026646924	C530008M17Rik	protein_coding
ENSMUSG00000029152.13	12479.510	0.067	0.023	2.921	0.003484314	0.026791011	Ociad1	protein_coding
ENSMUSG00000029095.17	8259.541	-0.081	0.028	-2.921	0.003493682	0.026848393	Ablim2	protein_coding
ENSMUSG00000007613.15	1273.818	0.148	0.051	2.920	0.003503177	0.026906689	Tgfbr1	protein_coding
ENSMUSG00000021730.8	4343.334	0.085	0.029	2.919	0.003513564	0.026971772	Hcn1	protein_coding
ENSMUSG00000002812.4	4380.215	-0.084	0.029	-2.918	0.003521682	0.027019371	Flii	protein_coding
ENSMUSG00000046058.7	920.375	-0.182	0.063	-2.917	0.003531381	0.027061196	Eid2	protein_coding
ENSMUSG00000022124.14	4356.387	0.094	0.032	2.917	0.003532238	0.027061196	Fbxl3	protein_coding
ENSMUSG00000050628.6	292.103	-0.199	0.068	-2.917	0.003532893	0.027061196	Ubald2	protein_coding
ENSMUSG00000048787.13	1092.244	0.129	0.044	2.917	0.003537703	0.027083315	Dcun1d3	protein_coding
ENSMUSG00000040502.5	755.224	-0.159	0.054	-2.916	0.003544328	0.027119303	March9	protein_coding
ENSMUSG00000028645.11	4542.588	-0.121	0.042	-2.916	0.00354739	0.027128005	Slc2a1	protein_coding
ENSMUSG00000027546.15	19938.446	-0.084	0.029	-2.914	0.003573217	0.027310697	Atp9a	protein_coding
ENSMUSG00000024293.15	1036.715	0.131	0.045	2.913	0.003583334	0.027373174	Esco1	protein_coding
ENSMUSG00000022295.7	11430.140	0.073	0.025	2.912	0.003587046	0.027386687	Atp6v1c1	protein_coding
ENSMUSG00000039100.9	14363.913	0.080	0.027	2.911	0.003597267	0.027449851	March6	protein_coding
ENSMUSG00000017943.15	3486.246	-0.096	0.033	-2.911	0.003602073	0.027471655	Gdap1l1	protein_coding
ENSMUSG00000022562.14	1567.172	-0.135	0.046	-2.911	0.003606678	0.02748331	Oplah	protein_coding
ENSMUSG00000047669.8	429.224	-0.196	0.067	-2.911	0.003607501	0.02748331	Msl3l2	protein_coding

ENSMUSG00000061458.9	1047.613	0.143	0.049	2.909	0.003623654	0.027591456	Nol10	protein_coding
ENSMUSG00000024862.16	11500.407	-0.099	0.034	-2.909	0.003625651	0.027591752	Klc2	protein_coding
ENSMUSG00000005069.12	3618.804	-0.098	0.034	-2.909	0.003628131	0.027595722	Pex5	protein_coding
ENSMUSG00000037003.15	1247.349	-0.156	0.054	-2.906	0.003656008	0.027792759	Tns2	protein_coding
ENSMUSG00000021754.17	586.429	0.209	0.072	2.905	0.003673792	0.027912895	Map3k1	protein_coding
ENSMUSG00000030541.16	2148.486	-0.121	0.042	-2.902	0.003712711	0.02818808	Idh2	protein_coding
ENSMUSG00000059173.19	12566.060	0.108	0.037	2.901	0.003716475	0.02818808	Pde1a	protein_coding
ENSMUSG00000022961.17	27423.576	0.057	0.020	2.901	0.003717555	0.02818808	Son	protein_coding
ENSMUSG00000038812.16	517.784	-0.166	0.057	-2.901	0.003718011	0.02818808	Trmt112	protein_coding
ENSMUSG00000015882.17	1012.881	0.138	0.047	2.900	0.003733103	0.028287285	Lcorl	protein_coding
ENSMUSG00000055493.4	1579.358	0.136	0.047	2.899	0.003740647	0.028329217	Epm2a	protein_coding
ENSMUSG00000026004.15	692.207	0.178	0.061	2.899	0.003748208	0.02837124	Kansl1l	protein_coding
ENSMUSG00000007021.4	8637.358	-0.092	0.032	-2.898	0.003750662	0.028374571	Syngr3	protein_coding
ENSMUSG00000036641.8	798.561	0.148	0.051	2.897	0.003771061	0.028513593	Ccdc148	protein_coding
ENSMUSG00000002083.13	249.253	-0.209	0.072	-2.896	0.003779588	0.028562738	Bbc3	protein_coding
ENSMUSG00000021024.14	3100.554	0.100	0.035	2.893	0.003813635	0.028804596	Psma6	protein_coding
ENSMUSG00000074829.10	833.306	0.159	0.055	2.893	0.003816824	0.02881324	2010315B03Rik	protein_coding
ENSMUSG00000029283.17	1393.085	0.117	0.040	2.892	0.003825653	0.028863807	Cdc7	protein_coding
ENSMUSG00000040940.18	4651.912	-0.115	0.040	-2.892	0.003830462	0.028863807	Arhgef1	protein_coding
ENSMUSG00000000561.14	1490.969	0.126	0.043	2.892	0.003831652	0.028863807	Wdr77	protein_coding
ENSMUSG00000020538.15	3082.030	-0.138	0.048	-2.892	0.003831714	0.028863807	Srebf1	protein_coding
ENSMUSG00000029723.16	2949.047	-0.109	0.038	-2.890	0.003846367	0.028958708	Tsc22d4	protein_coding
ENSMUSG00000029462.18	4216.778	0.097	0.034	2.889	0.003869613	0.029118169	Vps29	protein_coding
ENSMUSG00000091956.2	32.221	-0.179	0.062	-2.889	0.003869925	NA	C2cd4b	protein_coding
ENSMUSG00000039163.10	310.748	0.194	0.067	2.888	0.003873526	0.029132059	Cmc1	protein_coding
ENSMUSG00000020986.13	5605.758	0.090	0.031	2.887	0.003892832	0.029261638	Sec23a	protein_coding
ENSMUSG00000024220.13	5710.187	-0.107	0.037	-2.886	0.003898454	0.029288282	Zfp523	protein_coding
ENSMUSG00000025266.11	11716.046	0.080	0.028	2.884	0.003920773	0.029440261	Gnl3l	protein_coding
ENSMUSG00000030960.16	664.343	0.155	0.054	2.882	0.003947563	0.029625642	Mettl10	protein_coding
ENSMUSG00000031813.8	775.359	-0.161	0.056	-2.882	0.003949961	0.029627858	Mvb12a	protein_coding
ENSMUSG00000018677.9	2573.863	-0.142	0.049	-2.881	0.003961619	0.029699499	Slc25a39	protein_coding
ENSMUSG00000061524.8	854.621	-0.209	0.072	-2.879	0.003988925	0.029866952	Zic2	protein_coding
ENSMUSG00000038039.13	4280.819	0.087	0.030	2.879	0.003989146	0.029866952	Gcc2	protein_coding

ENSMUSG00000024807.17	3449.735	-0.114	0.040	-2.879	0.003990313	0.029866952	Syvn1	protein_coding
ENSMUSG00000020358.17	4222.207	-0.111	0.038	-2.879	0.003994706	0.029883964	Hnrnpab	protein_coding
ENSMUSG00000041570.14	9699.306	0.071	0.025	2.878	0.003998301	0.02989499	Camsap2	protein_coding
ENSMUSG00000029381.15	273.473	-0.219	0.076	-2.878	0.004001258	0.029901232	Shroom3	protein_coding
ENSMUSG00000022559.7	1359.811	-0.131	0.045	-2.877	0.004017849	0.030009306	Fbxl6	protein_coding
ENSMUSG00000055471.6	437.764	-0.213	0.074	-2.875	0.004039517	0.030155167	Alk	protein_coding
ENSMUSG00000027274.16	1701.579	0.133	0.046	2.873	0.004060939	0.030299032	Mkks	protein_coding
ENSMUSG00000027122.15	1751.407	0.109	0.038	2.873	0.004066632	0.030325454	Arl14ep	protein_coding
ENSMUSG00000020090.7	228.748	-0.224	0.078	-2.872	0.004079794	0.03039211	Npffr1	protein_coding
ENSMUSG00000050796.4	483.713	-0.178	0.062	-2.872	0.004082039	0.03039211	B3galt6	protein_coding
ENSMUSG00000026219.14	12298.936	0.071	0.025	2.871	0.004093044	0.030457953	Trip12	protein_coding
ENSMUSG00000095224.1	33.520	-0.187	0.065	-2.870	0.004099328	NA	F930015N05Rik	protein_coding
ENSMUSG00000045896.14	4282.507	0.101	0.035	2.870	0.004101729	0.030506471	Paip2b	protein_coding
ENSMUSG00000073295.4	1365.631	0.121	0.042	2.870	0.004108414	0.030540078	Nudt11	protein_coding
ENSMUSG00000051951.5	2172.903	0.105	0.036	2.868	0.004126198	0.030656103	Xkr4	protein_coding
ENSMUSG00000030284.11	4354.857	-0.099	0.034	-2.866	0.004156924	0.030868116	Creld1	protein_coding
ENSMUSG00000055733.6	2486.578	0.106	0.037	2.865	0.00417592	0.03099215	Nap1l3	protein_coding
ENSMUSG00000041528.15	4775.163	-0.086	0.030	-2.864	0.004178273	0.03099215	Rnf123	protein_coding
ENSMUSG00000018809.2	683.226	0.159	0.056	2.864	0.004180726	0.03099215	Smyd4	protein_coding
ENSMUSG00000074102.4	3178.611	-0.097	0.034	-2.864	0.004182423	0.03099215	Rbm15b	protein_coding
ENSMUSG00000007659.18	5113.133	-0.080	0.028	-2.864	0.004186887	0.030993083	Bcl2l1	protein_coding
ENSMUSG00000047412.16	1323.609	0.148	0.052	2.864	0.004188874	0.030993083	Zbtb44	protein_coding
ENSMUSG00000053799.6	1417.664	0.135	0.047	2.864	0.004189146	0.030993083	Exoc6	protein_coding
ENSMUSG00000039375.16	1969.057	0.141	0.049	2.861	0.004220222	0.031198206	Wdr17	protein_coding
ENSMUSG00000027550.14	1081.901	0.130	0.045	2.861	0.004222124	0.031198206	Lrrcc1	protein_coding
ENSMUSG00000036908.16	699.321	-0.175	0.061	-2.861	0.004225796	0.031198206	Unc93b1	protein_coding
ENSMUSG00000061589.14	7852.959	-0.120	0.042	-2.861	0.004225844	0.031198206	Dot1l	protein_coding
ENSMUSG00000056073.16	4267.930	0.112	0.039	2.861	0.004227939	0.031198206	Grik2	protein_coding
ENSMUSG00000059981.12	9912.784	-0.099	0.035	-2.859	0.004256135	0.031387988	Taok2	protein_coding
ENSMUSG00000020102.15	2436.958	0.126	0.044	2.858	0.004258112	0.031387988	Slc16a7	protein_coding
ENSMUSG00000024943.8	1749.443	0.122	0.043	2.858	0.004262882	0.03140672	Smc5	protein_coding
ENSMUSG00000027335.9	1020.145	-0.223	0.078	-2.857	0.004282678	0.031536084	Adra1d	protein_coding
ENSMUSG00000039081.9	149.357	-0.221	0.077	-2.856	0.004288181	0.031560116	Zfp503	protein_coding

ENSMUSG00000029213.11	1603.446	0.119	0.042	2.855	0.004309594	0.031686385	Commd8	protein_coding
ENSMUSG00000038733.13	7341.153	0.068	0.024	2.855	0.004309834	0.031686385	Wdr26	protein_coding
ENSMUSG00000031917.12	990.362	0.140	0.049	2.854	0.00431307	0.031693643	Nip7	protein_coding
ENSMUSG00000067212.8	872.700	-0.188	0.066	-2.854	0.004318576	0.031717568	H2-T23	protein_coding
ENSMUSG00000031776.17	5533.946	-0.084	0.029	-2.854	0.004323844	0.031739714	Arl2bp	protein_coding
ENSMUSG00000081058.5	47.844	-0.207	0.073	-2.852	0.004342478	NA	Hist2h3c2	protein_coding
ENSMUSG00000042203.7	2451.620	-0.107	0.037	-2.852	0.004343863	0.03187007	Tbc1d22b	protein_coding
ENSMUSG00000025958.14	3011.460	0.096	0.034	2.851	0.00435359	0.031924816	Creb1	protein_coding
ENSMUSG00000039110.14	195.719	-0.220	0.077	-2.850	0.004365995	0.031999137	Mycbpap	protein_coding
ENSMUSG00000012640.16	1783.452	0.109	0.038	2.850	0.004370688	0.032016877	Zfp715	protein_coding
ENSMUSG00000024122.16	8309.132	0.074	0.026	2.849	0.004383893	0.032096926	Pdpk1	protein_coding
ENSMUSG00000038415.10	133.771	-0.221	0.078	-2.849	0.004392286	0.032141681	Foxq1	protein_coding
ENSMUSG00000000058.6	1457.141	0.134	0.047	2.847	0.004411064	0.032262345	Cav2	protein_coding
ENSMUSG00000062190.12	4117.026	0.100	0.035	2.847	0.00441492	0.032273801	Lancl2	protein_coding
ENSMUSG00000045980.13	1095.528	-0.154	0.054	-2.846	0.004420507	0.03229789	Tmem104	protein_coding
ENSMUSG00000026623.16	16016.804	0.074	0.026	2.845	0.004444631	0.032457323	Lpgat1	protein_coding
ENSMUSG00000003438.17	1207.996	-0.121	0.043	-2.843	0.004464717	0.03258712	Timm50	protein_coding
ENSMUSG00000034152.6	5909.334	0.090	0.031	2.843	0.004474989	0.032638421	Exoc3	protein_coding
ENSMUSG00000037818.16	1205.024	0.137	0.048	2.842	0.004476378	0.032638421	Abhd18	protein_coding
ENSMUSG00000033216.9	783.972	-0.150	0.053	-2.841	0.004492265	0.03273732	Eefsec	protein_coding
ENSMUSG00000025130.11	5505.445	-0.080	0.028	-2.840	0.004507173	0.032828994	P4hb	protein_coding
ENSMUSG00000024851.13	12086.263	-0.117	0.041	-2.837	0.004554349	0.033138355	Pitpnm1	protein_coding
ENSMUSG00000019539.11	167.063	-0.218	0.077	-2.837	0.004559046	0.033155412	Rcn3	protein_coding
ENSMUSG00000029408.13	2561.511	-0.128	0.045	-2.836	0.00456438	0.033177085	Abcb9	protein_coding
ENSMUSG00000016757.10	930.409	-0.136	0.048	-2.835	0.004576449	0.033247667	Ttll12	protein_coding
ENSMUSG00000037364.12	7777.017	-0.076	0.027	-2.835	0.004580435	0.033259483	Srrt	protein_coding
ENSMUSG00000027581.12	14149.775	-0.080	0.028	-2.834	0.004594615	0.03332125	Stmn3	protein_coding
ENSMUSG00000038366.15	9096.112	-0.087	0.031	-2.834	0.004595448	0.03332125	Lasp1	protein_coding
ENSMUSG00000029245.16	6123.933	0.112	0.040	2.834	0.004596034	0.03332125	Epha5	protein_coding
ENSMUSG00000014846.12	2026.408	-0.143	0.050	-2.833	0.00460418	0.033333759	Tppp3	protein_coding
ENSMUSG00000056258.8	9482.170	0.103	0.036	2.833	0.004604787	0.033333759	Kcnq3	protein_coding
ENSMUSG00000017286.15	2324.834	0.104	0.037	2.833	0.004604855	0.033333759	Glod4	protein_coding
ENSMUSG00000027248.13	9807.582	-0.108	0.038	-2.832	0.004619637	0.033423596	Pdia3	protein_coding

ENSMUSG00000034471.12	2006.459	-0.123	0.043	-2.832	0.004627907	0.033466247	Caskin2	protein_coding
ENSMUSG00000025425.17	2589.963	-0.103	0.036	-2.829	0.004669854	0.033752269	St8sia5	protein_coding
ENSMUSG00000030226.12	4956.855	0.155	0.055	2.828	0.004677427	0.033789673	Lmo3	protein_coding
ENSMUSG00000007670.9	5115.982	-0.097	0.034	-2.828	0.004682195	0.03380679	Khsrp	protein_coding
ENSMUSG00000071369.11	2636.207	-0.099	0.035	-2.828	0.004685263	0.033811622	Map3k5	protein_coding
ENSMUSG00000020570.15	2038.760	0.129	0.046	2.827	0.00469803	0.033886403	Sypl	protein_coding
ENSMUSG00000042662.16	1088.737	-0.130	0.046	-2.826	0.004715355	0.03399397	Dusp15	protein_coding
ENSMUSG00000031634.13	1845.088	0.120	0.042	2.825	0.004730072	0.03407211	Ufsp2	protein_coding
ENSMUSG00000035403.10	464.103	-0.186	0.066	-2.825	0.004731029	0.03407211	Crb2	protein_coding
ENSMUSG00000020591.11	4510.472	-0.084	0.030	-2.825	0.004734878	0.034082418	Ntsr2	protein_coding
ENSMUSG00000028033.16	4456.268	0.103	0.036	2.824	0.004741451	0.034099745	Kcnq5	protein_coding
ENSMUSG00000033671.18	4661.247	0.079	0.028	2.824	0.004742124	0.034099745	Cep350	protein_coding
ENSMUSG00000055884.8	736.958	0.146	0.052	2.822	0.004773188	0.034305613	Fancm	protein_coding
ENSMUSG00000037376.14	1310.073	-0.130	0.046	-2.822	0.004779664	0.034332589	Trmt6	protein_coding
ENSMUSG00000037509.19	11931.775	-0.080	0.028	-2.821	0.004783695	0.034332589	Arhgef4	protein_coding
ENSMUSG00000028518.8	2769.534	0.101	0.036	2.821	0.004784512	0.034332589	Prkaa2	protein_coding
ENSMUSG00000019970.15	4033.305	-0.219	0.078	-2.821	0.004786685	0.034332589	Sgk1	protein_coding
ENSMUSG00000091264.2	11522.341	0.082	0.029	2.821	0.004793677	0.034365248	Smim13	protein_coding
ENSMUSG00000024201.12	4733.544	-0.084	0.030	-2.820	0.004801317	0.034393205	Kdm4b	protein_coding
ENSMUSG00000042446.16	3214.860	0.089	0.032	2.820	0.004802457	0.034393205	Zmym4	protein_coding
ENSMUSG00000033728.14	926.267	-0.151	0.054	-2.819	0.004814755	0.034463769	Lrrc14	protein_coding
ENSMUSG00000039361.11	7728.129	0.090	0.032	2.818	0.004834198	0.034585377	Picalm	protein_coding
ENSMUSG00000036698.10	9195.413	-0.062	0.022	-2.816	0.004861759	0.034764904	ago-02	protein_coding
ENSMUSG00000028397.13	1301.415	0.138	0.049	2.815	0.004884951	0.034900442	Kdm4c	protein_coding
ENSMUSG00000054455.4	7009.041	-0.081	0.029	-2.814	0.004885666	0.034900442	Vapb	protein_coding
ENSMUSG00000033287.15	5947.733	-0.096	0.034	-2.814	0.004894437	0.034945388	Kctd17	protein_coding
ENSMUSG00000000776.12	803.949	-0.154	0.055	-2.814	0.004899679	0.034965105	Polr3d	protein_coding
ENSMUSG00000021290.6	1707.258	0.119	0.042	2.813	0.004905902	0.03499179	2010107E04Rik	protein_coding
ENSMUSG00000033430.10	2967.685	-0.095	0.034	-2.812	0.004916008	0.035046138	Terf2ip	protein_coding
ENSMUSG00000027613.15	1607.451	-0.126	0.045	-2.812	0.00492641	0.035102537	Eif6	protein_coding
ENSMUSG00000040813.16	2429.520	-0.105	0.037	-2.811	0.00494198	0.035178427	Tex264	protein_coding
ENSMUSG00000025658.16	12177.632	0.094	0.034	2.811	0.004942052	0.035178427	Cnksr2	protein_coding
ENSMUSG00000042286.13	1297.622	-0.160	0.057	-2.809	0.004961971	0.035302381	Stab1	protein_coding

ENSMUSG00000030528.12	229.010	0.199	0.071	2.809	0.004968199	0.035328857	Blm	protein_coding
ENSMUSG00000048279.17	5423.330	0.086	0.031	2.807	0.00500054	0.035540906	Sacs	protein_coding
ENSMUSG00000020954.16	3645.356	0.099	0.035	2.807	0.005006569	0.035558457	Strn3	protein_coding
ENSMUSG00000029061.2	149.090	-0.217	0.077	-2.807	0.005008056	0.035558457	Mmp23	protein_coding
ENSMUSG00000079084.10	1635.208	0.141	0.050	2.806	0.005015205	0.035591286	Ccdc82	protein_coding
ENSMUSG00000047446.18	1627.763	0.120	0.043	2.804	0.005046804	0.035790326	Arl4a	protein_coding
ENSMUSG00000018593.13	7566.220	-0.134	0.048	-2.804	0.00504907	0.035790326	Sparc	protein_coding
ENSMUSG00000002808.7	5220.543	-0.086	0.031	-2.804	0.00505087	0.035790326	Epdr1	protein_coding
ENSMUSG00000018474.17	40392.474	-0.081	0.029	-2.803	0.005057989	0.035813577	Chd3	protein_coding
ENSMUSG00000031715.14	2584.249	0.102	0.036	2.803	0.005059983	0.035813577	Smarca5	protein_coding
ENSMUSG00000079317.10	1364.598	0.126	0.045	2.803	0.005061774	0.035813577	Trappc2	protein_coding
ENSMUSG00000059669.8	905.192	0.143	0.051	2.803	0.005068909	0.03583094	Taf1b	protein_coding
ENSMUSG00000090112.8	3283.617	0.094	0.034	2.803	0.005069313	0.03583094	Shprh	protein_coding
ENSMUSG00000038084.16	8417.630	0.076	0.027	2.802	0.005076634	0.035861985	Opa1	protein_coding
ENSMUSG00000019817.18	1365.174	0.130	0.047	2.802	0.005078794	0.035861985	Plagl1	protein_coding
ENSMUSG00000024423.5	14629.599	0.087	0.031	2.801	0.005096157	0.035966571	Impact	protein_coding
ENSMUSG00000001143.13	3114.395	-0.105	0.038	-2.801	0.005100714	0.035978228	Lman2l	protein_coding
ENSMUSG00000049744.15	393.411	0.213	0.076	2.800	0.005102915	0.035978228	Arhgap15	protein_coding
ENSMUSG00000040875.12	2174.705	-0.115	0.041	-2.799	0.005120472	0.036083966	Osbpl10	protein_coding
ENSMUSG00000036315.13	428.333	0.170	0.061	2.799	0.005128896	0.036125266	Znrd1	protein_coding
ENSMUSG00000018848.4	1883.804	0.107	0.038	2.797	0.005151132	0.036263764	Rars	protein_coding
ENSMUSG00000040785.18	57443.698	0.049	0.017	2.797	0.005156703	0.036283546	Ttc3	protein_coding
ENSMUSG00000041891.15	1859.862	0.107	0.038	2.797	0.005160279	0.036283546	Lman1	protein_coding
ENSMUSG00000079657.10	3066.927	-0.121	0.043	-2.797	0.005161665	0.036283546	Rab26	protein_coding
ENSMUSG00000078234.6	705.399	-0.182	0.065	-2.796	0.0051724	0.036331245	Klhdc7a	protein_coding
ENSMUSG00000036242.14	1256.763	0.125	0.045	2.796	0.005173606	0.036331245	3632451O06Rik	protein_coding
ENSMUSG00000043843.16	1477.358	-0.160	0.057	-2.796	0.005178287	0.036346006	Tmem145	protein_coding
ENSMUSG00000027977.15	2952.218	0.112	0.040	2.795	0.005191014	0.036412328	Ndst3	protein_coding
ENSMUSG00000001383.8	5937.772	0.094	0.034	2.795	0.005195378	0.036412328	Zmat2	protein_coding
ENSMUSG00000032097.10	8456.316	0.081	0.029	2.794	0.005198277	0.036412328	Ddx6	protein_coding
ENSMUSG00000030682.4	6057.526	-0.079	0.028	-2.794	0.00520168	0.036412328	Cdipt	protein_coding
ENSMUSG00000020814.13	293.498	-0.207	0.074	-2.794	0.005203141	0.036412328	Mxra7	protein_coding
ENSMUSG00000028232.13	1629.116	0.121	0.043	2.794	0.005203237	0.036412328	Tmem68	protein_coding

ENSMUSG00000006423.15	1915.349	0.107	0.038	2.794	0.005210308	0.036443715	C330007P06Rik	protein_coding
ENSMUSG00000022443.16	7353.311	-0.118	0.042	-2.792	0.005230901	0.036569602	Myh9	protein_coding
ENSMUSG00000040396.12	1884.509	0.102	0.037	2.791	0.005249978	0.036684775	Abhd13	protein_coding
ENSMUSG00000022382.14	2139.972	-0.128	0.046	-2.791	0.005257933	0.036722157	Wnt7b	protein_coding
ENSMUSG00000015605.5	3418.427	-0.122	0.044	-2.790	0.00526776	0.036759408	Srf	protein_coding
ENSMUSG00000020078.16	2662.592	0.124	0.044	2.790	0.005268483	0.036759408	Vps26a	protein_coding
ENSMUSG00000033389.16	7933.180	-0.089	0.032	-2.788	0.00529651	0.03692171	Arhgap44	protein_coding
ENSMUSG00000016256.10	1407.544	-0.121	0.043	-2.788	0.005296984	0.03692171	Ctsz	protein_coding
ENSMUSG00000031837.14	2895.485	-0.151	0.054	-2.787	0.005316324	0.037038196	Necab2	protein_coding
ENSMUSG00000009633.3	144.464	-0.217	0.078	-2.787	0.005322191	0.037060144	G0s2	protein_coding
ENSMUSG00000019055.15	1263.282	-0.163	0.059	-2.787	0.005324733	0.037060144	Plod1	protein_coding
ENSMUSG00000032087.10	4527.800	-0.114	0.041	-2.786	0.00532979	0.03707703	Dscaml1	protein_coding
ENSMUSG00000024193.7	2505.588	-0.115	0.041	-2.786	0.005333565	0.037084987	Phf1	protein_coding
ENSMUSG00000039716.12	18783.837	0.068	0.024	2.786	0.005338257	0.037099307	Dock3	protein_coding
ENSMUSG00000052915.14	9299.328	-0.066	0.024	-2.785	0.005352187	0.037177783	Msl1	protein_coding
ENSMUSG00000020042.15	1637.831	-0.117	0.042	-2.785	0.005357703	0.037197769	Btbd11	protein_coding
ENSMUSG00000074698.10	3945.323	0.082	0.029	2.781	0.005419775	0.037610194	Csnk2a1	protein_coding
ENSMUSG00000040007.8	2720.039	-0.127	0.046	-2.779	0.005452442	0.037818268	Bahd1	protein_coding
ENSMUSG00000071252.5	483.675	0.166	0.060	2.779	0.005456501	0.037827805	2210408I21Rik	protein_coding
ENSMUSG00000042599.8	4016.723	0.112	0.040	2.777	0.005486038	0.038013876	Kdm7a	protein_coding
ENSMUSG00000029860.16	2184.588	-0.105	0.038	-2.776	0.005509872	0.038160264	Zyx	protein_coding
ENSMUSG00000040520.7	1643.982	0.125	0.045	2.775	0.005517989	0.03819771	Manea	protein_coding
ENSMUSG00000041445.8	245.744	-0.215	0.077	-2.775	0.005522707	0.0382116	Mmrn2	protein_coding
ENSMUSG00000020053.18	365.508	0.191	0.069	2.775	0.00552578	0.038214106	Igf1	protein_coding
ENSMUSG00000052397.8	3327.604	-0.115	0.041	-2.774	0.005541827	0.038306284	Ezr	protein_coding
ENSMUSG00000041729.15	8383.494	-0.092	0.033	-2.774	0.00554493	0.03830894	Coro2b	protein_coding
ENSMUSG00000025743.14	12097.867	-0.112	0.040	-2.772	0.005576636	0.03850912	Sdc3	protein_coding
ENSMUSG00000029401.8	496.165	-0.171	0.062	-2.770	0.00559875	0.038634996	Rilpl2	protein_coding
ENSMUSG00000042404.16	3734.127	-0.093	0.033	-2.770	0.005600347	0.038634996	Dennd4b	protein_coding
ENSMUSG00000047368.3	1283.838	0.130	0.047	2.770	0.005609163	0.038676883	Abhd17b	protein_coding
ENSMUSG00000073725.8	9290.077	0.072	0.026	2.769	0.005617691	0.038716744	Lmbrd1	protein_coding
ENSMUSG00000021774.12	2554.221	0.100	0.036	2.769	0.005628913	0.03877512	Ube2e1	protein_coding
ENSMUSG00000063260.2	302.870	0.197	0.071	2.768	0.005633208	0.038785754	Syt10	protein_coding

ENSMUSG00000050910.5	1115.463	-0.134	0.048	-2.768	0.005638877	0.038794395	Cdr2l	protein_coding
ENSMUSG00000004771.12	3830.992	0.084	0.030	2.768	0.005639968	0.038794395	Rab11a	protein_coding
ENSMUSG00000055567.18	24243.610	0.075	0.027	2.767	0.005654084	0.038872518	Unc80	protein_coding
ENSMUSG00000035293.13	1037.761	0.157	0.057	2.767	0.005661781	0.038906456	G2e3	protein_coding
ENSMUSG00000035840.6	399.358	0.173	0.063	2.766	0.005669875	0.038943089	Lysmd3	protein_coding
ENSMUSG00000019173.11	5180.158	-0.076	0.027	-2.765	0.005685611	0.039025495	Rab5c	protein_coding
ENSMUSG00000054934.10	3130.202	-0.122	0.044	-2.765	0.005687686	0.039025495	Kcnmb4	protein_coding
ENSMUSG00000021039.9	3336.649	0.092	0.033	2.765	0.00569018	0.039025495	Snw1	protein_coding
ENSMUSG00000026825.17	65705.309	-0.072	0.026	-2.764	0.005715101	0.039177351	Dnm1	protein_coding
ENSMUSG00000029674.13	3929.279	-0.110	0.040	-2.763	0.005728089	0.039243635	Limk1	protein_coding
ENSMUSG00000042178.6	675.698	-0.160	0.058	-2.763	0.005730339	0.039243635	Armc5	protein_coding
ENSMUSG00000040249.15	40651.053	-0.097	0.035	-2.763	0.005735388	0.039254812	Lrp1	protein_coding
ENSMUSG00000023143.10	514.390	-0.184	0.066	-2.762	0.005737542	0.039254812	Nagpa	protein_coding
ENSMUSG00000024045.5	6238.589	0.079	0.029	2.761	0.005755902	0.039361321	Akap8	protein_coding
ENSMUSG00000016526.8	420.795	-0.179	0.065	-2.760	0.005779913	0.0394872	Dyrk3	protein_coding
ENSMUSG00000006095.12	2408.184	-0.097	0.035	-2.760	0.005788023	0.039523449	Tbcb	protein_coding
ENSMUSG00000024381.15	12840.022	-0.083	0.030	-2.759	0.005806029	0.03961539	Bin1	protein_coding
ENSMUSG00000035314.10	2094.494	-0.121	0.044	-2.758	0.005807109	0.03961539	Gdpd5	protein_coding
ENSMUSG00000033813.15	1668.487	0.108	0.039	2.758	0.00581374	0.039641436	Tcea1	protein_coding
ENSMUSG00000066647.6	1585.416	0.114	0.041	2.757	0.005827577	0.039716574	Gm5113	protein_coding
ENSMUSG00000026834.13	1244.311	0.137	0.050	2.757	0.005833485	0.039737623	Acvr1c	protein_coding
ENSMUSG00000045036.14	124.341	0.215	0.078	2.756	0.005845567	0.039800685	Tmem232	protein_coding
ENSMUSG00000035284.10	8268.370	0.080	0.029	2.756	0.005854228	0.039840407	Vps13c	protein_coding
ENSMUSG00000024137.8	2338.670	-0.115	0.042	-2.755	0.005863247	0.039882529	E4f1	protein_coding
ENSMUSG00000013155.10	387.411	-0.182	0.066	-2.755	0.005874729	0.039941353	Enkd1	protein_coding
ENSMUSG00000027708.14	1468.397	0.122	0.044	2.754	0.005887303	0.039988263	Dcun1d1	protein_coding
ENSMUSG00000001552.14	3210.399	-0.116	0.042	-2.753	0.005906036	0.040093642	Jup	protein_coding
ENSMUSG00000037989.15	10860.162	-0.102	0.037	-2.753	0.005911169	0.040093642	Wnk2	protein_coding
ENSMUSG00000008305.18	1931.676	-0.119	0.043	-2.753	0.005911352	0.040093642	Tle1	protein_coding
ENSMUSG00000050697.8	1417.420	0.125	0.046	2.751	0.005948761	0.040314754	Prkaa1	protein_coding
ENSMUSG00000024172.10	2834.719	0.108	0.039	2.751	0.005949673	0.040314754	St6gal2	protein_coding
ENSMUSG00000033615.9	21615.743	-0.083	0.030	-2.750	0.005952727	0.040316064	Cplx1	protein_coding
ENSMUSG00000025008.15	1934.860	0.102	0.037	2.750	0.005956814	0.040324372	Tctn3	protein_coding

ENSMUSG00000068551.12	1088.754	-0.138	0.050	-2.750	0.005961202	0.040334697	Zfp467	protein_coding
ENSMUSG00000069631.14	1626.706	0.123	0.045	2.749	0.00597548	0.040411909	Strada	protein_coding
ENSMUSG00000025790.14	2271.448	-0.118	0.043	-2.748	0.005987484	0.040473672	Slco3a1	protein_coding
ENSMUSG00000021832.7	3314.268	0.092	0.033	2.748	0.005998556	0.040529071	Psmc6	protein_coding
ENSMUSG00000049285.4	99.117	-0.214	0.078	-2.748	0.006004683	0.04055103	Mblac1	protein_coding
ENSMUSG00000047604.3	212.635	-0.199	0.072	-2.747	0.006007727	0.040552159	Frat2	protein_coding
ENSMUSG00000039782.14	3982.258	0.110	0.040	2.745	0.006048589	0.04080843	Cpeb2	protein_coding
ENSMUSG00000032397.7	333.840	0.188	0.069	2.744	0.00606369	0.040881917	Tipin	protein_coding
ENSMUSG00000039578.16	477.317	0.178	0.065	2.744	0.006065282	0.040881917	Ccser1	protein_coding
ENSMUSG00000054752.16	2312.713	0.101	0.037	2.742	0.006103073	0.041116976	Fsd1l	protein_coding
ENSMUSG00000001496.15	62.217	-0.190	0.069	-2.742	0.006109188	NA	Nkx2-1	protein_coding
ENSMUSG00000038530.11	15289.276	0.084	0.031	2.742	0.006112611	0.041161555	Rgs4	protein_coding
ENSMUSG00000027488.12	1497.250	-0.132	0.048	-2.741	0.006124783	0.041223825	Snta1	protein_coding
ENSMUSG00000027955.16	208.751	0.208	0.076	2.739	0.006159335	0.041436593	Fam198b	protein_coding
ENSMUSG00000018481.9	5367.124	0.084	0.031	2.739	0.00616238	0.041437298	Appbp2	protein_coding
ENSMUSG00000037820.15	877.950	-0.198	0.072	-2.737	0.006199061	0.041664073	Tgm2	protein_coding
ENSMUSG00000049612.10	4633.062	0.085	0.031	2.735	0.006234373	0.041869309	Omg	protein_coding
ENSMUSG00000021848.15	82.083	-0.167	0.061	-2.735	0.006235539	0.041869309	Otx2	protein_coding
ENSMUSG00000094114.1	413.993	0.204	0.075	2.734	0.006248919	0.041939176	Gm21967	protein_coding
ENSMUSG00000059839.8	826.854	0.152	0.056	2.734	0.006263913	0.042016847	Zfp874b	protein_coding
ENSMUSG00000073792.11	624.500	0.158	0.058	2.734	0.006266455	0.042016847	Alg6	protein_coding
ENSMUSG00000074165.10	1768.465	0.128	0.047	2.733	0.006275422	0.042056968	Zfp788	protein_coding
ENSMUSG00000047648.13	412.804	0.177	0.065	2.732	0.006286652	0.042112204	Fbxo30	protein_coding
ENSMUSG00000022983.15	2360.445	-0.110	0.040	-2.732	0.006291571	0.042125132	Scaf4	protein_coding
ENSMUSG00000066735.8	3528.781	0.090	0.033	2.732	0.006302111	0.042175665	Vkorc1l1	protein_coding
ENSMUSG00000060639.5	52.900	-0.201	0.074	-2.731	0.006314248	NA	Hist1h4i	protein_coding
ENSMUSG00000027810.14	1679.940	0.132	0.048	2.730	0.006334064	0.042369384	Eif2a	protein_coding
ENSMUSG00000033849.3	3236.461	0.125	0.046	2.730	0.006341409	0.042398394	B3galt2	protein_coding
ENSMUSG00000005034.15	28854.178	0.061	0.022	2.729	0.006355942	0.042475414	Prkacb	protein_coding
ENSMUSG00000042851.17	553.603	0.152	0.056	2.728	0.006369746	0.04254749	Zc3h6	protein_coding
ENSMUSG00000033055.10	815.844	-0.136	0.050	-2.728	0.006374	0.042555737	Ankrd54	protein_coding
ENSMUSG00000026269.14	2295.990	-0.114	0.042	-2.727	0.006398805	0.042701115	Rnpepl1	protein_coding
ENSMUSG00000044477.10	3343.999	-0.118	0.043	-2.726	0.0064047	0.042706017	Zfand3	protein_coding

ENSMUSG00000024420.8	717.480	-0.196	0.072	-2.726	0.0064056	0.042706017	Zfp521	protein_coding
ENSMUSG00000021171.8	3538.054	0.091	0.033	2.726	0.006413177	0.042725841	Esyt2	protein_coding
ENSMUSG00000074129.13	235.171	-0.195	0.072	-2.726	0.006414636	0.042725841	Rpl13a	protein_coding
ENSMUSG00000041014.17	1717.614	0.123	0.045	2.726	0.006419917	0.042740816	Nrg3	protein_coding
ENSMUSG00000024560.6	2503.614	-0.103	0.038	-2.725	0.006427504	0.042771122	Cxxc1	protein_coding
ENSMUSG00000014444.17	663.923	-0.180	0.066	-2.725	0.006433626	0.042791657	Piezo1	protein_coding
ENSMUSG00000030401.16	3844.811	-0.124	0.046	-2.724	0.006456423	0.042922783	Rtn2	protein_coding
ENSMUSG00000015656.17	65683.816	-0.083	0.030	-2.723	0.006459431	0.042922783	Hspa8	protein_coding
ENSMUSG00000031916.17	1122.218	-0.126	0.046	-2.723	0.006465627	0.04294371	Cog8	protein_coding
ENSMUSG00000021037.7	4742.147	-0.080	0.029	-2.722	0.006487034	0.043065593	Ahsa1	protein_coding
ENSMUSG00000043448.13	547.355	-0.208	0.076	-2.717	0.006582017	0.043674573	Gjc2	protein_coding
ENSMUSG00000089911.4	2239.967	0.113	0.041	2.717	0.006584963	0.043674573	Mfsd14a	protein_coding
ENSMUSG00000031150.12	792.878	-0.136	0.050	-2.717	0.006591749	0.043699014	Ccdc120	protein_coding
ENSMUSG00000032788.15	12161.495	-0.102	0.038	-2.716	0.006615343	0.043834808	Pdxk	protein_coding
ENSMUSG00000040648.14	1916.818	0.125	0.046	2.715	0.006626085	0.043885356	Ppip5k2	protein_coding
ENSMUSG00000006154.13	408.391	-0.167	0.062	-2.715	0.006635473	0.043926896	Eps8l1	protein_coding
ENSMUSG00000027009.18	851.224	0.190	0.070	2.714	0.006641903	0.043948819	Itga4	protein_coding
ENSMUSG00000043683.3	2939.697	-0.105	0.039	-2.714	0.006651113	0.043989107	Fem1a	protein_coding
ENSMUSG00000020614.13	831.777	-0.149	0.055	-2.713	0.006672281	0.044108409	Fam20a	protein_coding
ENSMUSG00000024777.9	6707.032	-0.097	0.036	-2.711	0.006698487	0.04426089	Ppp2r5b	protein_coding
ENSMUSG00000032171.7	2803.766	-0.109	0.040	-2.711	0.006706056	0.044290135	Pin1	protein_coding
ENSMUSG00000026767.12	1645.453	0.110	0.041	2.708	0.006771409	0.044700812	Fam188a	protein_coding
ENSMUSG00000036907.9	43.696	-0.191	0.071	-2.706	0.006803759	NA	C1ql2	protein_coding
ENSMUSG00000026648.18	872.248	0.142	0.052	2.705	0.006821518	0.045010522	Dclre1c	protein_coding
ENSMUSG00000025321.14	2513.808	0.110	0.041	2.705	0.006832186	0.045059816	Itgb8	protein_coding
ENSMUSG00000007216.6	292.151	-0.190	0.070	-2.704	0.006841802	0.045074542	Zfp775	protein_coding
ENSMUSG00000014859.9	1489.180	-0.115	0.042	-2.704	0.006844013	0.045074542	E2f4	protein_coding
ENSMUSG00000045107.4	278.928	-0.196	0.072	-2.703	0.006878541	0.045280786	Saysd1	protein_coding
ENSMUSG00000068039.12	8061.654	0.082	0.031	2.702	0.006882277	0.045284225	Tcp1	protein_coding
ENSMUSG00000008226.14	1345.839	0.125	0.046	2.702	0.006889846	0.045312871	Scrn3	protein_coding
ENSMUSG00000026610.13	1123.516	0.149	0.055	2.702	0.006899454	0.045349659	Esrrg	protein_coding
ENSMUSG00000049878.13	2161.002	0.105	0.039	2.702	0.006901874	0.045349659	Rlf	protein_coding
ENSMUSG00000052364.4	1371.834	-0.131	0.049	-2.701	0.006911133	0.045389334	B630019K06Rik	protein_coding

ENSMUSG00000042410.15	2344.567	0.107	0.040	2.701	0.006922701	0.04544413	Agps	protein_coding
ENSMUSG00000030513.14	1121.150	-0.136	0.050	-2.700	0.006936232	0.04548413	Pcsk6	protein_coding
ENSMUSG00000045509.6	193.486	-0.198	0.073	-2.700	0.006938222	0.04548413	Gpr150	protein_coding
ENSMUSG00000091735.2	290.588	-0.204	0.076	-2.700	0.006938476	0.04548413	Gpr62	protein_coding
ENSMUSG00000038967.13	5227.473	-0.088	0.033	-2.699	0.006947404	0.045521485	Pdk2	protein_coding
ENSMUSG00000059878.12	967.968	0.125	0.046	2.699	0.006952317	0.045532504	Zfp422	protein_coding
ENSMUSG00000005262.12	3552.824	0.088	0.033	2.699	0.006964035	0.04558807	Ufd1	protein_coding
ENSMUSG00000038615.17	18271.517	-0.058	0.022	-2.698	0.006976156	0.045646216	Nfe2l1	protein_coding
ENSMUSG00000028496.17	1575.401	0.120	0.045	2.697	0.006990241	0.045674758	Mllt3	protein_coding
ENSMUSG00000027111.15	1265.562	0.126	0.047	2.696	0.007008464	0.045772609	Itga6	protein_coding
ENSMUSG00000060904.14	5263.984	0.079	0.029	2.693	0.007072686	0.046170653	Arl1	protein_coding
ENSMUSG00000032570.17	8878.145	0.073	0.027	2.693	0.007090744	0.046267107	Atp2c1	protein_coding
ENSMUSG00000016382.15	10739.546	0.104	0.039	2.692	0.007103226	0.046327102	Pls3	protein_coding
ENSMUSG00000036916.13	1515.935	0.142	0.053	2.692	0.007109242	0.046344895	Zfp280c	protein_coding
ENSMUSG00000036790.5	1309.742	0.123	0.046	2.691	0.007117785	0.046379134	Slitrk2	protein_coding
ENSMUSG00000026281.15	1166.070	-0.125	0.046	-2.691	0.007125334	0.046406869	Dtymk	protein_coding
ENSMUSG00000039910.10	1138.082	-0.140	0.052	-2.691	0.007134382	0.04643725	Cited2	protein_coding
ENSMUSG00000037720.16	4761.924	0.077	0.029	2.690	0.007136589	0.04643725	Tmem33	protein_coding
ENSMUSG00000024012.17	15839.944	-0.106	0.039	-2.690	0.007140933	0.046444076	Mtch1	protein_coding
ENSMUSG00000068740.13	17826.844	-0.075	0.028	-2.689	0.007167096	0.046592738	Celsr2	protein_coding
ENSMUSG00000058748.9	390.564	0.184	0.069	2.687	0.007206343	0.046810091	Zfp958	protein_coding
ENSMUSG00000019689.4	370.163	-0.170	0.063	-2.687	0.007207173	0.046810091	Fmc1	protein_coding
ENSMUSG00000011877.13	19549.112	-0.092	0.034	-2.686	0.007233572	0.046922564	Git1	protein_coding
ENSMUSG00000031393.16	9061.807	0.062	0.023	2.686	0.007234478	0.046922564	Mecp2	protein_coding
ENSMUSG00000032409.15	1125.174	0.157	0.058	2.686	0.007237827	0.046922692	Atr	protein_coding
ENSMUSG00000041548.4	597.647	-0.173	0.065	-2.684	0.00728405	0.047197763	Hspb8	protein_coding
ENSMUSG00000053347.14	1087.190	0.134	0.050	2.683	0.007287066	0.047197763	Zfp943	protein_coding
ENSMUSG00000044067.6	2387.410	0.124	0.046	2.683	0.007290303	0.047197763	Gpr22	protein_coding
ENSMUSG00000031099.16	2363.641	0.121	0.045	2.683	0.007306212	0.047279043	Smarca1	protein_coding
ENSMUSG00000037857.16	2184.582	0.120	0.045	2.682	0.007314549	0.047311267	Nufip2	protein_coding
ENSMUSG00000027287.14	962.019	0.132	0.049	2.682	0.007322486	0.04734088	Snap23	protein_coding
ENSMUSG00000034973.16	4085.498	0.084	0.031	2.681	0.007339583	0.047429655	Dopey1	protein_coding
ENSMUSG00000078185.3	895.546	0.133	0.050	2.680	0.007361616	0.047550239	Chml	protein_coding

ENSMUSG00000002617.14	1165.963	0.140	0.052	2.679	0.007378464	0.047637232	Zfp40	protein_coding
ENSMUSG00000062619.5	58.950	-0.200	0.075	-2.679	0.00737943	NA	2310039H08Rik	protein_coding
ENSMUSG00000038831.16	6307.480	0.078	0.029	2.678	0.007400811	0.047759632	Ralgps1	protein_coding
ENSMUSG00000029405.16	16300.226	0.070	0.026	2.678	0.007408037	0.047784383	G3bp2	protein_coding
ENSMUSG00000033282.14	1762.898	0.108	0.040	2.677	0.007422698	0.047844895	Rpgrip1l	protein_coding
ENSMUSG00000030629.15	2268.933	0.106	0.039	2.677	0.007424208	0.047844895	Zfand6	protein_coding
ENSMUSG00000028322.11	274.590	-0.194	0.072	-2.677	0.007439339	0.047920492	Exosc3	protein_coding
ENSMUSG00000023883.13	3234.964	0.089	0.033	2.676	0.007451321	0.04797575	Phf10	protein_coding
ENSMUSG00000027201.16	4202.662	0.086	0.032	2.676	0.007458014	0.047996918	Myef2	protein_coding
ENSMUSG00000002043.17	227.930	-0.188	0.070	-2.675	0.007482645	0.048133453	Trappc6a	protein_coding
ENSMUSG00000028161.17	41244.093	0.082	0.031	2.674	0.007487491	0.048142655	Ppp3ca	protein_coding
ENSMUSG00000050321.2	3482.175	0.093	0.035	2.674	0.007499394	0.048197199	Neto1	protein_coding
ENSMUSG00000039270.9	6770.498	0.086	0.032	2.674	0.007503347	0.048200628	Megf9	protein_coding
ENSMUSG00000047731.17	2050.544	-0.101	0.038	-2.673	0.007512855	0.048217751	Wbp1l	protein_coding
ENSMUSG00000063785.12	966.427	0.128	0.048	2.673	0.007517067	0.048222826	Utp14a	protein_coding
ENSMUSG00000032410.13	2476.364	0.091	0.034	2.672	0.007540384	0.04832841	Xrn1	protein_coding
ENSMUSG00000010914.10	1946.506	0.110	0.041	2.670	0.007574058	0.048522167	Pdhx	protein_coding
ENSMUSG00000061118.8	1274.726	-0.130	0.049	-2.670	0.007582972	0.048557206	Dnajc30	protein_coding
ENSMUSG00000057421.12	3418.129	0.091	0.034	2.669	0.007613474	0.048730379	Las1l	protein_coding
ENSMUSG00000014932.15	574.521	0.145	0.054	2.668	0.007636795	0.048857461	Yes1	protein_coding
ENSMUSG00000003099.11	5552.623	-0.088	0.033	-2.667	0.007647231	0.048889144	Ppp5c	protein_coding
ENSMUSG00000042804.13	1206.048	-0.178	0.067	-2.667	0.007649974	0.048889144	Gpr153	protein_coding
ENSMUSG00000044066.14	1914.024	0.110	0.041	2.667	0.007652154	0.048889144	Cep68	protein_coding
ENSMUSG00000003299.10	1900.200	-0.114	0.043	-2.667	0.007662792	0.048934928	Mrpl4	protein_coding
ENSMUSG00000010406.11	445.176	-0.172	0.065	-2.666	0.007674969	0.048982569	Mrpl52	protein_coding
ENSMUSG00000042197.13	3407.182	0.092	0.034	2.666	0.007680858	0.048982569	Zfp451	protein_coding
ENSMUSG00000036437.6	1293.833	0.140	0.052	2.666	0.007682872	0.048982569	Npy1r	protein_coding
ENSMUSG00000036615.6	962.322	-0.142	0.053	-2.666	0.007684154	0.048982569	Rfxap	protein_coding
ENSMUSG00000018446.8	1737.771	-0.119	0.045	-2.663	0.007737277	0.049298907	C1qbp	protein_coding
ENSMUSG00000034659.14	2439.170	-0.129	0.049	-2.663	0.007750776	0.049362599	Tmem109	protein_coding
ENSMUSG00000036257.15	6555.799	0.080	0.030	2.661	0.007794153	0.049594034	Pnpla8	protein_coding
ENSMUSG00000024054.13	2452.193	0.099	0.037	2.658	0.00785059	0.0499306	Smchd1	protein_coding
ENSMUSG00000020034.7	1302.962	0.122	0.046	2.658	0.007859044	0.049961827	Tcp11l2	protein_coding

Supplementary Table S3. Gene expression changes in mPFC (FDR 5%) in Plcb1+/- mice compared to WT after cocaine seeking reinstatement

ids	baseMean	log2FoldChange	lfcSE	stat	pvalue	padj	Gene Name	Gene type
ENSMUSG00000022769.7	906.219	-0.242	0.039	-6.25	4.22E-10	9.05E-06	Sdf2l1	protein_coding
ENSMUSG00000026864.13	25343.092	-0.156	0.034	-4.57	4.82E-06	0.021	Hspa5	protein_coding
ENSMUSG00000038393.14	2084.497	-0.162	0.034	-4.75	2.03E-06	0.021	Txnip	protein_coding
ENSMUSG00000040046.14	69.482	-0.088	0.019	-4.60	4.19E-06	0.021	Tph1	protein_coding
ENSMUSG00000021025.8	960.708	-0.178	0.039	-4.63	3.68E-06	0.021	Nfkbia	protein_coding
ENSMUSG00000027342.14	1321.497	-0.156	0.036	-4.31	1.62E-05	0.035	Pcna	protein_coding
ENSMUSG00000003814.8	21535.466	-0.128	0.029	-4.34	1.40E-05	0.035	Calr	protein_coding
ENSMUSG00000005958.14	1125.700	0.155	0.036	4.31	1.61E-05	0.035	Ephb3	protein_coding
ENSMUSG00000024966.8	8739.320	-0.115	0.026	-4.38	1.19E-05	0.035	Stip1	protein_coding
ENSMUSG00000067608.4	81.222	0.061	0.014	4.40	1.08E-05	0.035	Pcna-ps2	protein_coding
ENSMUSG00000004951.10	382.579	-0.156	0.037	-4.23	2.32E-05	0.042	Hspb1	protein_coding
ENSMUSG00000096768.7	184.618	-0.146	0.034	-4.24	2.28E-05	0.042	Erdr1	protein_coding

Supplementary Table S4. Enrichment of GO (Gene Ontology) terms in differentially expressed genes in mPFC

Term	Genes	Count	%	P-Value	Benjamini
Type I pneumocyte differentiation		6	0,3	2,2E-4	3,3E-2
regulation of alpha-amino-3-hydroxy-5-methyl-4-isoxazole propionate selective glutamate receptor activity		10	0,5	3,0E-5	6,9E-3
mRNA splice site selection		11	0,5	2,8E-7	2,7E-4
positive regulation of excitatory postsynaptic potential		11	0,5	3,4E-4	4,8E-2
neurotransmitter secretion		13	0,6	1,0E-5	3,5E-3
dendrite morphogenesis		15	0,7	7,5E-5	1,4E-2
long-term synaptic potentiation		15	0,7	3,5E-4	4,8E-2
synapse organization		16	0,7	1,9E-6	9,0E-4
learning		19	0,9	1,0E-4	1,9E-2
response to endoplasmic reticulum stress		23	1,0	6,5E-6	2,6E-3
regulation of cell cycle		26	1,2	1,9E-4	3,0E-2
axonogenesis		28	1,3	1,1E-5	3,4E-3
rhythmic process		28	1,3	2,9E-4	4,2E-2
regulation of translation		30	1,4	1,5E-5	4,2E-3
peptidyl-serine phosphorylation		30	1,4	9,5E-5	1,8E-2
neuron projection development		32	1,5	3,5E-5	7,7E-3
actin cytoskeleton organization		33	1,5	2,1E-5	5,4E-3
ubiquitin-dependent protein catabolic process		35	1,6	1,9E-5	5,1E-3
chemical synaptic transmission		35	1,6	1,9E-4	3,0E-2
endocytosis		40	1,8	8,7E-6	3,3E-3
cell-cell adhesion		40	1,8	2,5E-5	6,1E-3
brain development		48	2,2	8,2E-7	5,0E-4
RNA splicing		54	2,5	1,2E-7	1,4E-4
positive regulation of apoptotic process		58	2,6	1,5E-4	2,5E-2
mRNA processing		65	3,0	3,1E-7	2,5E-4
nervous system development		85	3,9	1,2E-11	2,9E-8
protein phosphorylation		90	4,1	1,0E-4	1,8E-2
apoptotic process		91	4,1	4,3E-5	8,7E-3
protein transport		98	4,5	4,6E-6	2,0E-3
phosphorylation		99	4,5	1,1E-5	3,4E-3

negative regulation of transcription from RNA polymerase II promoter	120	5,5	4,8E-7	3,3E-4
positive regulation of transcription from RNA polymerase II promoter	144	6,5	4,3E-5	9,0E-3
transport	252	11,4	1,5E-6	8,1E-4
transcription, DNA-templated	302	13,7	3,9E-15	1,9E-11
regulation of transcription, DNA-templated	335	15,2	1,2E-11	2,0E-8

Supplementary Table S5. Enrichment of KEGG (Kyoto Encyclopedia of Genes and Genomes) terms in differentially expressed genes in mPFC

Terms	Genes	Count	%	P-Value	Benjamini
Endocytosis		56	2,5	1,2E-7	3,2E-5
Protein processing in endoplasmic reticulum		40	1,8	1,2E-7	1,6E-5
MAPK signaling pathway		52	2,4	1,9E-7	1,7E-5
mTOR signaling pathway		20	0,9	1,3E-6	8,8E-5
Ubiquitin mediated proteolysis		33	1,5	3,3E-6	1,8E-4
Amphetamine addiction		19	0,9	4,1E-5	1,8E-3
Synaptic vesicle cycle		18	0,8	5,0E-5	1,9E-3
Nicotine addiction		14	0,6	5,6E-5	1,9E-3
Long-term potentiation		18	0,8	1,2E-4	3,5E-3
FoxO signaling pathway		28	1,3	1,5E-4	4,0E-3
Neurotrophin signaling pathway		26	1,2	2,0E-4	4,8E-3
B cell receptor signaling pathway		18	0,8	2,6E-4	5,7E-3
Spliceosome		27	1,2	3,3E-4	6,7E-3
Estrogen signaling pathway		22	1,0	3,3E-4	6,4E-3
Renal cell carcinoma		17	0,8	4,7E-4	8,4E-3
Choline metabolism in cancer		22	1,0	5,1E-4	8,6E-3
Insulin signaling pathway		27	1,2	7,5E-4	1,2E-2
Colorectal cancer		16	0,7	8,7E-4	1,3E-2
Glioma		16	0,7	1,0E-3	1,4E-2
Chronic myeloid leukemia		17	0,8	1,1E-3	1,5E-2
Axon guidance		25	1,1	1,1E-3	1,5E-2
ErbB signaling pathway		19	0,9	1,3E-3	1,6E-2
VEGF signaling pathway		15	0,7	1,3E-3	1,6E-2
Prostate cancer		19	0,9	1,5E-3	1,7E-2
Insulin resistance		22	1,0	1,6E-3	1,7E-2
Dopaminergic synapse		25	1,1	2,0E-3	2,0E-2
Non-alcoholic fatty liver disease (NAFLD)		28	1,3	2,0E-3	1,9E-2
HTLV-I infection		43	2,0	2,0E-3	1,9E-2
cAMP signaling pathway		33	1,5	2,1E-3	1,9E-2
Oxytocin signaling pathway		28	1,3	2,2E-3	1,9E-2
Amyotrophic lateral sclerosis (ALS)		13	0,6	2,7E-3	2,3E-2
Pancreatic cancer		15	0,7	3,0E-3	2,5E-2
Endometrial cancer		13	0,6	3,2E-3	2,6E-2
Progesterone-mediated oocyte maturation		18	0,8	3,4E-3	2,7E-2
Osteoclast differentiation		23	1,0	4,1E-3	3,1E-2
Focal adhesion		33	1,5	4,6E-3	3,4E-2
Acute myeloid leukemia		13	0,6	6,1E-3	4,3E-2
Signaling pathways regulating pluripotency of stem cells		24	1,1	6,1E-3	4,2E-2
Hepatitis B		25	1,1	6,2E-3	4,2E-2
Melanoma		15	0,7	6,9E-3	4,5E-2
Central carbon metabolism in cancer		14	0,6	7,0E-3	4,5E-2
Adipocytokine signaling pathway		15	0,7	7,9E-3	4,9E-2
Epstein-Barr virus infection		33	1,5	8,1E-3	4,9E-2

Supplementary Table S6. Correspondence between genes and proteins from the Dopaminergic synapse from KEGG pathways

Protein in Figure 4	Gene Symbol	Gene Name	Entrez Gene ID	RNAseq FC
Akt	Akt1	thymoma viral proto-oncogene 1	11651	-1.068
	Akt2	thymoma viral proto-oncogene 2	11652	-1.088
	Akt3	thymoma viral proto-oncogene 3	23797	1.052
Cav2.1/2.2 and Vgcc	Cacna1a	calcium channel, voltage-dependent, P/Q type, alpha 1A subunit	12286	-1.093
Cam	Calm2	calmodulin 2	12314	1.092
	Calm3	calmodulin 3	12315	-1.079
Comt	Comt	catechol-O-methyltransferase	12846	-1.091
Creb	Atf6b	activating transcription factor 6 beta	12915	-1.084
	Creb1	cAMP responsive element binding protein 1	12912	1.069
Gi/o	Gnao1	guanine nucleotide binding protein, alpha O	14681	-1.051
	Gng2	guanine nucleotide binding protein (G protein), gamma 2	14702	1.104
Ampar	Gria2	glutamate receptor, ionotropic, AMPA2 (alpha 2)	14800	1.133
	Gria3	glutamate receptor, ionotropic, AMPA3 (alpha 3)	53623	1.090
	Gria4	glutamate receptor, ionotropic, AMPA4 (alpha 4)	14802	1.070
Girk	Kcnj3	potassium inwardly-rectifying channel, subfamily J, member 3	16519	1.103
Mao	Maoa	monoamine oxidase A	17161	1.115
Mapk	Mapk8	mitogen-activated protein kinase 8	26419	1.088
	Mapk9	mitogen-activated protein kinase 9	26420	1.065
Pp1	Ppp1cb	protein phosphatase 1 catalytic subunit beta	19046	1.152
Pp2a	Ppp2r3d	protein phosphatase 2 (formerly 2A), regulatory subunit B'', delta	19054	-1.213
	Ppp2r5b	protein phosphatase 2, regulatory subunit B', beta	225849	-1.070
Pp2b	Ppp3ca	protein phosphatase 3, catalytic subunit, alpha isoform	19055	1.058
	Ppp3cb	protein phosphatase 3, catalytic subunit, beta isoform	19056	1.134
Pka	Prkacb	protein kinase, cAMP dependent, catalytic, beta	18749	1.043
Vssc	Scn1a	sodium channel, voltage-gated, type I, alpha	20265	1.097

FC= Fold-change from RNAseq of mPFC comparing Plcb1+/- and WT mice. In red, genes upregulated. In green, genes downregulated.

Supplementary Table S6. Enrichment of transcription factor binding sites (TFBS) in genes differentially expressed in mPFC

TFBS	Size	Expect	Observed	Ratio	P Value	FDR
GCCATNTTG_YY1_Q6	672	78.886	Size	1.724	6.20E-11	1.38E-08
MAZR_01	368	43.199	87	2.0139	7.31E-11	1.38E-08
YGCGYRCGC_UNKNOWN	459	53.882	102	1.893	7.91E-11	1.38E-08
GCANCTGNY_MYOD_Q6	1475	173.15	252	1.4554	9.15E-11	1.38E-08
AP2ALPHA_01	380	44.608	88	1.9727	1.78E-10	2.16E-08
CTGCAGY_UNKNOWN	1236	145.09	216	1.4887	3.31E-10	3.34E-08
TGCGCANK_UNKNOWN	804	94.381	153	1.6211	4.41E-10	3.81E-08
CACBINDINGPROTEIN_Q6	397	46.604	89	1.9097	8.20E-10	6.20E-08
RCGCANGCGY_NRF1_Q6	1263	148.26	216	1.4569	2.34E-09	1.57E-07
EGR1_01	429	50.36	90	1.7871	2.07E-08	1.25E-06
AP2GAMMA_01	382	44.843	82	1.8286	3.07E-08	1.69E-06
ERR1_Q2	414	48.599	86	1.7696	6.82E-08	3.32E-06
TCF1P_Q6	421	49.421	87	1.7604	7.32E-08	3.32E-06
EGR_Q6	396	46.486	83	1.7855	7.68E-08	3.32E-06
VDR_Q3	366	42.964	78	1.8155	9.17E-08	3.70E-06
TGACCTY_ERR1_Q2	1551	182.07	247	1.3566	1.11E-07	4.20E-06
FAC1_01	344	40.382	73	1.8077	2.82E-07	1.00E-05
RNGTGGGC_UNKNOWN	1164	136.64	192	1.4051	3.09E-07	1.04E-05
CCCNNGGGAR_OLF1_01	521	61.16	100	1.6351	3.47E-07	1.11E-05
PAX4_01	408	47.895	82	1.7121	5.86E-07	1.77E-05
WTTGKCTG_UNKNOWN	846	99.311	146	1.4701	6.85E-07	1.97E-05
RTAAACA_FREAC2_01	1379	161.88	218	1.3467	1.2171E-06	3.18E-05
AAAYRNCTG_UNKNOWN	596	69.964	109	1.5579	1.2211E-06	3.18E-05
LFA1_Q6	370	43.434	75	1.7268	1.2629E-06	3.18E-05
AR_01	246	28.878	55	1.9046	1.5287E-06	3.62E-05
GATA1_01	411	48.247	81	1.6789	1.5557E-06	3.62E-05
PAX4_03	452	53.06	87	1.6397	1.7888E-06	3.93E-05
YY1_Q6	406	47.66	80	1.6786	1.8168E-06	3.93E-05
TCCATTKW_UNKNOWN	388	45.547	77	1.6906	2.1371E-06	4.46E-05
ZF5_B	383	44.96	76	1.6904	2.4956E-06	5.03E-05
NGFIC_01	399	46.838	78	1.6653	3.3359E-06	6.43E-05
FOXJ2_02	373	43.786	74	1.69	3.4025E-06	6.43E-05
MYOD_01	466	54.703	88	1.6087	3.5165E-06	6.45E-05
TFIII_Q6	335	39.325	68	1.7292	3.7303E-06	6.64E-05
TGCTGAY_UNKNOWN	952	111.75	157	1.4049	4.1129E-06	6.98E-05
YY1_02	336	39.443	68	1.724	4.1526E-06	6.98E-05
EGR2_01	317	37.212	65	1.7467	0.000004276	6.99E-05
MTF1_Q4	435	51.064	83	1.6254	4.3921E-06	6.99E-05
TTCYRGAA_UNKNOWN	523	61.395	96	1.5637	4.5215E-06	7.01E-05
CATTGTYY_SOX9_B1	586	68.79	105	1.5264	4.9945E-06	7.40E-05
GATTGGY_NFY_Q6_01	1839	215.88	274	1.2692	6.9387E-06	8.90E-05
TGACATY_UNKNOWN	1034	121.38	167	1.3758	7.0622E-06	8.90E-05
CDP_02	220	25.826	48	1.8586	0.000013889	1.56E-04
EGR3_01	149	17.491	36	2.0582	0.000016232	1.70E-04
SOX9_B1	377	44.256	72	1.6269	0.000018087	1.77E-04
GTGACGY_E4F1_Q6	965	113.28	155	1.3683	0.00002078	1.96E-04

TGACCTTG_SF1_Q6	391	45.899	73	1.5904	0.000034911	2.97E-04
KCCGNSWTTT_UNKNOWN	150	17.608	35	1.9877	0.000046306	3.74E-04
OCT1_04	355	41.673	67	1.6077	0.00005124	4.08E-04
HSF_Q6	356	41.791	67	1.6032	0.00005605	4.29E-04
HEN1_01	317	37.212	61	1.6392	0.000061522	4.65E-04
AP1_Q4_01	446	52.356	79	1.5089	0.00010872	7.39E-04
NRF2_Q4	428	50.243	76	1.5127	0.00013453	8.84E-04
TGACAGNY_MEIS1_01	1337	156.95	198	1.2616	0.0002177	1.24E-03
CACGTG_MYC_Q2	1558	182.89	226	1.2357	0.00026579	1.45E-03
E2F_Q2	288	33.808	54	1.5973	0.00031278	1.66E-03
RGAANNTTC_HSF1_01	767	90.038	120	1.3328	0.00052592	2.59E-03
OCT_Q6	457	53.647	77	1.4353	0.00063826	2.93E-03
CATRRAGC_UNKNOWN	239	28.056	45	1.6039	0.00086894	3.70E-03
E4BP4_01	406	47.66	69	1.4478	0.0009417	3.90E-03
AP1_Q2	385	45.195	66	1.4603	0.00095563	3.93E-03
CDPCR1_01	221	25.943	42	1.6189	0.001049	4.12E-03
ZIC3_01	430	50.477	72	1.4264	0.0011215	4.32E-03
CREB_01	422	49.538	70	1.413	0.0016763	6.05E-03
ER_Q6_01	408	47.895	68	1.4198	0.0017129	6.13E-03
RFX1_01	416	48.834	69	1.413	0.001805	6.39E-03
FOXO1_01	385	45.195	64	1.4161	0.0024536	7.88E-03
E2F_02	414	48.599	68	1.3992	0.0024883	7.88E-03
MYOD_Q6_01	457	53.647	73	1.3608	0.0036598	0.010544
CREB_Q2	465	54.586	74	1.3557	0.0037971	0.010785
SP1_Q6	395	46.369	64	1.3802	0.0045082	0.012341
HIF1_Q5	388	45.547	63	1.3832	0.0045782	0.012421
NRF2_01	403	47.308	65	1.374	0.0047014	0.012586
FOXO3_01	367	43.082	60	1.3927	0.0047836	0.012749
RP58_01	353	41.438	58	1.3997	0.0049148	0.013015
NKX25_02	413	48.482	66	1.3613	0.0054775	0.014162
RORA1_01	436	51.182	68	1.3286	0.0085232	0.019385
NF1_Q6_01	449	52.708	69	1.3091	0.011251	0.024396
AR_Q2	188	22.069	33	1.4953	0.011569	0.024733
E2F_Q3_01	376	44.138	59	1.3367	0.012024	0.025615
HNF3_Q6	289	33.925	47	1.3854	0.012637	0.026639
DR1_Q3	371	43.551	58	1.3318	0.013637	0.028254
TFIIA_Q6	431	50.595	66	1.3045	0.013943	0.02879
ETS_Q4	391	45.899	60	1.3072	0.017663	0.034584
GTTRYCATRR_UNKNOWN	277	32.517	44	1.3531	0.02247	0.042088
TAL1ALPHAE47_01	404	47.425	61	1.2862	0.022922	0.04267
RSRFC4_01	452	53.06	67	1.2627	0.025666	0.04663
